# Illuminating the mechanism: gene expression responses to antimicrobial photodynamic therapy

**DOI:** 10.1186/s12864-026-12676-0

**Published:** 2026-02-23

**Authors:** Natalia Burzyńska, Agata Woźniak-Pawlikowska, Aleksandra Rapacka-Zdończyk, Maciej Jaśkiewicz, Beata Kruszewska-Naczk, Joanna Nakonieczna, Michał Wojciech Szcześniak, Mariusz Grinholc

**Affiliations:** 1 Laboratory of Photobiology and Molecular Diagnostics, Intercollegiate Faculty of Biotechnology, University of Gdansk and Medical University of Gdansk, Gdansk, Poland; 2 Department of Bioinformatics, Institute of Human Biology and Evolution, Adam Mickiewicz University in Poznan, Poznan, Poland

**Keywords:** aPDI, aBL, gene expression, RNA-seq, RT-qPCR

## Abstract

Photodynamic treatments are promising antimicrobial approaches that rely on the generation of reactive oxygen species (ROS), which lead to damage to bacterial cell structures. As the number of studies investigating gene expression changes in microorganisms following antimicrobial photodynamic inactivation (aPDI) and antimicrobial blue light (aBL) exposure is steadily increasing, there is a clear need for a comprehensive review that synthesizes these findings. This article aims to address this gap by presenting the current state of knowledge on transcriptomic alterations induced by photodynamic therapies, with a particular focus on studies utilizing qPCR and RNA-seq methodologies. Investigated genes were divided into key functional categories associated with virulence factors, DNA repair, detoxification and oxidative stress response, defense mechanisms and cellular metabolism. A general trend toward reduced expression of virulence-related genes has been observed, indicating the potential of these methods not only in eradicating bacteria but also in reducing their pathogenicity. Increased expression of certain genes linked with oxidative stress response and heat shock proteins may reflect adaptive or compensatory survival mechanisms. Differences in experimental results are often due to different protocols regarding the type of photosensitizer, wavelength of light or phase of bacterial growth.

**Supplementary Information:**

The online version contains supplementary material available at 10.1186/s12864-026-12676-0.

## Introduction

Antimicrobial resistance (AMR) represents one of the most significant public health and modern medicine challenges in Europe and around the world today. This “silent pandemic” was classified to the top ten global threats till the COVID-19 outbreak [1]. Unfortunately, the COVID-19 pandemic has put tremendous strain on healthcare systems. It led to a redirection of resources, personnel, and attention away from AMR surveillance to favor SARS-CoV-2-oriented detection [2]. Even more concerning, the overuse of antibiotics and antimicrobial stewardship neglect during a pandemic [3, 4] very likely exacerbated the AMR crisis. On top of that, the global antibiotic pipeline has been jeopardized, largely because of inadequate funding for creating new antibiotics and restricted global access to both new and existing ones [5]. To address antimicrobial resistance, there is a need for multi-sector collaboration to enhance local and global policies and education on antibiotic use, strengthen surveillance systems, and advance new technologies and alternative antimicrobial treatments [6].

Antimicrobial photodynamic inactivation (aPDI) and antimicrobial blue light (aBL) have been gaining much more attention as tools against bacterial pathogens, especially in the context of a possible upcoming “post-antibiotic” era. The efficacy of aPDI [7–9] and aBL [10–12] toward pathogenic microorganisms has been widely proven, regardless of their antibiotic resistance profile. The assumed mechanism of action of both photodynamic methods involves the generation of reactive oxygen species (ROS) upon activation of the photosensitizer (PS) by light, with important distinctions in the origin, localization, and effects of these PSs. aPDI is based on the use of exogenously administered photosensitizers, i.e., chemical compounds that can be excited with a wavelength of light that overlaps with the absorption spectra of PSs [13]. In contrast, aBL exploits naturally occurring endogenous porphyrins in bacterial cells that can absorb blue light in the range of 400–470 nm [14]. Illumination of either exogenous or endogenous PSs in aerobic conditions induces the generation of singlet oxygen (Type II reaction) and/or other reactive oxygen species like hydrogen peroxide, superoxide anion, and hydroxyl radicals (Type I reaction) [15]. In the case of aPDI, the relative contribution of these photodynamic mechanisms is unique for each photosensitizing compound and depends on its chemical structure [15], whereas excitation of endogenous porphyrins is generally associated with the generation of singlet oxygen, with other ROS produced secondarily via the Type I pathway [16]. These ROS induce cell membrane damage, DNA oxidation, as well as lipid and protein degradation [13, 17, 18]; however, the exact effects are strongly dependent on PS localization. For PSs that surround or bind to bacterial cells, the destructive effects are limited to outer structures such as the cell wall or membrane, whereas for internalized or endogenous photosensitizing compounds, damage is concentrated on intracellular components, including cytoplasmic proteins or DNA, ultimately leading to bacterial cell death. Moreover, Type III of the photoinactivation mechanism has been described as an oxygen-independent reaction in which activated PSs directly affect target molecules [19].

While the efficacy of photodynamic methods in pathogen inactivation has been well-documented, the molecular responses of microbial cells, particularly in terms of gene expression, remain not fully understood. This work provides a critical overview of current studies on gene expression changes following aPDI or aBL treatment, with emphasis on genes involved in virulence, DNA repair, defense, bacterial metabolism, and stress responses, utilizing qPCR and RNA-seq approaches. These findings will provide insight into the cellular adaptations driven by light-based antimicrobial methods, which can contribute to the development of aPDI/aBL as effective, routine tools for combating drug-resistant pathogens.

## Approaches for assessing gene expression changes in aPDI and aBL studies

Changes in gene expression induced by aPDI and aBL have been predominantly investigated using RNA sequencing (RNA-seq) [20–22] and quantitative PCR (qPCR) [23–25]. RNA-seq enables unbiased, transcriptome-wide profiling and is particularly useful for identifying global stress responses and pathways affected by photodynamic treatments, whereas qPCR is most commonly applied for targeted validation of selected genes. In recent years, additional approaches, including long-read RNA sequencing, digital PCR, and emerging single-cell transcriptomic techniques, have expanded the methodological toolbox for studying bacterial responses to photodynamic stress. Each of these methods differs in throughput, sensitivity, and suitability under oxidative stress conditions characteristic of aPDI and aBL experiments. A comparative overview of gene expression profiling techniques relevant to photodynamic studies is provided in Table 1.

**Table 1:** Comparison of gene expression analysis methods relevant to aPDI/aBL studies

Method / Feature	qPCR	RNA-seq (short read)	Long read RNA-seq (ONT/PacBio)	dPCR	scRNA-seq
Targeting	Known genes	Transcriptome-wide	Transcriptome-wide	Known genes	Transcriptome-wide
Quantification	Relative	Relative	Relative	Absolute	Relative
Novel transcript detection	No	Yes	Yes	No	Yes
Sensitivity	High	High	Moderate-high	Very high	Low-moderate
Single-cell compatible	No	No	No	No	Yes
Key strengths in aPDI/aBL studies	Rapid; cost-effective; high precision; suitable for validation of selected targets	Unbiased genome-wide profiling; high sensitivity across a wide dynamic range	Full-length transcript and operon resolution; improved identification of transcript boundaries	Absolute quantification without reference genes; high precision for low-abundance targets	Resolution of population heterogeneity; identification of subpopulations
Major limitations	Requires prior gene knowledge; dependence on stable reference genes under oxidative stress	Cost and computational complexity; snapshot of transcription; limited structural information	Protocol complexity; lower throughput; higher error rates	Limited multiplexing; one or few targets per assay	Not yet routine; technically demanding; low bacterial mRNA content
Applications in aPDI/aBL	Validation of RNA-seq results; targeted analysis of stress-response or resistance-associated genes	Global transcriptomic profiling; pathway discovery; comparative analysis of photodynamic responses	Emerging analysis of operon organization and transcript integrity after photodynamic stress	Potential quantification of tolerant- or survival-associated transcripts after repeated sublethal treatments	Conceptual or future analysis of heterogeneity in photodynamic responses
References	[26, 27]	[28, 29]	[30, 31]	[32, 33]	[34, 35]

## Virulence factors

The ability of bacteria to adapt and survive in hostile environments is largely dependent on the regulation of virulence-associated genes. Numerous studies have demonstrated that both aPDI and aBL can affect a wide spectrum of virulence factors in bacteria at the biological level. This chapter aims to address changes in the transcriptional level of genes encoding virulence factors, especially those associated with quorum sensing, biofilm formation, motility, secretion systems, as well as enterotoxin production.

### Quorum sensing and biofilm

Quorum sensing (QS) is a process of bacterial cell-to-cell communication involving the production, recognition, and response to extracellular signaling molecules known as autoinducers. It regulates cellular metabolic activity and modulates biofilm formation and virulence-associated traits, with the direction of changes largely depending on bacterial species and environmental conditions. In the case of biofilms, as a consortium of bacterial cells embedded in a self-produced matrix of extracellular polymeric substances (EPSs), the QS system regulates the formation of specialized biofilm structures as well as the expression of surface adhesion molecules [36–38]. Hence, interference with this signaling has been widely explored as an antimicrobial strategy that influences bacterial collective behaviors, with aPDI/aBL known for its ability to modulate biofilm formation.

In Gram-negative bacteria, genes related to QS and biofilm formation have been frequently investigated following photodynamic treatments. In *Pseudomonas aeruginosa*, Tan et al. reported that 5-aminolevulinic acid photodynamic therapy with red light reduced mRNA expression levels of QS-related genes involved in the synthesis of signaling molecules (*lasI*, *rhlI*) and transcription regulation (*lasR*,* rhlR*) to less than 0.5-fold compared with untreated cells, as well as cells exposed to light or ALA alone. However, this study reported growth inhibition rather than a reduction in bacterial viability [39]. The same QS genes were investigated in two independent studies, following exposure of the reference [40, 41] and clinical isolates [41], to the same conditions of methylene blue (MB) and red light (1–3 log_10_ decrease in viability). All tested genes showed reduced expression across all strains by approximately 1.5- to 2-fold [40], but discrepancies were observed for the reference strain between studies. In the second one, expression of genes was more diminished for *rhlR* (> 100-fold), *rhlI* (> 10-fold), and *lasR* (> 10-fold) [40]. They also observed a reduction of transcript levels for biofilm formation gene *pslA* (> 10-fold), biofilm biosynthesis glycosyltransferase gene *pelF* (> 10-fold) [40] and rhamnolipid gene *rhlA* (approx. 2-fold) [41]. Moreover, the MB and red light were applied to examine the transcription levels of *pqsA*, involved in quinolone signal production, *cdrA* encoding fibrillar adhesin, and *rhlA* at 1 (< 1 log_10_ reduction in viability), 2 (< 1 log_10_), and 5 (1–3 log_10_) min time points. Interestingly, expression levels of *cdrA* depended on the time of irradiation (downregulated 5.7-fold at 1 min; upregulated 7.1-fold at 2 min and 8.4-fold at 5 min), while for *rhlA* and *pqsA* genes, no significant changes were observed at any time [25]. mRNA levels of genes *pelA* (Pel polysaccharide production) and *pslA* (Psl polysaccharide production) were also analyzed in biofilm cultures under the influence of carbon dots (CDs) derivatives synthesized from gentamicin and imipenem and UVA light, affecting the viability of all tested strains by > 3 log_10_. Shiralizadeh et al. reported diminished expression of both genes for all tested PSs in comparison to untreated cells, i.e., *pelA* (> 50-fold for CDsGEN-NH2; approx. 14-fold for CDs-IMP-NH2; > 100-fold for CDs-IMP-GEN; approx. 100-fold for CDs-GEN-IMP) and *pslA* (approx. 100-fold, except for CDs-IMP-NH2 - approx. 20-fold) [42].

While the studies described above focused on single-species biofilms, aPDI effects on QS-system gene expression were also examined in a multi-species biofilm. Indocyanine green (ICG) and near-infrared light were applied to biofilm consisting of *P. aeruginosa*, *Acinetobacter baumannii*, and *S. aureus*, which resulted in downregulation of acyl-homoserine-lactone synthases (*lasI*, *abaI*) and staphylococcal accessory gene regulator A (*agrA*) to approximately 4.9-, 1.9-, and 3.7-fold, respectively. However, the applied treatment did not result in a statistically significant reduction in viability [43].

In the case of *A. baumannii* and acyl homoserine lactone synthase (*abaI*), which plays an important role in the QS system and biofilm formation, two independent studies analyzed its transcript levels after photodynamic treatment with exogenous PSs. Hypericin nanoparticles conjugated with D-amino acids (HypNP@D-Trp) combined with blue light resulted in a > 3 log_10_ reduction in viability [44], whereas for treatment with toluidine blue O (TBO) and red light, only growth inhibition was reported [45]. Notably, both approaches reported decreased *abaI* transcripts levels; however, the magnitude of change was not correlated with antimicrobial efficacy, i.e., a 4.64-fold decrease for HypNP@D-Trp-aPDI and 6.3- and 8.3-fold reduction for TBO-aPDI. Moreover, for TBO-aPDI, significant downregulation of additional biofilm-associated genes was noted, including the *csuE* gene encoding one of the most prominent *A. baumannii* adhesins (12- and 34-fold, strain dependent), and the *epsA* gene coding a polysaccharide export outer membrane protein (11.4- and 17.6-fold, strain dependent) [45]. Beyond photodynamic inactivation, two independent studies examined the expression of the *abaI* and *csu* genes following blue light irradiation. RT-qPCR analysis demonstrated that 462 nm light resulted in approximately 38-fold lower *abaI* expression at 23 °C and 1.5-fold higher expression at 37 °C in comparison to non-treated cells [46]. In addition, blue light exposure led to an approximately 1.45-fold decrease in transcripts of the Csu fimbrial major subunit - *csuAB*, which encodes pili critical for biofilm formation [47]. Transcriptome-wide analysis further supported a regulatory role of blue light in *A. baumannii*. RNA-seq revealed repression of a light-regulated locus encoding a predicted type I pilus involved in biofilm generation, named *prpABCD* for this study, with these findings subsequently confirmed by RT-qPCR (*prpA* 2.75-fold, *prpB* 4.41-fold, *prpC* 3.08-fold, and *prpD* 5.76-fold) [48]. Consistently, reduced expression of *fimA*, *fimC*, and *fimD* genes (2-2.83-fold) encoding type I fimbriae was observed following blue light exposure, as demonstrated by whole transcriptome analysis [49].

Among Gram-negative oral pathogens, photodynamic modulation of QS- and biofilm-associated genes has been extensively investigated in *Aggregatibacter actinomycetemcomitans*, a key etiological agent of periodontal disease. Several studies have focused on the expression of *rcpA*, a gene involved in adhesion and interactions with host epithelial cells. In a mixed-species biofilm model, photodynamic treatment using graphene quantum dots with curcumin (GQD-Cur) and blue light (< 1 log_10_ viability decrease) significantly reduced transcript levels of *rcpA*, as well as *fimA* in *Porphyromonas gingivalis* that encodes fimbriae, and *inpA* in *Prevotella intermedia*, a cysteine protease (8.1-, 9.6-, and 11.8-fold, respectively) [50]. In a related study focusing on *P. gingivalis*, reduced expression of *fimA* was observed following sublethal aPDI with MB (4.6-fold), TBO (14.4-fold), and ICG (17.3-fold) [51]. Notably, these transcriptional changes were detected at doses that did not result in a statistically significant reduction in bacterial viability. In *A. actinomycetemcomitans*, the expression level of the *rcpA* gene after aPDI was investigated in five more studies. Across these studies, downregulation of *rcpA* was consistently observed following photodynamic treatment; however, the magnitude of transcript reduction did not correlate with antimicrobial efficacy. Specifically, *rcpA* expression was reduced by 8.5-fold following curcumin-mediated aPDI with blue light without a statistically significant reduction in viability [52]. For curcumin-loaded nanoparticles with blue light (> 3 log_10_), the decrease was 4.4-, 5.2-, and 9.7-fold, with greater suppression observed at higher photosensitizer concentrations [53]. MB with red light [54], and ICG with near-infrared light [55], both caused < 1 log_10_ reduction in viability, reduced *rcpA* expression by 3.83- and 5.65-fold, respectively. In another study, ICG or ICG doped with chitosan nanoparticles and near-infrared light resulted in 6.4- and 13.2-fold downregulation, but corresponding viability data were not provided [56]. Moreover, for Cur-loaded nanoparticles-mediated aPDI, *qseB* and *qseC* genes, components of the QseBC two-component system in *A. actinomycetemcomitans*, exhibited lower expression in comparison to non-treated cells with an average decrease by 4.5- and 5.5-fold, respectively [53].

For some bacterial species, only single reports exist regarding the expression of genes related to biofilm or QS after aPDI or aBL. In *Serratia marcescens*, aPDI with MB and red light applied in sublethal (1–3 log_10_) and lethal (> 3 log_10_) doses led to the downregulation of QS-controlled biofilm formation genes (*bsmA* and *bsmB*, approx. 2.5–10 fold), fimbrial adhesion genes (*fimA* and *fimC*, approx. 3-10-fold), and QS gene *swrR* (approx. 4–10 fold), depending on the strain [57]. In *Escherichia coli*, blue light caused a < 1 log_10_ reduction in viability but induced a strong downregulation of curli biosynthesis genes (*csgABCD*, near detection limit, approx. ≥10³–10⁴-fold), as well as *fimA* (256-fold) and *fimH* (2048-fold) encoding type I pili, as confirmed by RT-qPCR [58]. While in *Cronobacter sakazakii*, also subjected to aBL, treatment resulted in a < 1 log₁₀ reduction in viability, and a moderate (approx. 4-fold) downregulation of *bcsA* and *bcsG*, cellulose biosynthesis genes, and QS-associated transcriptional regulator *luxR* was observed [59].

As a key Gram-positive oral pathogen, *Streptococcus mutans* has been extensively investigated in the context of its virulence and gene expression under different aPDI conditions. One of the most frequently examined genes was the *gtf* family, encoding glucosyltransferases, which are fundamental enzymes in adherence and cariogenic biofilm formation [60]. The transcript level of the *gtfB* has been investigated in seven independent studies, five of which were conducted by the Pourhajibagher research group. For all tested combinations, they reported significant downregulation of that gene, but the magnitude of change was rather PS-specific than dose-dependent. aPDI with TBO or ICG with red light resulted in 3.9- and 8.25-fold reduction in *gtfB* expression, respectively, despite no statistically significant decrease in bacterial viability [60]. In biofilm cultures exposed to photodynamic treatment (< 1 log_10_) with riboflavin [61], emodin-chitosan nanoparticles [62], and *Ulva lactuca* extract [63], all combined with blue light, *gtfB* transcript levels were reduced by 5.1-, 7.8-, and 6.4-fold, respectively. In contrast, nano‑quercetin excited by blue light, caused the highest reduction in viability (1–3 log_10_) with a 6.5-fold decrease at 64 µg/mL and a 4.1-fold decrease at 32 µg/mL, indicating a concentration-dependent transcriptional response [64]. Beyond this particular gene, the expression of other glucosyltransferase-encoding genes has been investigated, revealing that *Ulva lactuca*-mediated aPDI (< 1 log_10_) significantly downregulated *gtfC* and *gtfD* by 5.3- and 7.4-fold, respectively [63]. Moreover, genes involved in the QS system in *S. mutans*, i.e., *comA* and *comB* (ABC transporter complex), *comDE* (histidine kinase of the competence regulon and competence protein), exhibited lower mRNA levels (by approximately 3.2-4.1-fold, 4.08-5.05-fold, and 3.56-4.0-fold) in comparison to the untreated cells, after treatment with nano-quercetin and blue light (1–3 log_10_) [64]. Contrary to those findings, the research of Misba et al. reported a slight increase in expression of genes engaged in biofilm formation after TBO treatment (1–3 log_10_), i.e., genes encoding glucosyltransferase *gtfC* (1.2-fold), fructosyltransferases *ftf* (1.3-fold), glucan binding protein *gbpB* (1.7-fold), surface protein *spaP* (1.2-fold), two-component regulatory system *vicR* (1.3-fold), and *comDE* (1.3-fold). Remarkably, the silver conjugates of this PS led to a > 3 log_10_ decrease in viability and the downregulation of the expression of all tested genes, i.e., 1.7-, 1.5, 2-, 1.25-, 1.7-, and 1.4-fold, respectively [65]. An interesting research was carried out by Hosseinpour-Nader et al., where *gtfB* expression was estimated in *S. mutans* samples collected from patients subjected to daily administration of Cur and teeth irradiation with blue light. Swab samples collected on days: 30, 60, 90, and 120 revealed a progressive reduction in cells viability (< 1 log_10_, 1–3 log_10_, 1–3 log_10_, and > 3 log_10_, respectively), together with a corresponding decrease in glucosyltransferase B mRNA levels, reaching approximately 2.5-, 5.0-, 8.0-, and 15-fold downregulation, respectively [66]. Finally, RT-qPCR analysis demonstrated a 3.5-fold downregulation of *gtfB* following aPDI with red light and a mixture of phycocyanin (PC) and chlorophyllin (Chl), PhotoActive+. However, the applied treatment did not result in a statistically significant reduction in bacterial viability [67].

Changes in biofilm-associated gene expression following photodynamic treatment have also been investigated in *Staphylococcus aureus*, a Gram-positive representative of the ESKAPE group. Mahmoudi et al. tested a combination of TBO and red light at a dose that resulted in no statistically significant decrease in viability in three strains to determine the transcript levels of genes from the *icaABCD* operon involved in the synthesis of polysaccharide intercellular adhesin (PIA) and *icaR*, its repressor. The expression of all tested genes, i.e., *icaA*, *icaB*, *icaC*, *icaD*, and *icaR* for each strain, was significantly downregulated, with an average decrease of 12.5-, 14-, 11.5-, 9-, and 7-fold change, respectively [68]. An independent study performed by Yang et al. assessed the effect of both aPDI (< 1 log_10_) and aBL (1–3 log_10_) treatments on the expression of the *lrgA* gene, which indirectly affects biofilm production through regulation of the cell wall and synthesis of biofilm matrix components. Pooled results of RNA-seq and qPCR pointed out that hyprocrellin B-mediated aPDI increased mRNA level by approximately 5-fold, while aBL led to a decrease of *lrgA* transcript by approximately 1.45-fold [69].

Gene expression patterns after photodynamic treatment have been extensively studied in *Enterococcus faecalis*, especially in terms of genes involved in biofilm formation or QS, like *ace* (collagen adhesin), *gel*E (gelatinase), *esp* (Enterococcal surface protein), *efa* (Enterococcus faecalis antigen A), and *fsrBC* (QS system). In the case of the *esp* gene, a significantly lower expression was noted for ICG conjugated with iron or aluminum along with near-infrared light, resulting in a < 1 log_10_ decrease in viability. Depending on the PS, i.e., Fe-88-ICG, Fe-101-ICG, and Al-101-ICG, observed downregulation was 4.4-, 6.2- and 6.0-fold, respectively [70]. TBO, MB, and ICG irradiated with red and near-infrared light led to a decrease in transcript levels of *esp* by 3.2-, 2.8-, and 5.2-fold; however, data about reduced bacterial viability are not provided [71]. Additionally, reduced graphene oxide-curcumin (rGO-Cur) and blue light treatment resulted in approximately 23.0-fold downregulation; however, this study reported growth inhibition rather than a reduction in bacterial viability. Moreover, they noted diminished levels of *efa* (approx. 18-fold), *gelE* (approx. 8.5-fold), and *fsrC* (approx. 8.0-fold) [72]. The same three genes were investigated under conditions of ICG and near-infrared light (< 1 log_10_), and RT-qPCR analysis demonstrated significant downregulation of all tested genes, by 2.1-, 3.5-, and 4.1-fold, respectively [73]. Subsequent studies examining *esp* and *ace* expression demonstrated a significant decrease in transcript levels following treatment with aPDI. For Kojic acid and Parietin-mediated treatment, reduced expression of *ace* (3.75- and 5.32-fold, respectively) and *esp* (4.44- and 6.06-fold, respectively) was reported, although no corresponding viability data were provided [74]. In contrast, rutin-Ga(III) complex-mediated aPDI resulted in a > 3 log_10_ reduction in viability, along with a concentration-dependent decrease in *ace* (2-6-fold) and *esp* (4-8-fold) transcript levels [75]. Moreover, gelatinase gene *gelE* exhibited reduced expression (3-6-fold) in cells exposed to photoexcited rutin-Ga(III) complex [75]. Finally, quantitative PCR analysis demonstrated a 10.8-fold downregulation of *fsrB* following C-Phycocyanin-mediated aPDI, with a decrease in survival rate of < 1 log_10_ [76]. Contrary to those findings is the research of Manoil et al., who investigated expression patterns of *ace*, *fsrC*, and *gelE* genes in six strains of *E. faecalis* after treatment with TMPyP and blue light (1–3 log_10_). Data obtained for the *ace* were inconclusive, while *fsrC* and *gelE* displayed strain-dependent regulation upon TMPyP-mediated aPDI [77].

### Motility

The movement of bacteria plays a key role in their virulence, enabling them to colonize tissues, evade the immune system, and penetrate the host barriers [78]. Bacterial motility is mediated by various structures and genetic determinants, depending on the cell envelope architecture. In Gram-negative bacteria, motility is typically associated with flagella driving swimming and swarming or type IV pili mediating twitching motility, whereas Gram-positive bacteria generally lack type IV pili and exhibit motility primarily through flagella, which are structurally and genetically distinct from those of Gram-negative species [79, 80].

Some recent studies have explored the relationship between photodynamic treatments and the expression of flagellin- and pili-related genes. Notably, the available studies examining this phenomenon so far have focused predominantly on Gram-negative bacteria. In the case of aPDI, the reported transcriptional responses are heterogeneous and strongly depend on the utilized photosensitizer. For instance, Boluki et al. reported no significant changes in transcript levels of the *pilZ*, a gene involved in the control and synthesis of type IV pili, in *A. baumannii* cells treated with TBO along with red light. However, no data regarding the antimicrobial efficacy or reduction in bacterial viability were provided [81]. In contrast, in *S. marcescens*, MB-mediated aPDI resulted in a significant decrease in expression of *flhD*, responsible for flagellar motion, by approximately 4- and > 10 fold, depending on the strain, under both sublethal (1–3 log_10_) and lethal (> 3 log_10_) treatment conditions [57]. In *C. sakazakii*, motility-related gene expression was examined in two independent studies employing different photodynamic approaches, revealing opposite responses. In the first study, hypocrellin B-mediated aPDI combined with blue light resulted in a 1–3 log_10_ decrease in viability and led to upregulation of several genes involved in flagellar biogenesis and assembly, including *fliH* (4.5-fold), *fliK* (5.0-fold), *fliC* (3.75-fold), *flgJ* (3.5-fold), and *flgK* (2.5-fold) [82]. In contrast, exposure to aBL at a dose resulting in a < 1 log_10_ reduction in viability led to decreased mRNA level of key motility- and flagellar-associated genes, including *flgJ* (4.0-fold), *motA* and *motB* (4.0-fold), *fliD* (8.0-fold), and *flhD* (4.0-fold) [59]. Blue light illumination was also applied to *Pseudomonas syringae* [83] and *E. coli* [58]. qPCR analysis revealed downregulation of all examined motility-related genes, with the magnitude of transcriptional changes differing markedly between species. In *P.syringae*, expression of the flagellin gene *fliC* was reduced by approximately 2.0-fold (no viability data provided) [83]. Whereas in *E.coli* under conditions causing < 1 log_10_ reduction in viability, reduction of transcript level in *fliA* (≥ 10⁴-fold; near detection limit), *fliC* (2048-fold), *motA* (10⁴-fold), *flhD* (≥ 10⁴-fold; near detection limit), and *flhC* (10⁴-fold) was observed, indicating suppression of the FlhDC-regulated flagellar transcriptional program [58].

### Secretion systems

Secretion of proteins across phospholipid membranes is one of the crucial mechanisms bacteria use to interact with their environment. Released proteins can play roles in promoting virulence by facilitating adhesion, extracting nutrients from the environment niche, or direct toxic effects on target cells [84]. To date, eleven types of secretion systems have been described [85]; however, their classification remains ambiguous. The most extensively studied and characterized types of secretion systems are 1–6 (T1SS-T6SS) in Gram-negative bacteria and type 7 (T7SS) in Gram-positive.

Notably, the available transcriptomic studies addressing secretion systems have primarily focused on the effects of blue light exposure. The RNA-seq analysis of *P. syringae* following blue light illumination revealed that the type 3 secretion system, responsible for the direct injection of effector proteins into host cells, was affected by the treatment, with 18 genes upregulated and 3 genes downregulated in this functional category [83]. For *A. baumannii*, two independent studies regarding secretion systems gene expression under blue light illumination were conducted. In the first study, RNA-seq analysis showed a transcriptional decrease of the *traI* (3-fold), which belongs to the type 4 secretion system involved in protein and DNA transfer, which was subsequently confirmed by RT-qPCR [47]. In the second study, transcriptome analysis indicated that the complete gene cluster of the type 6 secretion system, which functions primarily in interbacterial antagonism, was upregulated by light. RT-qPCR analysis verified these findings by indicating a significant increase in the expression of all representative genes, including *tssM* (anchor protein), *tssD* (secretion protein), and *tssC* (contractile sheath), by 4-16-fold [49].

### Toxin production

Bacterial toxins play a significant role in disease development by facilitating bacterial host colonization, evading the immune system, or causing tissue damage. They can be broadly grouped into two main types: endotoxins - components of the bacterial cell wall, like lipopolysaccharides (LPS), and exotoxins - proteins secreted directly into host cells or the surrounding environment [86].

Several studies have investigated the impact of photodynamic treatments on the expression of toxin-encoding genes, revealing heterogeneous, species-dependent, and photosensitizer-specific responses. In *S. aureus*, aPDI generally led to downregulation of enterotoxin-encoding genes. Ogonowska et al. reported that two distinct treatments, i.e., NMB with red light and RB with green light, both resulting in a < 1 log_10_ decrease in viability, led to downregulation of *seb*, encoding staphylococcal enterotoxin B. Transcript levels were assessed at two time points, revealing 1.15- and 1.87-fold decreases for NMB-aPDI, and 2.05 and 2.82 decreases for RB-aPDI, respectively [24]. More complex transcriptional responses were observed following treatment with gallium porphyrin derivatives. Ga³⁺CHP and Ga³⁺MPIX combined with green light (< 1 log_10_ reduction in viability) significantly downregulated *sec*, encoding staphylococcal enterotoxin C, by 6- and 2.77-fold, respectively. In contrast, a notable upregulation (2.83-fold) of the *tst* gene, encoding toxic shock syndrome toxin-1 (TSST-1), was observed for Ga³⁺CHP-mediated aPDI. This increase in *tst* expression was concomitant with altered transcription of the *srrA* (no significant change) and *srrB* (2-fold) genes, indicating activation of the SrrAB two-component regulatory system, which is known to mediate the bacterial response to oxidative stress [87]. A subsequent study employed the same PS but blue light instead of the previously used green light to investigate two ESKAPE representatives – *S. aureus* and *P. aeruginosa*. RT-qPCR analysis revealed that while Ga³⁺CHP-mediated aPDI (< 1 log_10_) decreased *sec* expression by approximately 13-fold, no significant changes were observed for *tst*. In *P. aeruginosa*, this treatment exerts no impact on the expression of genes responsible for pyocyanin and phenazine production (*phzB2*, *phzM*, and *phzS*) [88]. In contrast, other photodynamic approaches in *P. aeruginosa* revealed opposite transcriptional outcomes. In two independent studies, MB-mediated aPDI resulted in increased expression of toxin-related genes. Specifically, *phzM* was markedly upregulated (1024-fold) under conditions causing a 1–3 log_10_ reduction in viability [41]. While the elastase gene, *lasB*, showed a time-dependent increase of 4.5, 5.1, and 7.9-fold after 1, 2, and 5 min of irradiation, respectively, under conditions associated with < 1 log_10_ (1–2 min) to 1–3 log_10_ (5 min) reductions in viability [25]. Conversely, ALA-mediated aPDI with red light reduced mRNA levels of *phzH* (phenazine pathway) and *lasB* to less than 0.5-fold compared with untreated cells. However, this study reported growth inhibition rather than a reduction in bacterial viability [39]. Finally, in *P. syringae* exposed to blue light, RNA-seq analysis revealed no significant changes in genes related to coronatine (phytotoxin) biosynthesis, whereas RT-qPCR analysis indicated approximately 2-fold downregulation of *cmaA* and *cfa5*, involved in the synthesis of coronamic acid and coronafacic acid, respectively [83].

Taken together, the available studies show that both aPDI and aBL can modulate the expression of a broad range of virulence-associated genes. However, these effects are highly species-dependent and strongly influenced by the applied photosensitizer, light parameters, and experimental conditions. Importantly, transcriptional changes have been observed both under conditions associated with bacterial killing and in the absence of measurable antimicrobial effects, indicating that modulation of gene expression does not consistently correlate with treatment efficacy. In several studies, the extent of transcriptional regulation appeared to depend more on photosensitizer concentration than on bactericidal outcome. These observations highlight the complexity of photodynamic responses and underline the need to interpret gene expression data in the context of both treatment parameters and phenotypic changes.

**Fig. 1:**
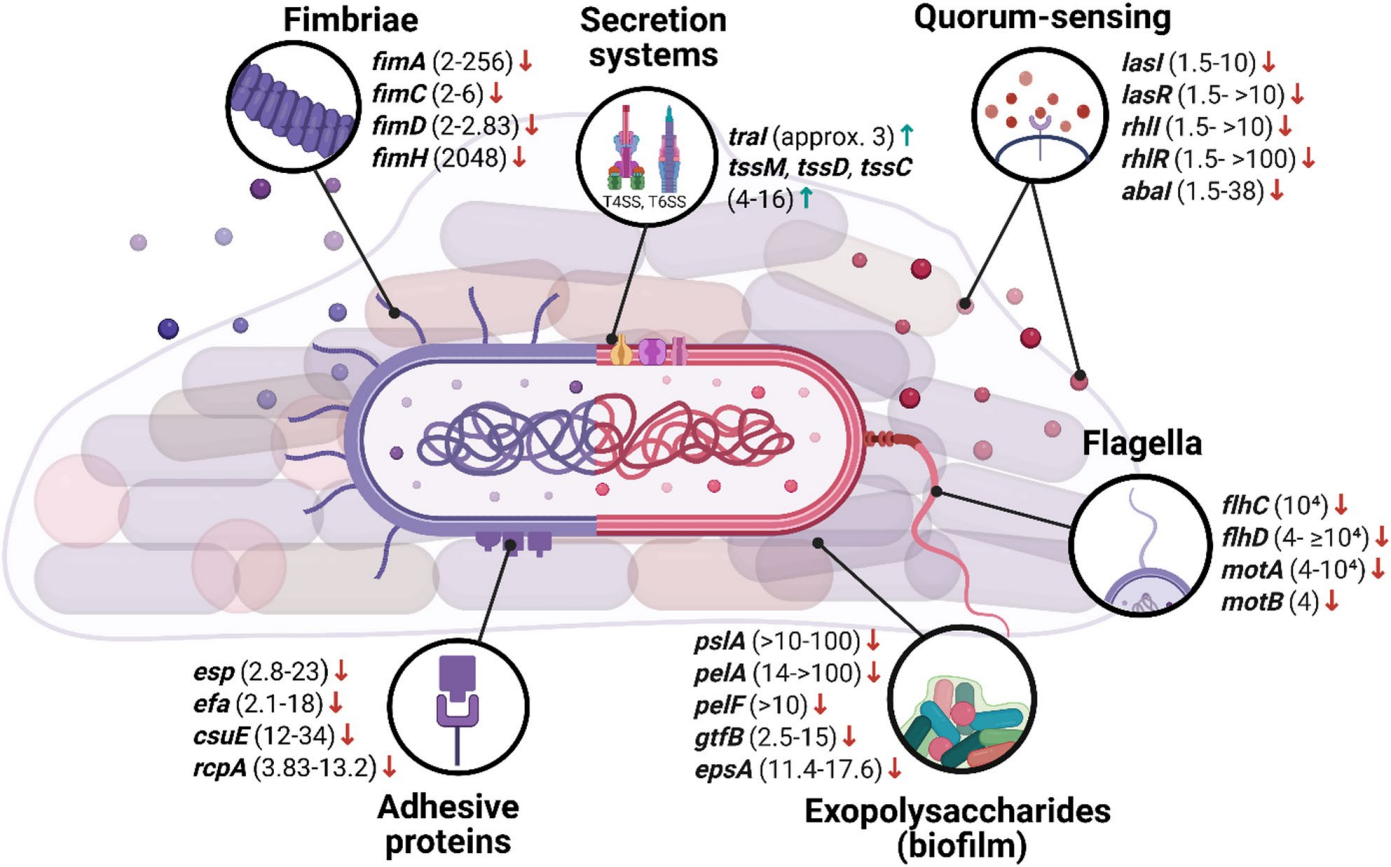
Graphical representation of selected genes altered after aBL/aPDI treatment regarding virulence factors. Arrows indicate direction of change, i.e., “↓” represents downregulation of a particular gene, while “↑” represents upregulation. Fold-change values shown in the figure represent the range between the lowest and highest reported magnitude of transcriptional change across the available studies for a given gene, unless only a single study was available. The genes presented in the figure were examined across multiple bacterial species. Adhesion- and fimbriae-related genes included *fimA*, investigated in *P. gingivalis*, *S. marcescens*, *E. coli*, and *A. baumannii*; *fimC* in *A. baumannii* and *S. marcescens*; *fimD* in *A. baumannii*; and *fimH* in *E. coli*. Genes related to secretion systems comprised *traI* and *tssC*, *tssD*, and *tssM*, all analyzed in *A. baumannii*. Quorum-sensing-associated genes *lasI*, *lasR*, *rhlI*, and *rhlR* were investigated in *P. aeruginosa*, while *abaI* was examined in *A. baumannii*. Biofilm- and adhesion-associated genes included *esp* and *efa* in *E. faecalis*, *csuE* and *epsA* in *A. baumannii*, *rcpA* in *A. actinomycetemcomitans*, *pslA*, *pelA*, and *pelF* in *P. aeruginosa*, and *gtfB* in *S. mutans*. Motility-related genes comprised *flhC* in *E. coli*; *flhD* in *S. marcescens*, *E. coli*, and *C. sakazakii*; *motA* in *E. coli* and *C. sakazakii*; and *motB* in *C. sakazakii*

The full list of differentially expressed genes regarding virulence factors is provided in Supplementary materials (Table S1.) and selected representative genes are presented in Fig. 1.

## Mechanisms of DNA repair

DNA strands are one of the main targets of photoinactivation [89]. Phototreatment can cause DNA oxidations and strand breaks, which may lead to cross-links between proteins and DNA or cross-links between DNA strands and eventually mutations [90, 91]. As a result, cell division can be arrested. All changes caused by photoinactivation can be lethal for bacteria. The level of accumulated DNA damage and cell survival rate depend on the species, the light dose, and the PSs used [91]. In research investigating gene expression after phototreatment, applied doses of light do not cause the eradication of all bacterial load, but noticeable changes in the gene expression profile compared to the non-irradiated control. These practice is used to catch what happens in bacterial cells before eradication and discover how bacteria defend themselves. It is also important for potential applications when the lethal dose of light cannot eventually be delivered to the entire bacterial population on the lesional skin or in the infected wound [24]. This dose of light, which does not cause complete inactivation, can be a signal to engage needed DNA repair systems, but not all of them immediately, as can be observed in the gene expression profile of bacteria depending on the dose of phototreatment and the duration of overall light exposure [23]. Most research investigating gene expression following aPDI/aBL has focused on the SOS response system, a global bacterial response to DNA damage. This system involves the sequential and differential expression of approximately 40 genes, including *recA*,* recX*,* recN*,* umuC*,* umuD*, and others [92]. In brief, after the accumulation of ssDNA and strand breaks, which block DNA polymerase, the RecA protein builds filaments with DinI around damaged DNA. Then, RecA deactivates LexA repressor, leading to SOS system gene expression, and DinD is required for disassembly of the RecA polymer after successful DNA repair [93].

Upregulation of the SOS response gene *recA* has been reported in several bacterial species following light-based treatments, with a relatively consistent increase in transcript levels across different experimental models. Increased *recA* expression was observed in *Streptococcus agalactiae* treated with RB activated by green light (approx. 3-fold, < 1 log_10_ reduction in bacterial load) [94], *E. coli* exposed to a blue light laser (2.64-fold, < 1 log_10_ reduction) [95], and *S. aureus* subjected to either blue light or RB with green light (approx. 2-fold for both treatments, 1–3 log_10_ reduction) [96]. In *S. mutans* biofilm, *recA* transcript levels increased by approximately 2-fold following TBO-mediated aPDI and by 3-fold after NMB treatment, both under conditions causing > 3 log_10_ reduction in viability [97]. In contrast, no significant changes in *recA* expression were reported for *A. baumannii* treated with TBO, where only growth inhibition rather than bacterial killing was observed [45] nor for *E. coli* treated with PVP-curcumin and blue light (1–3 log_10_ reduction at 10 µg/mL; no statistically significant reduction at 50 µg/mL) [98]. This observation is consistent with previous reports indicating that curcumin can inhibit the bacterial SOS response [99]. Similarly, no *recA* upregulation was detected in *E. coli* irradiated with leneYR (< 1 log_10_ reduction), but an increased amount of RecA was detected by Western Blot, and RNA-seq data showed increased expression of *recX* - the *recA* inhibitor [100]. The upregulation of other SOS response genes was observed in *E. coli* treated with a blue laser (< 1 log_10_ reduction), including *recN* (2.5-fold), *yebG* (2.1-fold), *dinI* (2.3-fold), and *dinD* (2-fold). Interestingly, the upregulation of the SOS response relates to the coexpression of zinc and copper metabolic pathway genes connected with the SOS response process: *zraP* (2.93-fold) *and copA* (3.73-fold) [95].

The pattern of gene expression in DNA repair systems also varies between bacterial species and depends on light dose and exposure time. In *Campylobacter jejuni*, blue light treatment was analyzed at two time points (T1: 15 min; T2: 30 min), allowing assessment of time-dependent transcriptional responses in DNA repair pathways. Under these conditions, multiple genes were downregulated, including Holliday junction helicase *ruvB* (T1: 1.5-fold; T2: 6-fold), crossover junction endodeoxyribonuclease *ruvC* (T1: 1.97-fold; T2: 4.26-fold), repair protein *recN* (T1: 1.31-fold), base excision repair *exoA* (T1: 3.41-fold; T2: 9.67-fold), single-stranded DNA-binding protein *ssb* (T1: 4.71-fold; T2: 4.27-fold), DNA gyrase subunit A and B *gyrA* (T1: 2.38-fold; T2: 4.78-fold) and *gyrB* (T1: 1.5-fold; T2: 2.07-fold), DNA ligase *ligA* (T1: 2.62-fold; T2: 3.55-fold), and DNA polymerase III subunit beta and gamma *dnaN* (T1: 1.38-fold; T2: 1.93-fold) and *dnaX* (T1: 2.38-fold; T2: 2.93-fold). In contrast, moderate upregulation was observed for Holliday junction helicase *ruvA* (T1: 1.44-fold; T2: 1.63-fold), DNA polymerase III subunit alpha *dnaE* (T1: 1.69-fold; T2: 1.66-fold), repair protein *recA* (T1: 1.51-fold; T2: 1.57-fold), single-stranded-DNA-specific exonuclease *recJ* (T1: 1.35-fold; T2: 1.39-fold), and *pgi* (T2: 1.43-fold), encoding phosphoglucose isomerase, which also participates in the DNA repair processes. Notably, no statistically significant reduction in bacterial load was reported at T1, whereas T2 was associated with a 1–3 log_10_ decrease in viability [22]. Similar to the time-dependent effects observed in *C. jejuni*, light-dependent transcriptional responses were also reported in *E. coli*. Following blue light irradiation at different wavelengths, upregulation of several genes associated with stress response and DNA repair was observed. Specifically, increased transcript levels were reported for adenylosuccinate synthetase *purA* (1.98-fold at 415 nm; 3.1-fold at 409 nm), 30 S ribosome binding factor *rbfA* (3.47-fold at 415 nm; 2.68-fold at 409 nm), and the error-prone DNA polymerase V subunit *umuD* (4-fold at 415 nm; 3.36-fold at 409 nm), while no significant changes were detected for phosphopentomutase *deoB*. These transcriptional changes occurred under conditions associated with a < 1 log_10_ reduction in bacterial viability [23]. In *Vibrio cholerae*, blue light irradiation induced strong upregulation of the *phr* gene (ΔExpression value: 822), responsible for DNA repair responses to UV-induced damage; however, no data on bacterial viability were provided [101]. In *S. aureus*, increased expression of the error-prone DNA polymerase V subunit *umuC* (approx. 2-fold) was observed following both aBL and RB-mediated aPDI with green light [96]. In contrast, *E. coli* subjected to PVP-curcumin mediated aPDI exhibited no change in the expression level of this gene, despite treatment conditions resulting in a 1–3 log_10_ reduction in viability at 10 µg/mL, or no statistically significant reduction at the higher concentration of 50 µg/mL [98].

As bacterial DNA is one of the primary targets of light-based antimicrobials, it is also crucial to discuss its impact on host eukaryotic DNA, the safety of aPDT, and potential genotoxicity. Investigations for methylene blue and visible light on keratinocytes show no immediate and delayed genotoxic damage up to 180 min [102]. Other analyses revealed that RB-mediated aPDI with the photosensitizer at lower therapeutic concentrations (0.2 µM RB and 0.2 µM RB aPDI) does not cause any DNA damage in the eukaryotic epithelial cell line VK2E6E7. Employment of higher doses (5 and 30 µM RB aPDI) caused a mild mutagenic effect [103]. Specific tests for other cell lines and light-photosensitizer combinations are still required. To conclude, it is possible to establish light and photosensitizer doses that are both bactericidal and safe for host cells, simultaneously, called a “safe therapeutic window” [104].

In summary, for DNA protection, bacteria employed multiple DNA repair and SOS stress response strategies, depending on the species and the level of damage induced by the applied light-based method. Simultaneously, DNA replication is arrested by downregulating genes encoding components of this process.

**Fig. 2:**
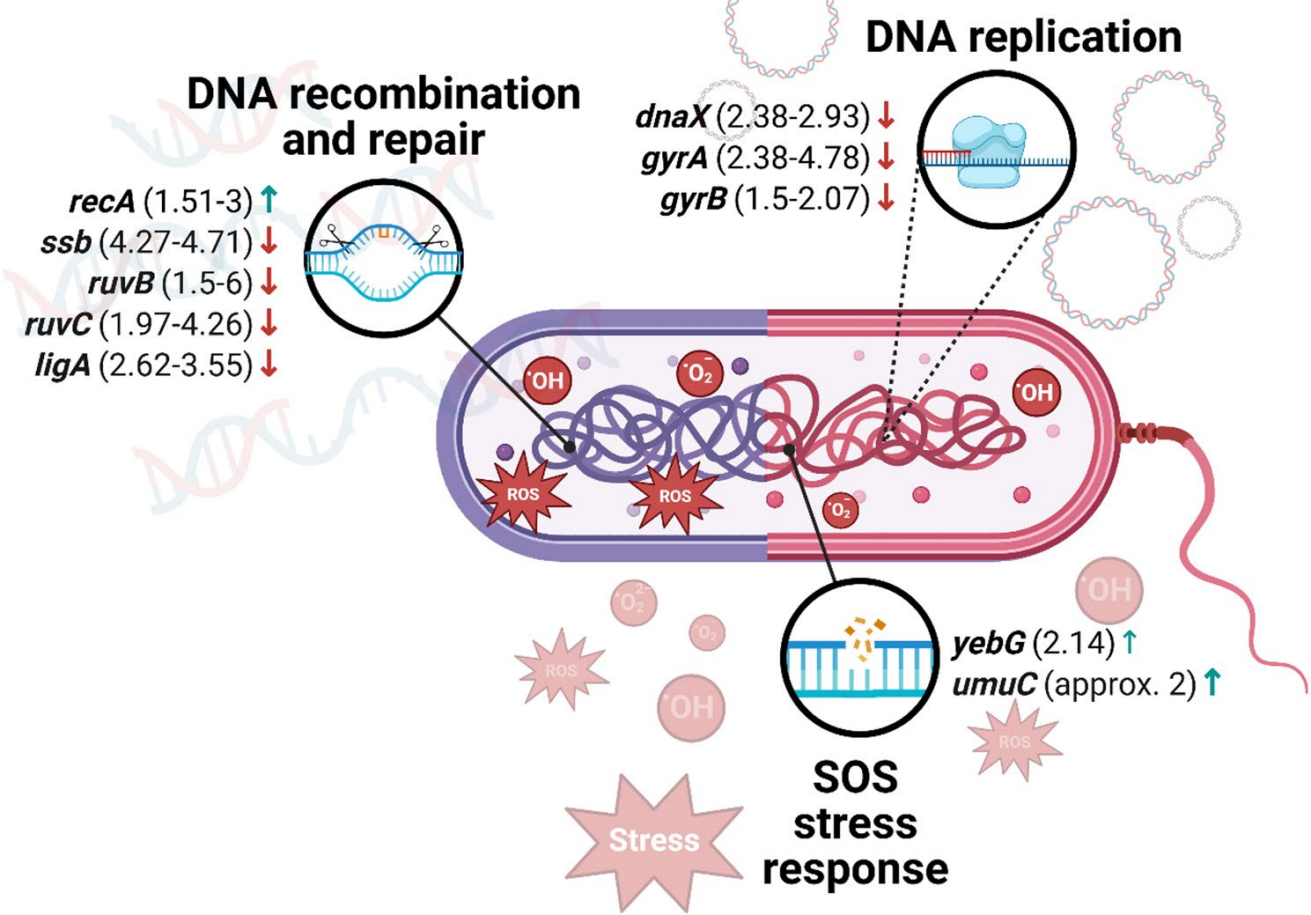
Graphical representation of selected genes altered after aBL/aPDI treatment regarding mechanisms of DNA repair. Arrows indicate direction of change, i.e., “↓” represents downregulation of a particular gene, while “↑” represents upregulation. Fold-change values shown in the figure represent the range between the lowest and highest reported magnitude of transcriptional change across the available studies for a given gene, unless only a single study was available. The SOS response gene *recA* was investigated in *S. agalactiae*, *A. baumannii*, *S. mutans*, *E. coli*, *S. aureus*, and *C. jejuni*. Genes associated with homologous recombination and DNA repair, including *ssb*, *ruvB*, *ruvC*, *ligA*, *dnaX*, *gyrA*, and *gyrB*, were examined in *C. jejuni*. Stress-related gene *yebG* was analyzed in *E. coli*, while the error-prone DNA polymerase gene *umuC* was investigated in *E. coli* and *S. aureus*

The full list of differentially expressed genes regarding DNA repair is provided in Supplementary materials (Table S2.) and selected representative genes are presented in Fig. 2.

## Detoxification and stress response

This paragraph aims to explore the molecular mechanisms underlying bacterial responses to oxidative stress and detoxification processes induced by aPDI and aBL. It is well known that microorganisms and higher organisms possess a wide range of tools enabling defense against ROS that can cause protein oxidation, lipid peroxidation, and DNA damage [105]. It should be noted that the studies summarized in this section primarily describe acute transcriptional responses to light-based treatments, typically observed in strains that remain susceptible to photoinactivation and respond by activating oxidative stress and detoxification pathways.

In response to oxidative stress, specific regulons are activated. In Gram-negative bacteria, the transcription factor OxyR is typically responsible for sensing hydrogen peroxide (H₂O₂), whereas in many Gram-positive species, this role is often fulfilled by PerR [106]. To confer protection against the superoxide radicals, the SoxRS system is involved; however, it also influences the expression of the SoxS, which encodes, for example, superoxide dismutase, oxidation-resistant biosynthetic enzymes, the DNA repair nuclease, or xenobiotic efflux pumps [107]. By examining the transcriptomic responses of various bacterial species to phototreatments and therefore investigating the detoxification “arsenal”, we aim to provide a deeper understanding of how bacteria adapt to light-induced oxidative stress and identify potential targets for improving the efficacy of aPDI and aBL in microbial control.

Buchovec et al. investigated the molecular effects of chlorophyllin (Chl)-based aPDI, which resulted in a 1–3 log_10_ reduction in viability, on *Salmonella enterica* serovar Typhimurium by analyzing the expression of 33 stress-related genes. The treatment induced oxidative stress, as evidenced by upregulation of key genes involved in ROS detoxification, such as *oxyR* (1.6-fold), *ahpC* (2.75-fold), and *grxA* (1.8-fold). Additionally, increased expression of *sulA* (1.8-fold) and *atpC* (2.75-fold) indicated disruption of cell division and inhibition of oxidative respiration. Another upregulated gene, *STM0225* (1.75-fold), was also associated with stress response and cellular adaptation [108]. Kim and Yuk investigated the impact of light illumination that did not result in a statistically significant reduction in bacterial viability, on gene expression levels in *Salmonella enterica* serovar Enteritidis (sensitive to aBL) and *Salmonella enterica* serovar Saintpaul (resistant to aBL). Their findings revealed that while only *oxyR* was upregulated in both non-illuminated and illuminated *S. Enteritidis* cells, all tested genes (*oxyR*, *recA*, *rpoS*, *sodA*, and *soxR*) were upregulated in *S. Saintpaul* under the same conditions [18].

In a subsequent study, Tardu et al. investigated the role of ChrR and SigmaE in regulating gene expression in *V. cholerae* in response to blue light irradiation. Their study showed that the genome-wide response to light is mediated by the ChrR anti-sigma factor and its cognate partner, SigmaE (σE). A highly upregulated putative ChrR operon consisting of ChrR (VC2301), SigmaE (VC2302), and a hypothetical gene (VC2303) was identified; however, quantitative data specifying exact fold-change values were not provided. In their experiments, a ΔChrR *V. cholerae* mutant was generated, and RNA-seq analysis revealed that 159 differentially expressed genes (DEGs) in wild-type cells were no longer regulated in the ΔChrR mutant, while 63 of the DEGs remained regulated. Furthermore, the researchers found that ChrR and σE work together to regulate gene expression in response to blue light. Additionally, the study revealed a consensus σE-binding sequence (TGATC-N18-CGTAT) upstream of 31 operons and 29 genes, highlighting the key role of σE in the photo-oxidative stress response. A KEGG pathway enrichment analysis showed that genes associated with pathways like geraniol degradation, fatty acid degradation, and propanoate metabolism were still significantly regulated in the ΔChrR mutant in response to blue light, further confirming the involvement of ChrR and σE in the light-regulated gene expression [101].

Similarly, Muller et al. analyzed metabolic and virulence-associated transcriptional changes in *A. baumannii* upon exposure to 462 nm blue light, using RNA-seq analysis. The authors observed increased expression of the gene encoding catalase – *katE* by 7.1-fold in comparison to the untreated cells. It is worth noting that RT-qPCR analysis confirmed the increased expression of *katE* in response to blue light, supporting previous findings [49]. Following this, Pourhajibagher et al. investigated the impact of photo-activated disinfection on the expression of *oxyR* in *P. gingivalis*. The study included 16 clinical isolates of *P. gingivalis* that were photosensitized with toluidine TBO, MB, and ICG, followed by irradiation with a diode laser at a specific wavelength for each PS. All applied aPDI conditions were characterized by no statistically significant reduction in bacterial viability. RT-qPCR analysis revealed that MB-, TBO-, and ICG-mediated aPDI significantly increased *oxyR* expression compared to the untreated control group. The expression levels were elevated approximately 5.6-, 8.5-, and 12.3-fold following MB-, TBO-, and ICG-aPDI treatment, respectively. Of note, the expression level in the ICG-aPDI group was significantly higher than in the MB-aPDI group, whereas no significant difference was observed between ICG-aPDI and TBO-aPDI. Additionally, under H₂O₂ treatment as a positive control, *oxyR* expression increased by approximately 12.8-fold. The expression levels in the H₂O₂ and ICG-aPDI groups were significantly higher than in the TBO- and MB-aPDI groups, but no remarkable difference was observed between H₂O₂ and ICG-aPDI [109]. These differences indicate that transcriptional responses to photodynamic approaches are strongly dependent on the type of PS used. Variability in *oxyR* induction may reflect differences in photophysical properties, cellular uptake, subcellular localization, and ROS generation profiles of individual PS. In particular, ICG, due to its near-infrared absorption and photothermal characteristics, may generate a distinct oxidative stress signature compared to phenothiazinium dyes such as MB or TBO. Consequently, gene expression patterns observed following aPDI should be interpreted in a photosensitizer-specific context rather than as a uniform response to light exposure.

Continuing the investigation into oxidative stress responses, Bustamante et al. examined the transcriptional response of *Klebsiella pneumoniae* aPDI (> 3 log_10_ reduction in viability) with [Ir(ppy)2(ppdh)]PF6 (PSIR-3), focusing on oxidative stress-related gene expression. The results indicated that the response occurs primarily via the extracytoplasmic sigma factor *rpoE* and the RNA-binding protein *hfq*, whereas no involvement of the *oxyR* pathway or *sodA* gene induction was observed. The release of *rpoE* triggered the activation of the extracytoplasmic regulon, leading to a strong upregulation (> 6-fold) of the *rpoE* in both *K. pneumoniae* strains upon PSIR-3-mediated aPDI, whereas treatment with the ([Ru(bpy)3](PF6)2 (PS-Ru) reference compound resulted in only approx. 2.5-fold increase. This suggests that *rpoE* plays a crucial role in responding to photooxidative stress induced by PSIR-3. Similarly, *hfq*, a gene involved in post-transcriptional regulation, was significantly upregulated (> 6-fold) in both *K. pneumoniae* strains after PSIR-3 treatment, indicating the participation of small RNAs in the photooxidative stress response via the Type II mechanism [110]. In a subsequent study, Manoil et al. investigated the transcriptional responses of *E. faecalis* strains to TMPyP-mediated aPDI (1–3 log_10_ decrease in viability), focusing, among others, on oxidative stress and general stress responses. Regarding oxidative stress response genes, *dps* (DNA-binding protein) was significantly upregulated (> 2-fold) in almost all strains, primarily after exposure to TMPyP alone. Meanwhile, *hypR* (transcriptional activator) was upregulated in the reference strain following exposure to TMPyP or TMPyP combined with light (4-fold). For general stress response genes, *dnaK* (chaperone-encoding) showed notable upregulation, reaching a 30.1-fold increase in *E. faecalis* reference strain after TMPyP-aPDI. A *σV* (extra-cytoplasmic function sigma factor) was highly upregulated in all strains except the reference one, with a peak increase of 38.7-fold after blue light exposure. *relA* (stringent response) exhibited both up- and downregulation across strains but was not specifically induced by TMPyP-aPDI [77].

Walker et al. performed RNA-seq analysis that indicated a two-stage response to aBL in *C. jejuni*. The initial phase, observed under bacteriostatic conditions (T1), was characterized by upregulation of chaperone and oxidative stress resistance genes, including *grpE* (34.2-fold), *groES* (9.65-fold), *groEL* (3.60-fold), *clpB* (9.31-fold), *dnaK* (8.44-fold), *hrcA* (38.68-fold), and *hspR* (3.09-fold). At bactericidal dose (T2; 1–3 log_10_ reduction in viability), the response was stronger, reaching 47.56-, 67.68-, 4.68-, 20-, 8.49-, 159.63-, and 7.32-fold for each gene, respectively. Moreover, *katA* (catalase, 111.82-fold), *ahpC* (thiol-peroxidase, 5.22-fold), *trxB* (thioredoxin reductase, 3.96-fold), and other stress-related genes were highly expressed. Notably, genes encoding key components of the electron transport chain (*cydA*, *cydB*, and *ccoN*) were downregulated by 4.11-, 3.20-, and 2.4-fold at T2. Transcriptomic data revealed that 352 genes were differentially expressed at the bacteriostatic dose, while 756 genes exhibited altered transcription at the bactericidal dose, with 58 and 275 genes, respectively, showing a ≥ 4-fold change. Several genes associated with oxidative stress defense and protein quality control were found to be upregulated in *C. jejuni*; among these, *cj0379c* (*msrP*; 6.6-fold at T2), encoding methionine sulfoxide reductase, and *cj0358* (2.86-fold at T1; 1.85-fold at T2, encoding cytochrome c peroxidase, likely contribute directly to the detoxification of reactive oxygen species. Additionally, the upregulation (6.84-fold at T2) of *csrA*, a global post-transcriptional regulator, suggests broader modulation of stress-related translation pathways. Of particular interest is the induction of the disulfide bond formation system, including *dsbA2* (1.79-fold at T2), *dsbB* (2.36-fold at T1; 3.45-fold at T2), and *dsbD* (1.57-fold at T1; 4.88-fold at T2), which are involved in proper protein folding and stabilization through disulfide bridge formation. This system may indirectly support oxidative stress defense by ensuring the structural integrity of periplasmic and membrane proteins, especially those with antioxidant or stress-related functions. These findings highlight the oxidative stress-induced damage caused by aBL and the adaptive responses that contribute to *C. jejuni* survival [22]. This aspect is further explored in the following section, where the role of protein damage and the heat shock response is investigated.

In a separate study, He et al. investigated the dose-dependent effects of ALA-mediated aPDI on the expression of four oxidative stress-related genes (*sodA*, *PPA0097*, *ahpC*, and *oxyR*) in *Cutibacterium acnes*. Low ALA concentrations (0.05–0.5 mmol/L) did not result in a measurable decrease in bacterial viability, whereas higher concentrations (1.0–2.5 mmol/L) were associated with a < 1 log₁₀ reduction in viability. Low-dose ALA-aPDI may stimulate bacterial proliferation by inducing mild oxidative stress and triggering ROS-mediated signaling, whereas high-dose aPDI leads to bacterial death due to excessive oxidative damage. Low-dose ALA-aPDI induced an adaptive oxidative stress response, with increased expression of *sodA* and *PPA0097*, promoting bacterial survival. In contrast, higher ALA-aPDI doses resulted in a decline in antioxidant enzyme activity, likely due to excessive ROS accumulation causing oxidative damage. *sodA* (2.0-fold at 0.5 mmol/L, 5.0-fold at 1.0 mmol/L, and 6.0-fold at 2.5 mmol/L) and *PPA0097* catalase (1.5-fold at 0.1 mmol/L, 2.5-fold at 0.5 mmol/L, 3.75-fold at 1.0 mmol/L, and 7.0-fold at 2.5 mmol/L) exhibited a linear correlation with ALA dosage. In contrast, *oxyR* (1.2-fold at 0.05 mmol/L, 0.5-fold at 0.1 mmol/L, 2-fold at 0.5 mmol/L, and 3.5-fold at 2.5 mmol/L) and *ahpC* (3-fold at 0.5 mmol/L, 2-fold at 1 mmol/L, and 3-fold at 2.5 mmol/L) showed a non-linear correlation with ALA concentrations [111].

While these transcriptional responses reflect important defense mechanisms against light-induced oxidative stress, they do not necessarily translate into increased survival or tolerance, which may instead require distinct regulatory patterns associated with repeated or sublethal photodynamic exposure, as discussed in Potential mechanisms involved in light tolerance.

**Fig. 3:**
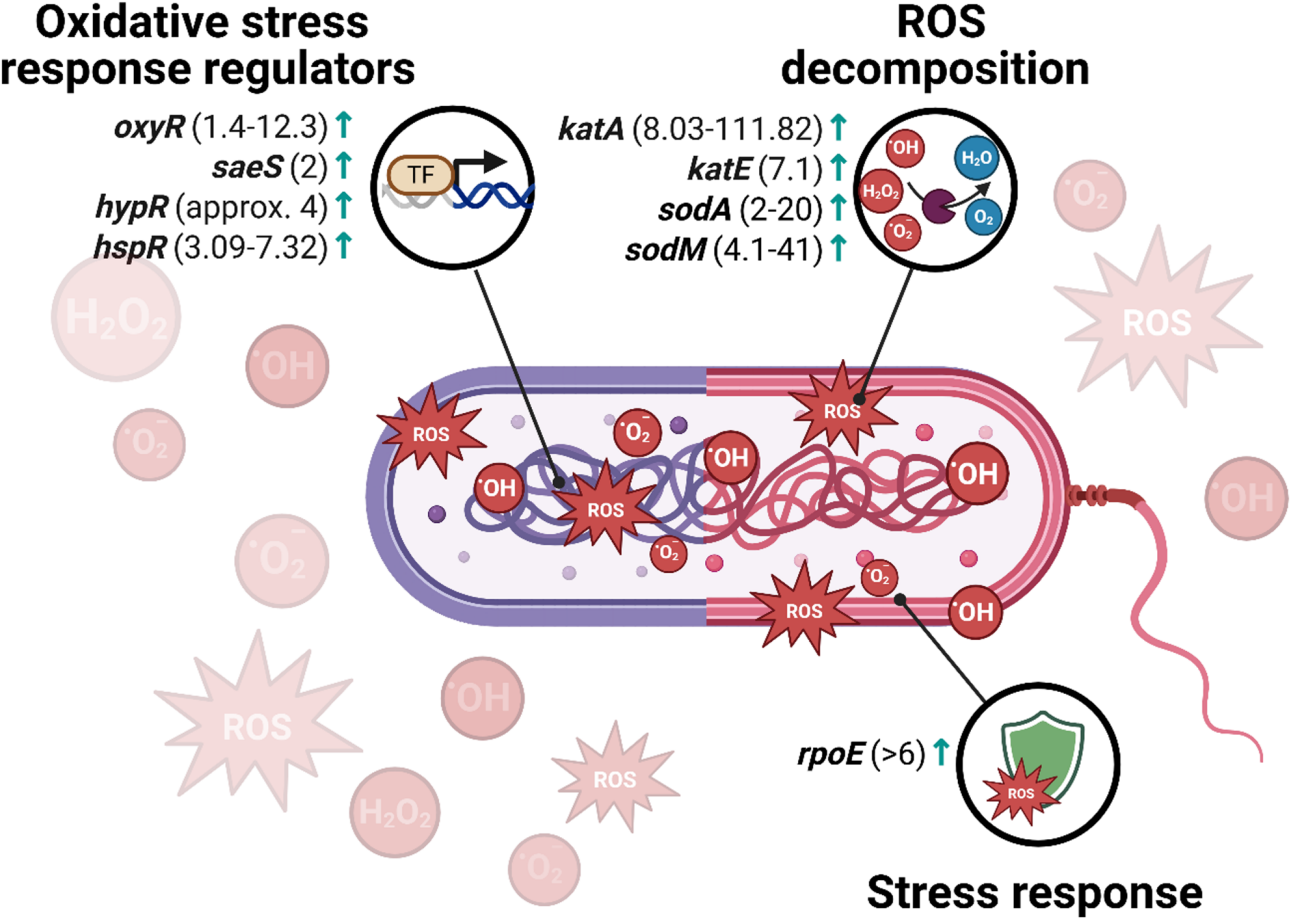
Graphical representation of selected genes altered after aBL/aPDI treatment regarding detoxification and stress response. Arrows indicate direction of change, i.e., “↓” represents downregulation of a particular gene, while “↑” represents upregulation. Fold-change values shown in the figure represent the range between the lowest and highest reported magnitude of transcriptional change across the available studies for a given gene, unless only a single study was available. The oxidative stress regulator *oxyR* was investigated in *C. acnes*, *K. pneumoniae*, *P. gingivalis*, *S. enterica*, *S. Enteritidis*, and *S. Saintpaul*. The superoxide dismutase gene *sodA* was analyzed in *C. acnes*, *K. pneumoniae*, *S. Enteritidis*, *S. Saintpaul*, and *S. aureus*, whereas *sodM* was examined exclusively in *S. aureus*. The two-component regulatory gene *saeS* was analyzed in *S. aureus*, while *hypR* was examined in *E. faecalis*. Heat shock regulator *hspR* and catalase-encoding genes *katA* and *katE* were investigated in *C. jejuni*. The extracytoplasmic stress sigma factor *rpoE* was analyzed in *K. pneumoniae*. ROS – reactive oxygen species

The full list of differentially expressed genes regarding detoxification and stress response is provided in Supplementary materials (Table S3.) and selected representative genes are presented in Fig. 3.

## Defense mechanisms

### Heat shock proteins

Heat-shock proteins (HSPs) allow a broad spectrum of organisms, including bacteria, to cope with multiple unfavorable factors (oxidative stress, antibiotic presence, immediate increase in temperature, etc.). HSPs play a chaperone activity by taking part in appropriate protein folding, preventing the creation of aggregates, and degradation of irreparable peptides [112, 113]. Expression of the genes encoding HSPs is important in terms of the health of the cell and the longevity of an organism [114]. The greatest importance in prokaryotic organisms is linked with the presence of chaperone families such as HSP60 (GroEL, GroES in *E. coli*), HSP70 system proteins (DnaK, DnaJ, and GrpE in *E. coli*), HSP90 chaperones, and HSP100 (ClpA and ClpB in *E. coli*) [114, 115]. The increased expression of their genes encoding HSPs under oxidative stress is a signal of tolerance and overcoming the harsh conditions of oxidative stress, a phenomenon that has been studied many times by many research groups.

In the study conducted by Yang et al., RNA-seq analysis of the MRSA strain upon aBL (< 1 log_10_ reduction in viability) revealed mainly an upregulation of phage-related genes and downregulation of only one of the genes encoding the cooper chaperone (SAR 2639). However, quantitative data specifying exact fold-change values were not provided [116]. Similarly, in the study by Adair et al., the small heat shock protein gene, *narK*, was downregulated (9.24-fold) in *S. aureus* after exposure to blue light (no data on survival after treatment) [117]. Furthermore, Pourhajibagher et al. showed that exposure of *A. baumannii* clinical and reference strains to red light and TBO resulted in an increased expression of chaperonin encoded by the *dnaK* by 5.6 and 4.4-fold, respectively, with no effect on survival rate [45]. Nevertheless, a study conducted by Walker et al. presented RNA-seq analysis of *C. jejuni* exposed to: a lower (bacteriostatic – T1) aBL dose (with no bactericidal effect) and a higher aBL dose (bactericidal – T2), resulting in a reduction in growth by 1–3 log_10_. Upon exposure to T1 aBL dose, HSP70 cofactor gene (*grpE*), co-chaperonins *(groES*), or co-chaperonins such as *groEL* were upregulated (34.2-fold), (9.65-fold), and (3.60-fold), respectively. Additionally, *clpB* (9.31-fold) and *dnaK* (8.44-fold) were overexpressed upon low aBL dose, whereas upon higher aBL presented even higher expression levels: *groES* (67.68-fold), *clpB* (20.04-fold). Furthermore, the heat-inducible transcription repressor *hrcA* and, to a lesser extent, heat shock transcriptional regulator *hspR*, were also upregulated following exposure to the lower aBL dose – 38.68-fold and 3.1-fold, respectively. Even higher upregulation of the following genes was observed upon exposure to a bactericidal dose of aBL: *hrcA* (159.63-fold) and *hspR* (7.32-fold) [22]. Notably important research was conducted by Muehler et al., who performed transcriptomic analysis of *E. coli* upon exposure to SAPYR photosensitizer and 380–600 nm light (reduction below 1 log_10_). RNA-seq analysis underlined the increased expression of the periplasmic chaperonin encoded by *cpxP* (97-fold), and upregulation of chaperones encoded by genes: *ibpA* (38.6-fold), *ipbB* (98-fold), *clpB* (13.3-fold), *hslO* (15.4-fold), *cspI* (51.7-fold), and *spy* (119-fold) [100]. On the other hand, authors observed a decrease in the expression of periplasmic chaperones encoded by the genes: *hdeA* (13.9-fold), *hdeB* (16-fold), and *hdeD* (21.1-fold), and for genes encoding decarboxylases, which take part in acid stress expression due to the participation in the gad system activated during the occurrence of extremely acidic conditions: *gadA* (27.9-fold), *gadB* (36.8-fold), and *gadC* (27.9-fold). Authors indicate that the presence of PS in the environment and its binding to the bacterial membrane, as well as during the phototreatment, results in the pH changes that have a reflection in the decrease of expression of periplasmic chaperones [100].

**Fig. 4:**
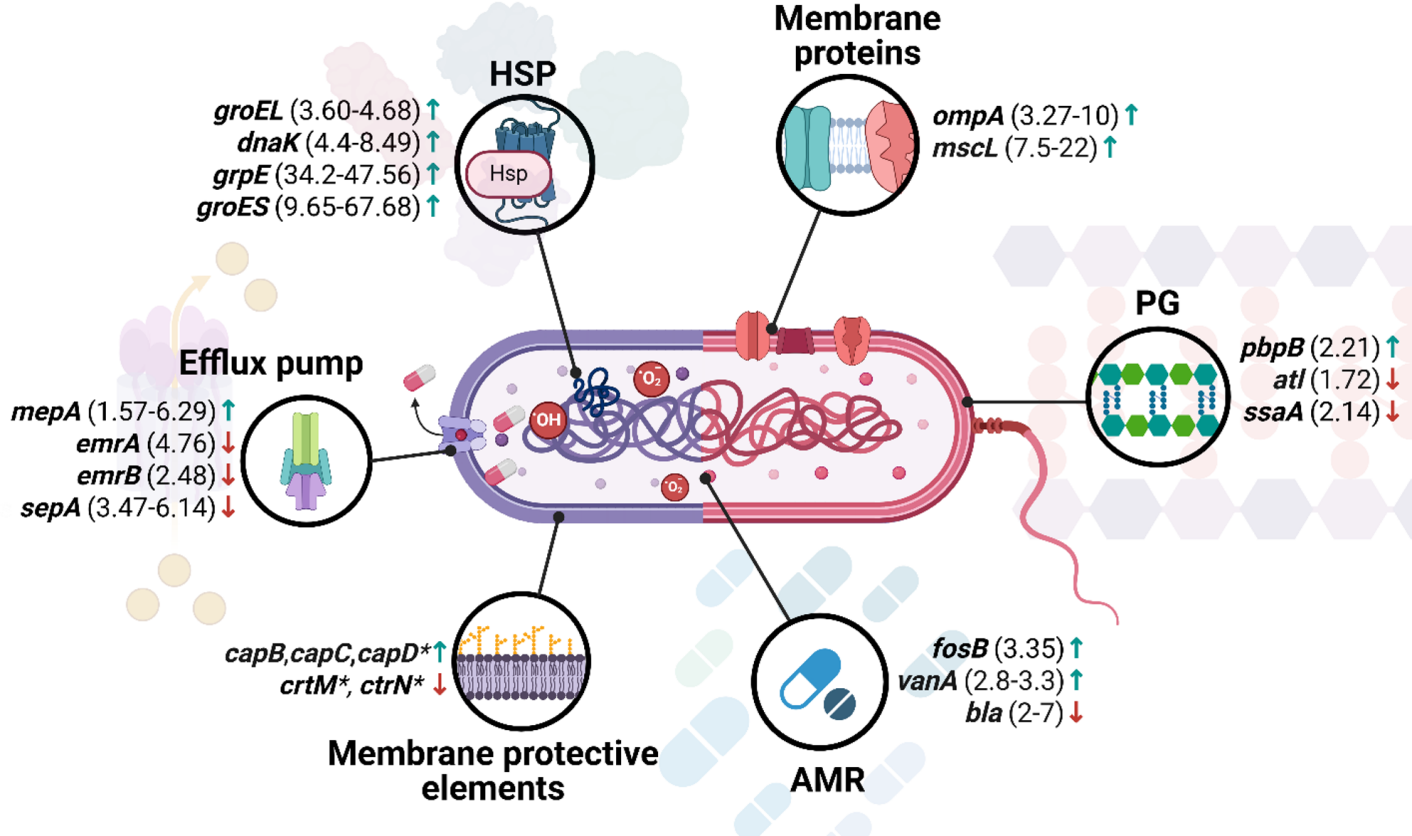
Graphical representation of selected genes altered after aBL/aPDI treatment regarding defense mechanisms. Arrows indicate direction of change, i.e., “↓” represents downregulation of a particular gene, while “↑” represents upregulation. Fold-change values shown in the figure represent the range between the lowest and highest reported magnitude of transcriptional change across the available studies for a given gene, unless only a single study was available. Heat shock proteins and chaperone-encoding genes, including *groEL*, *groES*, *grpE*, and *dnaK*, were predominantly investigated in *C. jejuni*, while *dnaK* was additionally examined in *A. baumannii*. Multidrug efflux pump components *emrA* and *emrB* were analyzed in *A. baumannii*, whereas *mepA* and *sepA* were investigated in *S. aureus*. Genes involved in capsule biosynthesis and cell envelope-associated virulence, including *capB*, *capC*, *capD*, *crtM*, and *crtN*, were examined in *S. aureus*. Outer membrane protein *ompA* was investigated in *A. baumannii*. The mechanosensitive channel gene *mscL* was analyzed in *E. coli*. Antibiotic resistance–associated genes included *pbpB*, *atl*, *ssaA*, and *fosB* in *S. aureus*, *bla* in *E. coli*, and *vanA* in *E. faecalis*. HSP – heat shock proteins; PG – peptidoglycan; AMR – antimicrobial resistance; *Authors did not provide fold change values

The full list of differentially expressed genes regarding heat shock proteins is provided in Supplementary materials (Table S4.) and selected representative genes are presented in Fig. 4.

### Antimicrobial resistance

Many studies demonstrate the efficacy of aPDI (e.g., RB, TBO, Gallium porphyrins) or aBL as a tool for phenotypic re-sensitization of multidrug-resistant pathogens to antimicrobials [8, 88, 118–121]. Notably, several studies suggest that the primary mechanism behind the bactericidal activity of antibiotics is oxidative stress caused by the production of ROS, which arises as a consequence of interactions between antibiotics and their conventional molecular targets [122, 123]. However, there is a limited number of studies indicating changes in the antibiotic resistance genes at the transcriptomic level, especially in the context of expression patterns upon oxidative stress via aPDI/aBL activity.

Increased expression of genes indirectly associated with drug resistance was observed upon exposure of *S. aureus* to multiple aPDI doses (< 3 log_10_ reduction in viability). Within this study, upregulation of global transcriptional regulator genes involved in sensing the oxidative stress: *mgrA* (3.3-fold), *saeS* (4.00-fold), and the *fmtA* (2.17-fold) gene involved in the modification of teichoic acid and methicillin resistance, was observed. Furthermore, the greatest change in expression of the gene linked with increased resistance to fosfomycin (*fosB*) via enzymatic activity (10.2-fold) was also in this study, as evidenced [20]. A study conducted by Manoil et al. presented the effect of TMPyP and blue light on various genes in *E. faecalis*, and RT-qPCR method analysis confirmed the increased expression of *vanA*: 2.8-fold for isolate A1 and 3.3-fold for isolate A2 upon TMPyP-aPDI (1–3 log_10_ reduction in viability) [77]. Only one study, conducted by Müller et al., evidenced the increased expression connected with the resistance gene for Gram-negative representatives. Indeed, blue light irradiation (no data regarding the survival rate) affected in *A. baumannii* the DJ41_1581.t01 gene encoding carbapenem-associated resistance protein by increasing expression by 2.67-fold in comparison to non-treated cells [49]. On the other hand, many studies described below demonstrate a reduction in the expression of genes associated with antibiotic resistance and its mechanisms following exposure to aBL/aPDI. One of the pieces of evidence is an experiment carried out by Yang et al., who provide proof of the downregulation of a gene, *blaZ*, which is responsible for the resistance to beta-lactams. This conclusion was drawn for MRSA exposed to aBL (460 nm) treatment (bacterial reduction < 1 log_10_) [116]. Furthermore, Wong et al. investigated the drug-resistant gene expression in MRSA clinical isolates. Thus, for tested strain exposed to ICG and near-infrared light RT-qPCR method revealed for the *mecA* (conferring methicillin resistance in *S. aureus* and coagulase-negative staphylococci) a 2-fold reduction in the gene expression [124, 125]. In addition, Snell et al. during the 7-day experimental exposure of *S. aureus* to MB and visible light (reduction > 3 log_10_) achieved the downregulation of expression of gene (SAOUHSC_00647) related to Multidrug resistance ABC transporter ATP-binding (4.9-fold) [20]. For the Gram-negative representative, Walker et al. indicate that exposure of *C. jejuni* to aBL (reducing the bacterial dose viability by 1–3 log_10_) resulted in a 3.23-fold change and 9.73-fold change reduction in *cj1180c* gene expression (encoding ABC transporter ATP-binding) [22]. Furthermore, a study conducted by Jiang et al. utilized a novel blue light-controllable gene expression system in *E. coli* to regulate the genes responsible for antibiotic resistance; therefore, exposure of bacteria to blue light served as the regulatory agent. Upon exposure to aBL dose (no reduction of bacterial viability), the *bla* gene (encoding the beta-lactamase) underwent a significant decrease in expression (approx. 2.7-fold). Consequently, the resistance of the engineered *E. coli* to ampicillin was effectively reduced [126]. Furthermore, in another Gram-negative representative, *A. baumannii*, upon exposure to Rutin-Gal(III) complex- and Quercetin-mediated aPDI (reduction > 3 log_10_ and 1–3 log_10_), reduced expression of *bla*_*OXA−23*_, which confers resistance to oxacillin and other beta-lactams in *A. baumannii.* Consequently, exposure to aPDI with Rutin-Gal(III) resulted in a decrease of the expression by 4.1-fold, while Quercetin as PS with light reduced by a 7.8-fold gene expression [127]. It is noteworthy that the study conducted by Yue et al. on acid-fast bacteria *Mycobacterium abscessus* revealed alterations in the expression of the *erm* gene, responsible for resistance to macrolides, lincosamides, and streptogramin B antibiotics, and the gene *whiB7*, which functions across *Mycobacterium* species, is related to the mechanism limiting the activity of antibiotics [128, 129]. During the irradiation with two different concentrations of ALA: 40 and 100 mg/mL, the reduction of *whiB7* and *erm* relative expression decreased: approx. 4-fold (for 40 mg/mL ALA), 10-fold (for 100 mg/mL ALA), and approx. 2-fold (for both ALA concentrations), respectively [130].

The full list of differentially expressed genes regarding antimicrobial resistance is provided in Supplementary materials (Table S5.) and selected representative genes are presented in Fig. 4.

### Efflux pumps

Efflux pumps are transport systems that actively extrude drugs, toxins, and detergents from bacterial cells via specific membrane-associated proteins. Multiple families of microbial efflux pumps (MEPs) have been identified, including those in Gram-positive species such as *S. aureus* (e.g., NorA or QacA). Major MEP families include the multidrug and toxic compound extrusion family (MATE), ATP-binding cassette superfamily (ABC), and small multidrug-resistance family (SMR) [131]. Due to the rising prevalence of antimicrobial resistance, alternative approaches, including aPDI, have been developed to inhibit efflux pump activity. For example, photoinactivation using hybrid compounds like the NorA efflux pump inhibitor-methylene blue (EPI-MB) has been shown to disrupt the efflux-mediated resistance mechanism [132, 133]. Below, literature data are presented that include changes in the expression of efflux pump genes under the influence of oxidative stress caused by photoinactivation.

In the context of the observed alterations in the expression of genes coding efflux pumps, the research conducted by Yue et al. highlights the upregulation of efflux pump genes, specifically MAB_1409c (approx. 4-fold) and MAB_3142c (approx. > 10-fold), in response to ALA (100 µg/ml) and light treatment (reduction viability < 1 log_10_) in *Mycobacterium abscessus* [130]. Contrary to this, in a separate study, *A. baumannii* exhibited a decline in the expression of *emrA* (4.76-fold) and *emrB* (2.48-fold), which encode the components of an efflux pump of the resistance-nodulation-division (RND) upon blue light treatment (unknown effect on bacterial viability) [49]. This observation can explain the increased susceptibility to minocycline and tigecycline upon aBL treatment [45]. As exemplified by Yu et al., inhibition of genes encoding the efflux pump expression is affected by TBO and red light (reducing viability by 1–3 log_10_) [134]. Therefore, for *S. aureus* strains, the Multidrug efflux MFS transporter genes such as *mdeA* and *mepA* were upregulated: in MRSA by 1.59-fold and 6.29-fold, respectively, and in MSSA by 1.77-fold and 1.57-fold, respectively. On the other hand, genes encoding efflux pumps: *norB* and *sepA* were downregulated upon aPDI treatment: 3.77-fold for MSSA, 3.02-fold for MRSA, and 6.14-fold for MSSA, 3.47-fold for MRSA, respectively. The expression of the *norA* gene encoding the quinolone resistance protein NorA differed between MRSA and MSSA strains; thus, the *norA* gene was upregulated in MRSA (2.80-fold) and downregulated in MSSA (5.44-fold) [134]. In the last study, an altered expression of a gene (*cj1180c*) related to the MATE transporter family was identified in *C. jejuni*. Exposure of a Gram-negative bacterium to (T1) bacteriostatic (no reduction of the bacterial viability) and (T2) bactericidal (reduction of the viability 1–3 log_10_) doses of aBL increased *cj1180c* expression by 3.23- and 9.73-fold, respectively [22].

The full list of differentially expressed genes regarding efflux pumps is provided in Supplementary materials (Table S5.) and selected representative genes are presented in Fig. 4.

### Microbial cell envelope

The structural composition of bacterial cell envelopes plays a critical role in determining the effectiveness of photoinactivation. Both Gram-positive and Gram-negative bacteria possess negatively charged cell surfaces; however, their envelope architectures differ markedly. Gram-positive bacteria have a thick peptidoglycan layer embedded with teichoic and lipoteichoic acids, contributing to the overall negative surface charge and facilitating the binding and penetration of PSs. In contrast, Gram-negative bacteria possess an additional outer membrane rich in polysaccharides, which serves as a selective permeability barrier and hinders photosensitizer uptake [135, 136]. Oxidative stress has been demonstrated to cause structural and chemical damage to bacterial membranes: compromises integrity and disruption, cell wall decomposition, peroxidation of lipids, and cross-linking of proteins, further impairing membrane function and contributing to cell death [136–139]. Consequences of oxidative stress action upon phototreatments are, e.g., peptidoglycan and membrane destabilization, change of surface properties, reduction of the wall stiffness, and creation of gaps [137, 139]. The following studies illustrate the changes that occur in genes associated with the bacterial cell wall and cell membranes as a result of oxidative stress induced by photoinactivation.

In a study by Adair et al., the exposure of MRSA isolates to blue light (unknown effect on bacterial viability) revealed approx. 5.03-fold upregulation in expression of the *srtB* gene, encoding enzyme sortase - crucial for the appropriate anchoring and sorting of the cell surface proteins [117, 140]. In another study, multiple treatments of MRSA with 15 cycles of sub-lethal aBL (1–3 log_10_ reduction in viability) resulted in the upregulation of the expression of genes crucial in peptidoglycan formation. Transcriptomic analysis with RNA-seq and RT-PCR revealed significant upregulation of *glmS*, and downregulation of *ssA* by 2.68-fold/ 3.11-fold, and 3.99-fold/ 2.14-fold, respectively. Additionally, the RT-PCR analysis revealed the increased expression of *pbpB* (2.21-fold) and *murG* (1.22-fold), both of which are essential in peptidoglycan biosynthesis. Similarly, the *atl*, contributing to cell wall hydrolysis, exhibited a 1.72-fold decrease in expression [141]. Conversely, Snell et al. reported in *S. aureus* cells treated with a single aPDI-MB dose (reduction > 3 log_10_), and in a group of “tolerant” *S. aureus* increased expression of genes encoding the capsular polysaccharide synthesis enzymes: *cap5I*,* cap5J*, c*ap5*K (3.36-fold, 4.29-fold, and 4.23-fold change, respectively) [20]. Similarly, in another study, exposure of MRSA to aBL (reduction of viability < 1 log_10_) also upregulated important genes for capsule synthesis: *capA*, *capB*, *capC*, *capD*, *capF*,* capG*,* cap8K*, and *capL* (no data referring to fold-change) [116]. Additionally, the same study presented upon aBL increased expression of dehydrosqualene synthase (*crtM*) and dehydrosqualene desaturase (*crtN*) involved in the carotenoid biosynthesis and antioxidant defense in *S. aureus* [116, 142]. Furthermore, Yang et al. presented that aBL exposure led to the upregulation of the *SAR1973* (unknown fold change and survival rate change upon treatment), which encodes a membrane protein in *S. aureus* [116]. A wide range of studies for Gram-negative microorganisms have reported changes in the expression of *ompA*, which is associated with the transport of molecules across the outer membrane and plays a role in cell adhesion, biofilm formation, and modulation of the immune response [143]. In the study by Boluki et al., *A. baumannii* exposure to TBO-aPDI (no data referring to viability) led to an approximately 10-fold increase in *ompA* expression compared to the control [81]. Furthermore, increased expression of the *ompA* (3.27-fold) was also observed in a study conducted by Mueller et al., who irradiated *A. baumannii* with blue light (no data regarding the reduction) [49]. Subsequently, exposure of clinical and reference strains of *A. baumannii* to a TBO-aPDI (no data indicating the reduction of cells) resulted in a clinical isolate (resistant to colistin), a 31-fold upregulation of the *lpsB* (important for outer membrane integrity), and a 9-fold increase in the reference strain [43]. In a similar investigation by Boluki et al., an *A. baumannii* colistin-resistant clinical isolate exposed to TBO and light (no data referring to the reduction) was examined in terms of the expression of genes: *pmrA* and *pmrB*, which are part of the two-component system (PmrAB) and contribute to colistin resistance through modification of the lipid A. RT-qPCR analysis revealed decreased expression of *pmrA* and *pmrB*: 6.1- and 4.9-fold, respectively [144]. In the study presented by Khan et al., *E. coli* in an early stationary phase was exposed to blue light (no reduction in viability), which led to an increase in the expression of genes responsible, e.g., for the transportation of long-chain fatty acids and biosynthesis of membrane phospholipids (*fadL*,* fadJ*,* fadI*,* fadH*,* fadE*, and *fadD*) by 69.7-, 92-, 49.8-, 8.3-, 31.3-, and 53-fold, respectively [145]. A recent study by Kruszewska-Naczk et al. reported the 2-fold upregulation of the *dacA* gene involved in peptidoglycan synthesis in *E.coli* after treatment with two doses of aBL at 409 and 415 nm (reduction of survival rate by < 1 log_10_). Observed results may suggest that *dacA* confers protection against aBL-induced stress, through the activity of DacA in repairing peptidoglycan structure [23]. Subsequently, Jiang et al. created a blue light-controllable gene expression system in *E. coli*, referring to the changes in expression of the mechanosensitive channel of large conductance (MscL), which protects the cell from lysis during the acute downward shifts in the osmotic pressure. Upon the aBL illumination, the *mscL* gene was expressed to a greater extent (approx. 7.5-fold for DHMD-RAIM and 22-fold for DHMD-RAI2M), and the result of this gene expression modification was reflected in the increased susceptibility of the *E. coli* strain to streptomycin (due to participation of MscL in streptomycin transport) [126].

The observed decrease in resistance gene expression in both Gram-positive and Gram-negative microorganisms following photoinactivation, in the context of the growing problem of antibiotic resistance, provides important evidence that light-based approaches may be an alternative and helpful tool against AMR. A decrease in expression of AMR-related genes can result in increased susceptibility of microorganisms to antibiotics, for example, by limiting the efflux of antibiotics out of the cell, decreasing of hydrolysis β-lactamase ring or suppressing *whiB7* transcriptional activator in *Mycobacterium spp.*, which promotes intrinsic resistance determinants (e.g., acyltransferases modifying antibiotics) [122]. Furthermore, altered expression of regulatory genes (particularly global regulators) can influence multiple cellular pathways involved in stress adaptation. While these regulators help to cope bacteria with harmful conditions, on the other hand, they are also closely related to antimicrobial resistance, virulence factors production, and toxins, etc. For example, in the study described above, the regulatory gene *mgrA* upon oxidative stress exhibited increased expression, which in consequence led to modulation of the membrane permeability and efflux systems, thereby favoring the inhibition of antibiotic efflux. Furthermore, the overexpression of resistance genes associated with efflux pumps, transporters, and related mechanisms upon oxidative stress may reflect bacterial adaptation to environmental changes upon ROS [146]. For instance, increased expression of the *fmtA* counterpart environmental adaptation, which helps to stabilize the bacterial cell in the presence of oxidative stress, simultaneously reduces the antibiotic effectiveness [147]. Additionally, increased expression of regulatory genes can also recruit other antimicrobial resistance mechanisms involving genes, e.g., *norA*,* norB*, due to multiple regulatory functions of gene *mgrA* [148].

Overall, the observed increases or decreases in the expression of genes related to drug resistance and efflux pumps represent a cross-reaction encompassed by cell reprogramming in response to oxidative stress.

It is also worth noting that many of the studies described lacked detailed information on how the doses of aBL or aPDI, particularly when using extracellular photosensitizers, affected bacterial survival, as well as on the exact fold-change values of gene expression upon ROS presence. This significantly hinders the ability to integrate obtained results and to correlate observed gene expression changes with decreased survival.

Furthermore, the lack of unified methodology and standardized protocols (even aBL or aPDI) may influence the reported outcomes, often resulting in insufficient CFU reduction and discrepancies in the expression patterns of the same genes across different studies. Nevertheless, many of the studies indicate significant alterations in the cell membrane, efflux pumps, and HSPs. These findings indicate that the photodynamic method effectively affects various cellular components and how Gram-positive and Gram-negative bacteria cope with the negative aspects of oxidative stress to maintain viability and adapt to its conditions (particularly through the protective responses).

The full list of differentially expressed genes regarding microbial cell envelope is provided in Supplementary materials (Table S6.) and selected representative genes are presented in Fig. 4.

## Cellular metabolism and other processes

aPDI can affect the expression of several bacterial genes associated with oxidative stress response, DNA repair, as well as the biosynthesis of crucial bacterial metabolites. Indeed, several metabolic pathways involved in energy metabolism, fermentative pathways, cell division, and replication may be altered by aPDI and aBL [149, 150] as bacteria instantly adapt to these conditions to facilitate their survival. Oxidative stress causes molecular damage, triggering compensatory biochemical responses, and bacteria adjust their metabolism and amino acid composition to maintain homeostasis. Identifying metabolic pathways altered by lethal or sublethal doses of aPDI and aBL can significantly contribute to the optimization of these therapies. For example, by combining phototherapy with metabolic pathway inhibitors and selecting PSs that target vulnerable metabolic components, or adjusting treatment parameters to enhance cellular damage.

One such metabolic target is the group of enzymes known as glucosyltransferases (Gtfs), which catalyze the conversion of extracellular sucrose into exopolysaccharides (EPS), playing a critical role in biofilm formation by *S. mutans* [151]. In addition to their structural function, Gtfs are also involved in carbohydrate metabolism, as glucans produced by these enzymes can serve as an energy reservoir under nutrient-limited conditions. Given their central role in both pathogenicity and survival of *S. mutans* within the oral cavity, Gtfs has become an attractive target for anti-virulence strategies aimed at selectively inhibiting pathogenic mechanisms without disrupting the resident microbiota [152]. In this context, several aPDI studies have focused on the downregulation of gtf-encoding genes [67]. *P. gingivalis*, a Gram-negative biofilm-forming pathogen associated with periodontitis and gingivitis, has also been targeted by aPDI. In the study using a DNA aptamer-nanographene oxide system, expression of key virulence genes *rgpA* (arginine protease) and *fimA* (fimbrilin) was significantly decreased following aPDI, by 6.8- and 10.4-fold at a dose that reduced viability by > 3 log_10_ [153]. Although both genes are primarily related to the virulence of *P. gingivalis*, they are also closely linked to its metabolism. For example, *rgpA* contributes to polysaccharide capsule synthesis, surface protein processing, and hemoglobin degradation [154], while *fimA* takes part in adhesion and biofilm formation, which in turn facilitates amino acid synthesis. Interestingly, *rpgA* in *P. gingivalis* was also downregulated after aPDI with MB (4.9-fold), TBO (11.6-fold), and ICG (14.0-fold) at doses that caused no statistically significant reduction in bacterial viability [155]. For the latter two, the observed relative change in mRNA expression was significant compared to the control. On the other hand, a study conducted by Yuan et al. on the transcriptomic response of *P. gingivalis* to the aBL (> 3 log_10_) revealed that *rpgA* as well as *rpgB* were substantially upregulated, by 7.41- and 8.60-fold, respectively [21]. This may reflect elevated heme acquisition driven by increased intracellular ROS. As both genes are critical for the conversion of oxyhemoglobin to methemoglobin, a better source of heme for *P. gingivalis*, this regulation allows bacteria to maintain the iron homeostasis and counteract the oxidative stress. The same study also reported upregulation of *fetB* (2.39-fold) and *ftn* (3.10-fold), likely linked to increased intracellular iron levels. For instance, *fetB* encodes an ABC-type iron-export component [156] that also acts as an iron chelatase, removing it from heme after its acquisition [157]. *Ftn* therefore encodes ferritin-like protein, which takes part in iron storage, so upregulation of both genes helps maintain iron homeostasis and counteracts oxidative stress-induced damage [158].

Another mechanism associated with the maintenance of metal cellular balance is connected with the expression of metal-specific efflux pumps such as *fieF*, which encodes a ferrous-iron efflux pump. In a study using nano-chitosan encapsulated ICG for aPDI against *A. actinomycetemcomitans*, *fieF* was significantly downregulated (14.8-fold), at a dose that resulted in < 1 log_10_ decrease in viability [159]. The impact of aPDI on the expression of genes associated with iron metabolism was also evaluated in the case of critical human pathogens. For instance, Snell et al. examined the tolerance and genomic response of *S. aureus* to sublethal (> 3 log_10_ reduction) doses of MB [20]. Interestingly, they found that strains with induced tolerance are characterized by higher expression of iron/heme transporting and uptake genes such as *efeO* (Iron uptake system component protein, *FeABC* (ATP-binding cassette (ABC) transporter), and *htsB* (Heme ABC type transporter permease), by 3.81-, 4.60-, and 2.96-fold, respectively. These changes were also associated with overexpression of iron transport siderophores, namely SirA (1.71-fold) and SirB (1.61-fold). An interesting study involving blue light treatment and temperature on iron metabolism in *A. baumannii* was conducted by Tuttobenne, Crib, and Mussi [160]. They showed that the expression of Fur-regulated Acinetobactin cluster genes such as *basA*,* bauD*,* basE*,* bauA*, and *basD* was induced only in the dark and only when BlsA photoreceptor was present, while iron metabolism modulation by light disappeared at elevated temperatures. However, quantitative data specifying exact fold-change values were not provided. This topic is much more interesting when taking into account another study, where doses of aPDI using TBO and LED (which did not cause a statistically significant reduction in cell viability) were found to induce *blsA* expression by 2.80-fold in extensively resistant *A. baumannii* [45]. Temperature-dependent gene regulation motivated Squire et al. to perform RNA-seq analysis on *A. baumannii* grown at 37 °C with or without illumination [47]. Interestingly, light exposure at this temperature resulted in downregulation of *benP* (2.85-fold) and *catA* (4.88-fold), which mediate benzoate transport and metabolism. Conversely, exposure to light under elevated temperature was associated with upregulation of several lipid-metabolism genes, including a GDPD glycerophosphodiester phosphodiesterase ortholog.

In a study conducted by Muehler et al., a wide-ranging RNA-seq of *E. coli* treated with SAPYR-based aPDI (< 1 log_10_ reduction in viability) revealed that half of the most downregulated genes were linked to formate metabolism, namely, *hycB* (52-fold), *hycC* (34.3-fold), *hycF* (encoding formate hydrogenlyase subunits 2, 3, and 6, 32-fold), *fdhF* (formate dehydrogenase, 30-fold), *hydN* (electron transport protein HydN, 45.3-fold), and 1 that encodes fumarase (*fumB*, 30-fold) [100]. This might be related to the fact that *E. coli* under anaerobic conditions utilizes formate hydrogenylase complex (FHL) and fumarase that contain iron-sulfur (Fe-S) clusters, which are sensitive to oxidative damage [161, 162]. Moreover, this could be associated with the shift in metabolic priorities as well as global regulatory effects that could mitigate the oxidative stress-induced damage.

Another comprehensive evaluation was conducted by Park et al., who performed the transcriptional profiling of *S. aureus* exposed to a dose (causing no statistically significant reduction) of aPDI involving chlorin e6 (Ce_6_) and a diode laser [163]. This study showed that *S. aureus* responds to photo-oxidative stress primarily through the Agr regulator. The most upregulated gene was *geh* (12.1-fold change), encoding staphylococcal lipases involved in both metabolism and virulence, including immune invasion, through direct hydrolysis of lipoprotein pathogen-associated molecular patterns (PAMPs) [164]. Furthermore, several genes that are involved in the degradation of amino acids, such as *hutI* (imidazolonepropionase, 5.2-fold) and *hutU* (urocanate hydratase, 6.7-fold), which degrade histidine or *arcC2* (carbamate kinase, 2.1-fold) and *arcD* (arginine/ornithine antiporter, 2.2-fold), which catalyze the arginine conversion to ornithine, ammonia, and CO_2_ in ATP production [165] were found to be upregulated. Additionally, *pckA* encoding phosphoenolpyruvate carboxykinase was also overexpressed (4.8-fold) [163]. This confirms that *S. aureus* in response to aPDI-induced oxidative stress, exploits mechanisms allowing it to utilize alternative energy sources. In the case of *S. aureus*, not only can aPDI lead to the utilization of intracellular machinery promoting survival. Interestingly, Adair and Drum have conducted RNA-seq analysis to investigate gene expression patterns in response to blue light irradiation (no data about the decrease in viability are provided) [117]. In their study, a list of 32 genes that change upon blue light irradiation has been proposed, indicating the critical genes associated with staphylococcal metabolism. The most significant upregulation was found for *acpD* (azoreductase) and *PcpA_N-like* (dioxygenase) with 28.06- and 18.32-fold change upon aBL treatment. This may be related to the role of these enzymes in electron transfer, reduction of oxidative damage led by quinones or other intermediates involved in the respiratory chain (azoreductase) or DNA/lipid repair caused by ROS (dioxygenase) [166, 167]. In contrast, other genes associated with metabolism (e.g., biosynthesis of amino acids) have been downregulated, including *asd* (semialdehyde dehydrogenase, 5.54-fold) or *lysC* (aspartate kinase, 5.3-fold).

For the vast majority of pathogens exposed to aPDI or aBL, the gene expression studies mostly focus on virulence genes, oxidative stress response, and DNA repair. However, sometimes genome-wide analyses reveal affected pathways and photooxidative regulators. Recently, Kuroyanagi et al. showed that *Vibrio parahaemolyticus* responds specifically to blue light by upregulation of genes responsible for iron-sulfur biosynthesis linked to ROS generation, without altering pathogenicity genes [168]. On the other hand, metalloregulatory-like protein (MerR) regulators and anti-sigma factor (ChrR), typically associated with *V. chole*rae virulence [169, 170] were identified as major blue light-response regulators [101]. Moreover, RNA-seq revealed strong upregulation of several metabolic or signal transduction pathways (e.g., TCA cycle and propanoate metabolism, lysine degradation, oxidative phosphorylation, and fatty acid degradation, while pathways related to chemotaxis, two-component system, glyoxylate and dicarboxylate metabolism, and ABC transporters were markedly downregulated.

Among bacterial pathogens responsible for gastrointestinal tract infections, *C. jejuni* has significant evidence of causing gastroenteritis globally. However, studies focusing on these bacteria are relatively limited [171]. Its susceptibility to light has prompted research on light-induced inactivation [172–174]. The most extensive analysis by Walker et al. used RNA-seq to assess global gene expression in response to aBL at two time points: 15 min (T1, no statistically significant reduction in bacterial load) and 30 min (T2, 1–3 log_10_ decrease in viability) [22]. Although the most important upregulation was noticed for genes associated with oxidative stress response, several metabolism-associated genes were induced upon blue light exposure. For example, genes encoding iron transport, such as *chuA* and *chuB*, were upregulated by 2.15- and 17.92-fold and by 1.72-and 4.44-fold after 15 and 30 min of exposure, respectively. Similarly, genes encoding reductases for alternative electron acceptors other than oxygen, including as *mfrABE* (periplasmic fumarate reductases subunits A, B, E, 2.15-and 17.92-fold; 4.84- and 4.30-fold; and 2.22-fold) *dcuAB* (3.72- and 2.44-fold, and 2.04- and 2.17) and *frd* (cytoplasmic fumarate reductase system, approx. 1.2-fold for both exposure times), and *cj0264c/265c* (periplasmic TMAO reductase, 4.37- and 4.07-fold, and 6.90- and 8.40 after 15 and 30 min, respectively), were upragulated. Interestingly, the change of *napG* (nitrate reductase G) depended on time of exposure (↑2.08-fold and ↓1.07-fold after 15 and 30 min of exposure, respectively), while *napH* (nitrate reductase H) was upregulated (2.87- and 1.65-fold, respectively). On the other hand, genes associated with amino acid synthesis, such as *argB* (acetylglutamate kinase) and *argC* (N-acetyl-gamma-glutamyl-phosphate reductase) or *kdtA* encoding 3-deoxy-D-manno-octulosonic-acid transferase, were downregulated by 4.82- and 3.39-fold, 4.83-and 8.94-fold, 7.15-and 11.49-fold for 15 and 30 min of exposure, respectively.

The observed gene regulation across these bacterial species suggests a shared adaptation mechanism to aPDI or aBL, prioritizing energy production and oxidative stress response over growth. In *C. jejuni*, upregulation of certain genes points to increased anaerobic respiration and iron acquisition, while downregulation suggests a transition from growth to survival.

**Fig. 5:**
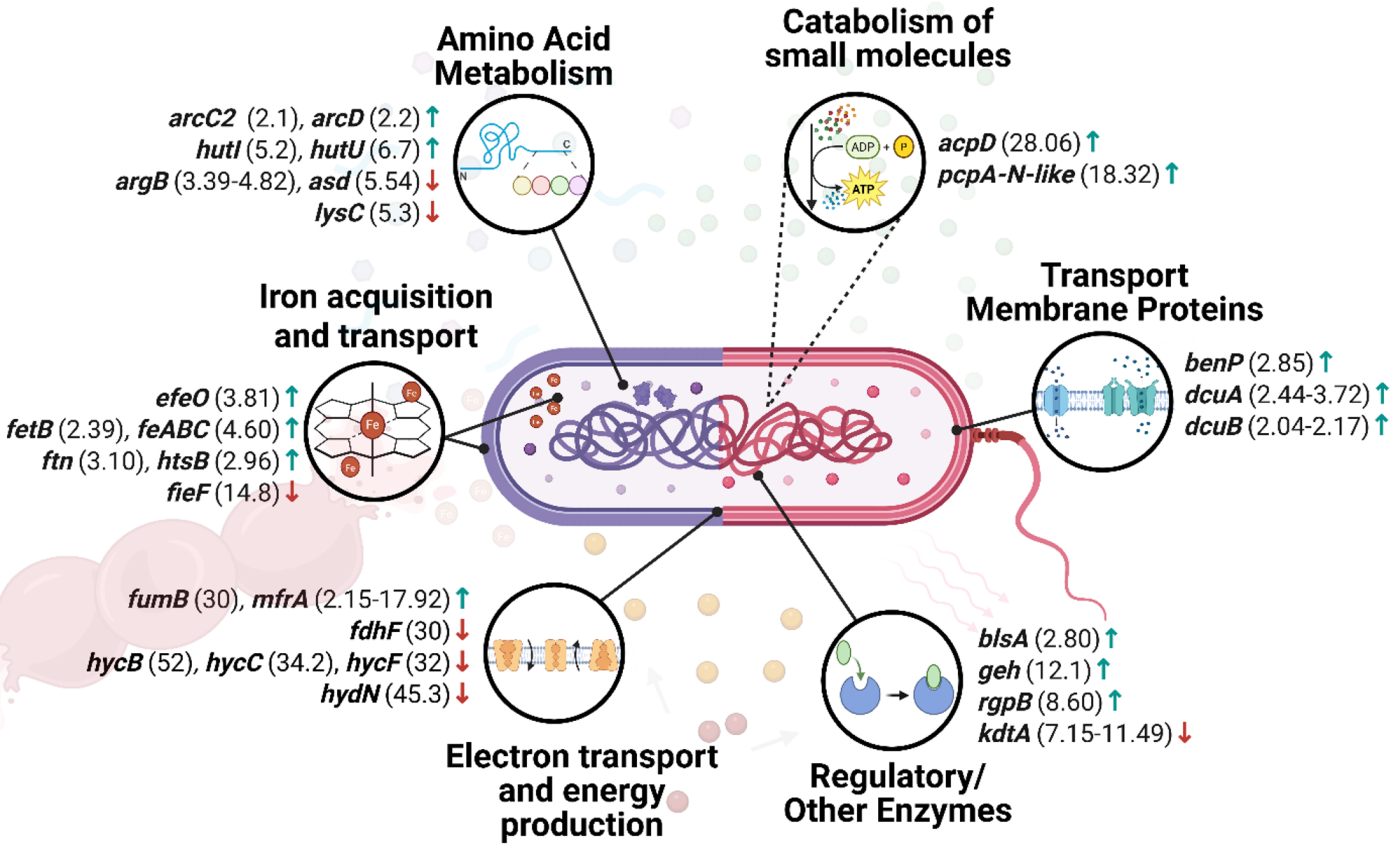
Graphical representation of selected genes altered after aBL/aPDI treatment regarding cellular metabolism and other processes. Arrows indicate direction of change, i.e., “↓” represents downregulation of a particular gene, while “↑” represents upregulation. Fold-change values shown in the figure represent the range between the lowest and highest reported magnitude of transcriptional change across the available studies for a given gene, unless only a single study was available. The bacterial species in which differential expression was reported are as follows: *arcC2*, *arcD*, *hutI*, *hutU*, *asd*, *lysC*, *efeO*, *feABC*, *htsB*, *acpD*, *pcpA-N-like*, *geh* - *S. aureus*; *argB*, *mfrA*, *kdtA*, *dcuA*, *dcuB* - *C. jejuni*; *fumB*, *fdhF*, *hycB*, *hycC*, *hycF*, *hydN* - *E*.*coli*; *fetB*, *ftn*, *rgpB* - *P. gingivalis*; *fieF* – *A. actinomycetemcomitans*; *bslA*, *benP* – *A. baumannii*

The full list of differentially expressed genes regarding cellular metabolism is provided in Supplementary materials (Table S7.) and selected representative genes are presented in Fig. 5.

## Potential mechanisms involved in light tolerance

While many studies describe transcriptional changes following aPDI or aBL exposure as part of an acute oxidative stress response, a subset of reports suggests that repeated sublethal phototreatments may promote adaptive transcriptional programs associated with increased survival. In this context, changes in the expression of genes involved in detoxification, redox homeostasis, and global stress regulation may not only reflect immediate damage control but also contribute to the emergence of light-tolerant phenotypes. This section summarizes studies in which gene expression patterns were explicitly linked to repeated photodynamic exposure or experimentally defined tolerant populations, and discusses their potential mechanistic implications.

One of the first studies directly linking transcriptional changes to the development of light tolerance was reported by Pierański et al. [94]. The study revealed that the changes in expression of genes in *S. agalactiae* exposed to multiple doses of RB-mediated aPDI may contribute to the tolerance development to photoinactivation. Accordingly, the study focused on genes involved in cellular detoxification. The authors demonstrated that a sublethal dose of RB-aPDI (< 1 log_10_) led to increased expression of *sodA* (5-fold), *tpx* (thiol peroxidase, 2.5-fold), or r*ecA* (recombinase A, 3-fold) and decreased expression of *ahpC* (alkyl hydroperoxide reductase C, 2-fold) and *cylE* (granadaene, β-hemolysin/cytolysin, carotenoid pigment, 5-fold). However, increased exposure in consecutive cycles to RB-aPDI resulted in more significantly increased expression of *sodA* (20-fold), *npx* (20-fold), *tpx* (3-fold), and *recA* (20-fold). This conclusion was drawn for tolerant *S. agalactiae* cells (treated for 10 days with aPDI) exposed additionally to aPDI. Overall, the presented changes in gene expression for the tolerant population may indicate a potential mechanism of tolerance to aPDI [94]. Subsequent study by Snell et al. reported changes in the expression of the genes responsible for response to oxidative stress and detoxification in *S. aureus* exposed daily to control treatments (with no PS and light exposure), followed by a single MB-aPDI treatment (resulting in a < 3 log_10_ decrease in viability) during a 7-day cycle. In this study, the successive 7-day sequential MB-aPDI treatments, followed by a single aPDI treatment (as multiple treatments), were also implemented. All the irradiated as well as control samples from day 7 were examined with the RNA-seq method. Depending on whether *S. aureus* was treated with single or multiple MB-aPDI (in 7 cycles of exposure), different genes were expressed. Repeated photodynamic exposure for 7 days (defined as: tolerant cells) resulted in upregulation of genes encoding a flavin mononucleotide (FMN)-dependent NADH-azoreductase (2.57-fold) - SAOUHSC_00173 and genes encoding manganese-dependent superoxide dismutase (upregulated in „tolerant” cells – 2.95-fold) - SAOUHSC_00093. Moreover, SAOUHSC_00833, which encodes a nitroreductase family protein, was upregulated in a “tolerant” cell (1.55-fold), and additionally, the SAOUHSC_02825 gene, encoding a glyoxalase family protein, was downregulated in the tested conditions (1.83-fold). The RNA-seq study also revealed in cells treated with a single MB-aPDI dosage downregulation of gene – SAOUHSC_00320 – encoding an NADH-dependent flavin mononucleotide reductase (1.90-fold), and SAOUHSC_00318, which encodes a glyoxalase/bleomycin resistance/ dioxygenase superfamily (1.38-fold). Increased expression of various genes related to detoxification in tolerant *S. aureus* cells may reflect stable adaptive changes arising during repeated cyclic exposure to photoinactivation, potentially contributing to enhanced survival under oxidative stress conditions. Besides genes described above, the iron transport siderophores *sirA* (1.71-fold), *sirB* (1.61-fold), whose function is related to ROS detoxification, were overexpressed, and *htsA* (1.99-fold), *htsB* (2.96-fold), and *htsC* (2.39-fold) in tolerant phenotypes [20]. Furthermore, global transcriptional regulators involved in sensing the oxidative stress, *mgrA* (1.73-fold) and *saeS* (2.0-fold), had increased expression, whereas the *csoR* (1.69-fold) repressor exhibited decreased expression [20]. Copper-sensing transcriptional repressor CsoR is involved in protection against the oxidative stress induced by the Fenton reaction due to the presence of copper [175].

In addition to studies directly examining repeated photodynamic exposure, several reports suggest that strain-dependent differences in stress response regulation may predispose bacteria to divergent outcomes following light-based antimicrobial treatments. The first study investigating gene expression changes associated with photoinactivation of microorganisms was carried out by Nakonieczna et al. [176]. Four clinical isolates of *S. aureus*, susceptible (≥ 3 log_10_ reduction in viability) and resistant (< 1 log_10_ reduction in viability) to photoinactivation, were exposed to protoporphyrin IX and red light. aPDI treatment resulted in the increased expression of the *sodA* (13.5-fold for 472 strain, and 20.0-fold for 80/0 strain) and *sodM* (41-fold for 472 strain, and 4.1-fold for 80/0 strain), encoding superoxide dismutases, for *S. aureus* strains susceptible to aPDI, whereas resistant strains did not exhibit comparable transcriptional induction. Importantly, the absence of enhanced survival in strains displaying elevated Sod activity indicated that induction of oxidative stress response genes alone does not necessarily confer protection against photodynamic damage.

A complementary regulatory pattern was reported by Kim and Yuk, who compared gene expression responses in *S. enterica* serovar Enteritidis (light-sensitive) and serovar Saintpaul (light-resistant) following 405-nm LED illumination. Notably, the transcription level of *oxyR* increased 1.5 times in illuminated *S. Enteritidis* cells but decreased 1.4 times in illuminated *S. Saintpaul* cells compared to nonilluminated controls. However, the broader upregulation of stress response genes in *S. Saintpaul* may partially contribute to its higher resistance to light exposure [18].

**Fig. 6:**
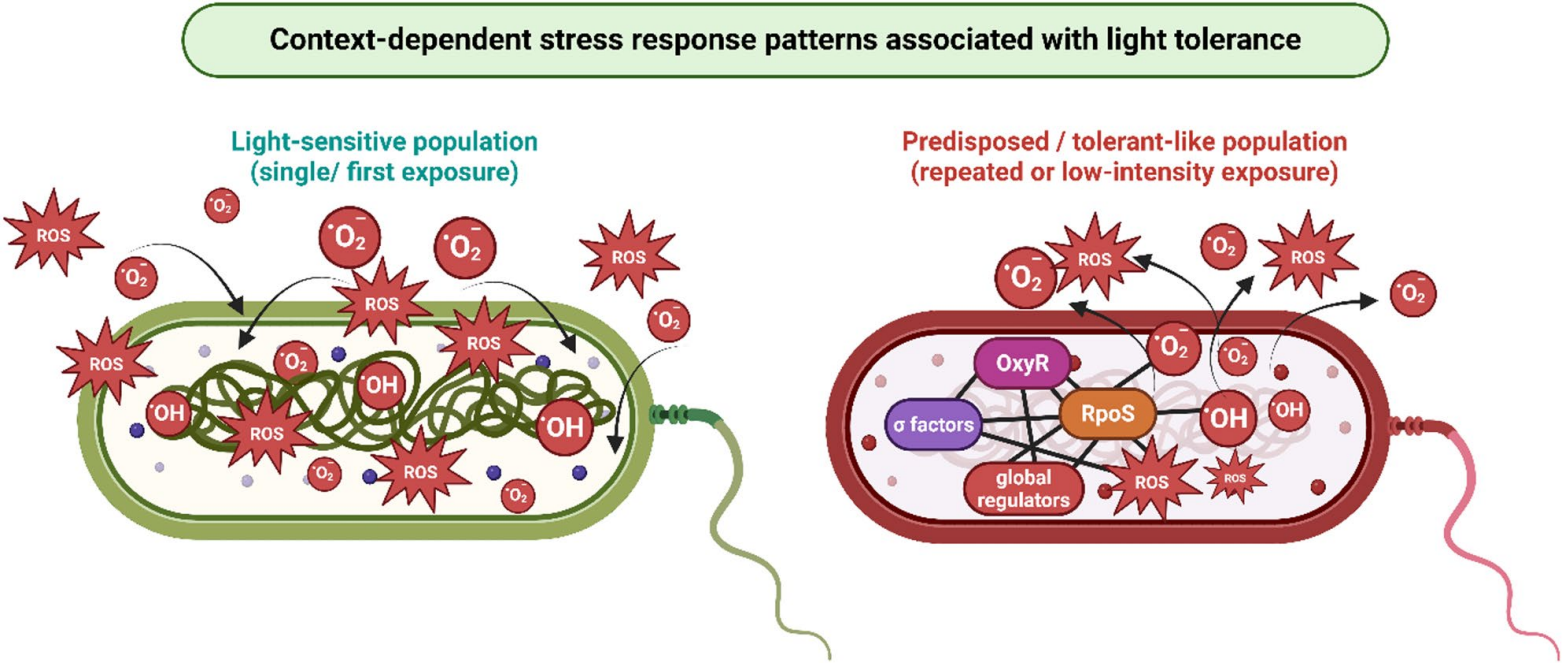
Conceptual model of context-dependent stress response patterns associated with bacterial light tolerance. In light-sensitive populations subjected to a single or first exposure to aBL or aPDI, photoinduced reactive oxygen species (ROS) trigger acute oxidative damage, resulting in widespread molecular stress and limited activation of protective regulatory networks. In contrast, repeated or low-intensity exposure may promote the emergence of predisposed or tolerant-like populations characterized by coordinated activation of global stress regulators (e.g., OxyR, RpoS, alternative sigma factors) and detoxification pathways, leading to improved redox homeostasis and enhanced survival under subsequent light stress

Collectively, these studies suggest that the differential organization of oxidative stress response networks (whether inducible or constitutively active) may influence bacterial susceptibility to photodynamic treatments. Such regulatory patterns could represent predisposing factors that facilitate survival under repeated or sublethal light exposure, thereby contributing to the emergence of aPDI/aBL-tolerant phenotypes observed in cyclic treatment models. The full list of differentially expressed genes regarding the potential mechanism of tolerance is provided in Supplementary materials (Table S8), and a conceptual overview of these context-dependent stress response patterns is provided in Fig. 6.

## Summary

Microorganisms developed a diverse arsenal of factors that facilitate their colonization of host cells and evade immune responses. The overview of existing data revealed a general downregulation of virulence-associated genes, suggesting that photodynamic methods can not only eradicate bacteria but, hypothetically, also attenuate their pathogenicity. Unfortunately, despite numerous studies demonstrating aPDI/aBL-induced decrease in the functionality of various virulence factors as well as aPDI/aBL-induced downregulation of virulence factor genes [88, 177–180], there are no in vivo studies supporting the statement that aPDI/aBL indeed reduces the bacterial virulence in a host; thus, such investigations are urgently needed. Upregulation of particular genes may be explained by possible compensatory or adaptive mechanisms that help bacteria maintain competitiveness in changing environmental conditions. The discrepancies between reported results may also stem from differences in the applied methodology, like PSs, light wavelengths, or bacterial phase of growth. Though it is almost impossible to identify the most robust transcriptomic data in relation to specific PSs, light wavelengths, and bacterial species, we made an effort to demonstrate the most frequently repeated response patterns. In the case of numerous photosensitizers, e.g., porphyrins, phenothiazines, or xanthens, the induction of genes associated with oxidative stress and the SOS response is consistently observed, with a simultaneous decrease in the expression of enterotoxin genes and other virulence determinants [24, 94, 96, 100, 176]. It appears that aPDI repeatedly modulates virulence-related loci and detoxification/oxidative stress regulons [20, 25, 39, 41, 94, 96, 100]. aPDI with gallium porphyrin further illustrates this pattern by downregulating the expression of toxin-encoding genes and engaging the SrrAB two-component regulatory system in *S. aureus*, and by eliciting similar effects in *P. aeruginosa* [87, 88].

In the case of aBL, we also observe particularly strong signatures of strong induction of chaperone and oxidative stress genes and coordinated remodeling of central metabolism [22]. Similar aBL-induced upregulation of stress and DNA repair pathways, along with altered oxidative stress regulons, has been demonstrated in *S. enterica* and *V. cholerae* [18, 101]. Studies on *E. coli* and *C. sakazakii* also indicate induction of stress and heat shock response genes and repression of loci associated with biofilm and motility [23, 58, 59, 95].

The most strikingly consistent picture is observed in *S. mutans*. In at least seven studies using different classes of PSs, i.e., TBO, ICG, riboflavin, emodin-chitosan nanoparticles, *Ulva lactuca* extract, nano-quercetin, and other preparations, sublethal aPDI consistently causes a 4- to 8-fold downregulation of *gtfB*, and treatment with *Ulva lactuca* extract additionally inhibits *gtfC/D* [60–64, 66, 67]. The same series of experiments shows a consistent reduction in the expression of QS *comA–E* genes.

Enterococcal virulence genes are another group with highly reproducible responses. Multiple studies using ICG, TBO, MB, curcumin-based systems, rutin-Ga(III), kojic acid, and parietin have shown a significant decrease in the transcription of these genes after aPDI and/or aBL in *E. faecalis*, in both planktonic and biofilm models [70–76].

In the case of DNA repair involved genes, it is worth noting that most of the research presents results for the sublethal phototreatment of bacteria. These doses of light may not cause a massive accumulation of DNA damage, which can result in no significant increase in the expression of genes engaged in DNA repair systems. The first line of cellular defense could be a general reaction to oxidative stress and the SOS response system. Nevertheless, no direct investigations focused on changes in the expression level of genes engaged in DNA repair among different repair systems after phototreatment, which suggests a need for further investigations into this research problem.

All the studies regarding bacterial responses to oxidative stress induced by aBL or aPDI highlight the role of genes associated with oxidative stress response in protecting cells from damage caused by ROS. In particular, genes responsible for ROS detoxification and chaperones show significant changes in response to aBL or aPDI mediated by different PSs. Numerous studies indicate a complex bacterial response to oxidative stress, involving activation of protective systems against ROS, as well as changes in the expression of genes related to energy metabolism, cell division, and DNA repair pathways. Specifically, some studies emphasize the critical role of sigma factors and their interaction with other regulatory proteins in regulating gene expression in response to photo-induced oxidative stress. These studies suggest that sigma factors are a central factor in controlling the transcription of genes related to oxidative stress response in various bacterial species.

It is thought that the consequences of oxidative stress in the cell may be reflected in the form of direct changes in the expression of genes encoding antibiotic resistance, but importantly also in global regulators that also undergo changes in expression and influence the sensitivity of the microorganism to antimicrobial agents. Such an effect is indirect because the regulators (e.g., MgrA) may indirectly lead to changes in the expression of genes encoding efflux pumps. It has been suggested that the presence of photosensitising compounds may contribute to their increased efflux from the cell. Porins and outer membrane proteins are one of the targets of oxidative stress, leading to compensatory upregulation of the genes encoding them. For example, increased expression of the *A. baumannii ompA* gene was observed after aPDI treatment. In addition, porin expression in Gram-negative bacteria can be altered by stress sigma factors. On the other hand, in Gram-positive microorganisms, it has been found that oxidative stress can result in modulation of surface protein expression and cell wall synthesis. In addition, photoinactivation has been evidenced to result in changes in the expression of genes involved in the construction of membrane components, such as lipid synthesis and cell envelope biogenesis. It is also thought that membrane protein folding - periplasmic chaperones, to be precise - plays an important role in removing misfolded proteins from the membrane. Overall, it would seem that oxidative stress affects the formation of cell membrane compounds and the maintenance of membrane protein integrity.

## Future perspectives

As demonstrated throughout this review, the response to photodynamic inactivation and oxidative stress induced by aBL and aPDI is the result of a complex regulation of genes and cellular pathways. Moreover, the influence on bacterial tolerance and survival under oxidative stress conditions depends on a combination of factors such as exposure time, light dose, photosensitizer type, and the bacterial strain. As a result, identifying a consistent, repeatable pattern of transcriptomic response across studies remains extremely challenging.

Therefore, future research should prioritize the unification of experimental protocols and the standardization of sublethal treatment parameters, especially in terms of mechanistic analyses. Sublethal conditions should be non-eradicating to enable more accurate insight into microbial regulatory responses, free from effects of irreversible cell damage or lysis.

Beyond protocols and parameters, there is a clear need for the standardization of terminology. Various terms, such as antimicrobial photodynamic inactivation (aPDI), photodynamic antimicrobial chemotherapy (PACT), and photodisinfection, are used interchangeably, creating inconsistency in the literature and ambiguity in interpretation among researchers and clinicians. Notably, several European photobiology societies are actively engaged in initiatives aimed at developing a unified classification system and standardized nomenclature, which could significantly enhance the clarity and comparability of future studies.

Additionally, many current studies employed isolates obtained from the environment or patients, which have a great clinical relevance, but cannot be translated to mechanistic research, whereas they often lack the extensive genomic and genetic toolkits available for established model organisms. Thus, we encourage working on well-characterized species, such as *E. coli* as a Gram-negative representative or *S. aureus* as a Gram-positive one, which enables the use of isogenic mutants, reported strain, or full system-analysis approach. Findings from such models can then be extrapolated to more complex or less studied pathogens.

Next, the methodology applied for studying gene expression should advance toward more advanced and high-throughput approaches. The majority of the presented research utilized RT-qPCR, which remains a common, accessible, and cost-effective tool, but is limited to several targeted genes. What should be prioritized is the implementation of techniques that can provide insight into broader transcriptional dynamics and regulatory cascades. Moving toward comprehensive methods like RNA-seq will allow unbiased profiling of the entire transcriptome, uncovering novel genes, pathways, and regulatory elements involved in the microbial response to photodynamic stress.

Regarding future perspective, we also identify studies demonstrating the combination of gene expression with protein analysis as an extremely important issue to be investigated. It would be of high impact to demonstrate whether the gene expression outcomes from the cited literature above could indeed be translated into the protein level. Currently, only a limited number of studies integrate transcriptomics and proteomics in bacteria exposed to aPDI/aBL. Though none of these performed a full quantitative proteomic analysis, they provide direct evidence that photodynamic stress induces coupled transcript-protein responses [22, 100, 111].

For the future better applicability of the photodynamic methods resulting from the designing of optimal photosensitizing compounds, it would be of high interest to demonstrate which specific ROS photochemical reaction, namely, mechanism type I, II or III, leads to a specific molecular response in microbial cells. Providing such a deep insight into bacterial response to photooxidative stress would facilitate the design of a proper therapeutic protocol.

Finally, we identify the great need for studies bringing the transcriptomic data to the in vivo level.

Collectively, these efforts will be essential to advance the field toward a more unified, mechanistically informed, and translationally relevant understanding of microbial responses to photodynamic stress.

## Supplementary Information

Supplementary Material 1

## Data Availability

No datasets were generated or analyzed during the current study.
