# Supplementary material for: Illuminating the mechanism: gene expression responses to antimicrobial photodynamic therapy
Source: BMC Genomics. 2026 Feb 23;27:322. doi: 10.1186/s12864-026-12676-0 (PMC13037157; doi:10.1186/s12864-026-12676-0)
Supplement: Supplementary file 1 — Supplementary Material 1 [file 12864_2026_12676_MOESM1_ESM.docx]

**Supplementary data**

**Supplementary Table S1. Differentially expressed genes related to virulence upon photodynamic treatment.**

| **Gene/encoded protein** | **Function** | **Expression change** | **Method** | **Species** | **Source of bacteria** | **Photoinactivation** | **Decrease in viability** | **Refe-rence** |
| --- | --- | --- | --- | --- | --- | --- | --- | --- |
| *abaI* / acyl-homoserine-lactone synthase | Signal transduction, quorum sensing | ↓ Downregulated  (1.9-fold) | RT-qPCR | *A. baumannii* | ATCC 19606, Iranian Biological Resource Center | ICG-aPDI (31.2 µg/mL, 810 nm, 31.2 J/cm^2^) | No statistically significant reduction | [43] |
|  |  | ↓ Downregulated  (38-fold at 23°C)  ↑ Upregulated  (1.5 fold at 37°C) | RT-qPCR |  | ATCC 17978, clinical isolate | 462 nm blue light (6-10 mol photons/m^2^/s) | No data | [46] |
|  |  | ↓ Downregulated  (4.64-fold) | RT-qPCR |  | ATCC, National Microbial Bank of Iran, Pasteur Institute | HypNP@D-Trp-aPDI (1/2 x MIC, 450 nm blue light, 60s) | >3 log_10_ | [42] |
|  |  | ↓ Downregulated  (8.3-fold for CR-XDR-  AB)  (6.3-fold for ATCC  19606) | RT-qPCR |  | CR-XDR-AB –  burn-wound infection  ATCC 19606 – reference strain | TBO-aPDI (0.37 mg/mL, 630nm, 180 J/cm^2^) | No reduction (growth inhibition) | [45] |
| *ace* / adhesin | Collagen binding | ↓ Downregulated  (3.75-fold) | RT-qPCR | *E. faecalis* | No data | Kojic acid-aPDI (32 μg/mL, 450 nm, 150 mW/cm^2^, 1 min) | No data | [74] |
|  |  | ↓ Downregulated  (5.32-fold) | RT-qPCR |  |  | Parietin-aPDI (8 μg/mL, 450 nm, 150 mW/cm^2^, 1 min) | No data |  |
|  |  | ↓ Downregulated  (2-6-fold) | RT-qPCR |  | ATCC 29212 | Rutin-Ga(III)-aPDI (25 μmol/L, blue light, 300–420 J/cm^2^) | >3 log_10_ | [75] |
|  |  | Inconclusive | RT-qPCR |  | ATTC 29212  A1, A2 – clinical isolates, Karolinska Hospital culture collections  UmID1, UmID2, UmID3 - infected dental root-canals, Umeå bacterial collection | TMPyP-aPDI (0.015 µM, 445 nm, 210 J/cm^2^) | 1-3 log_10_ | [77] |
| *agrA* / accessory gene regulator A | Response regulator, quorum sensing | ↓ Downregulated  (3.7-fold) | RT-qPCR | *S. aureus* | ATCC 25923, Iranian Biological Resource Center | ICG-aPDI (31.2 µg/mL, 810 nm, 31.2 J/cm^2^) | No statistically significant reduction | [43] |
| *bcsA* / cellulose synthase catalytic subunit | Polymerizes uridine 5'-diphosphate glucose to cellulose | ↓ Downregulated  (approx. 4-fold) | RT-qPCR | *C. sakazakii* | ATCC 29004, American Type Culture Collection | 405 nm aBL (26 mW/cm^2^, 2h, 25°C) | ≤ 1 log_10_ | [59] |
| *bcsG* / cellulose biosynthesis protein | Cellulose biosynthesis | ↓ Downregulated  (approx. 4-fold) | RT-qPCR | *C. sakazakii* | ATCC 29004, American Type Culture Collection | 405 nm aBL (26 mW/cm^2^, 2h, 25°C) | ≤ 1 log_10_ | [59] |
| *bsmA* / biofilm peroxide resistance protein | Regulator of biofilm structure | ↓ Downregulated  (approx. 6- and 2.5-fold  for ATCC 13880)  (approx. 10-fold for  Sm2) | RT-qPCR | *S. marcescens* | ATCC 13880  Sm2 – clinical isolate from blood sample | MB-aPDI (25 and 50 μM, 660 nm, 15 J/cm^2^) | 1-3 log_10_ (sub-lethal)  >3 log_10_ (lethal) | [57] |
| *bsmB* / BsmB | Regulator of biofilm structure | ↓ Downregulated  (>10-fold for ATCC 13880)  (approx. 6-fold for Sm2) | RT-qPCR | *S. marcescens* | ATCC 13880  Sm2 – clinical isolate from blood sample | MB-aPDI (25 and 50 μM, 660 nm, 15 J/cm^2^) | 1-3 log_10_ (sub-lethal)  >3 log_10_ (lethal) | [57] |
| *cdrA* / two-partner secretion system adhesin | C-di-GMP signaling | ↓ Downregulated  (5.7-fold for 1 min)  ↑ Upregulated  (7.1-fold for 2 min)  (8.4-fold for 5 min) | RT-qPCR | *P. aeruginosa* | PAO1, reference strain | MB-aPDI (0.8 mM, 660 nm, 1,2, and 5 min) | < 1 log_10_ ( 1 min)  < 1 log_10_ ( 2 min)  1-3 log_10_ (5 min) | [25] |
| *cfa5* / coronafacic acid synthetase, ligase component | Biosynthesis of coronatine | ↓ Downregulated  (2-fold) | RT-qPCR | *P. syringae* | DC3000, London Research and Development Centre | 458 nm blue light (10 min, 20 μE/m^2^s) | No data | [83] |
| *cma* / coronamic acid synthetase | Biosynthesis of coronatine | ↓ Downregulated  (2-fold) | RT-qPCR | *P. syringae* | DC3000, London Research and Development Centre | 458 nm blue light (10 min, 20 μE/m^2^s) | No data | [83] |
| *comA* / ATP-binding protein | ABC transporter | ↓ Downregulated  (4.1-fold at 64 μg/mL)  (3.2-fold at 32 μg/mL) | RT-qPCR | *S. mutans* | ATCC 35668 | N-QCT-aPDI (64 μg/mL, 32 μg/mL 405 nm, 150 mW/cm^2^ for 60 s) | 1-3 log_10_ | [64] |
| *comB* / accessory factor for ComA | ABC transporter | ↓ Downregulated  (5.05-fold at 64 μg/mL)  (4.08-fold at 32 μg/mL) | RT-qPCR | *S. mutans* | ATCC 35668 | N-QCT-aPDI (64 μg/mL, 32 μg/mL 405 nm, 150 mW/cm^2^ for 60 s) | 1-3 log_10_ | [64] |
| *comDE* / histidine kinase of the competence regulon and competence protein | Two-component regulatory system | ↓ Downregulated  (4-fold at 64 μg/mL)  (3.56-fold at 32 μg/mL) | RT-qPCR | *S. mutans* | ATCC 35668 | N-QCT-aPDI (64 μg/mL, 32 μg/mL 405 nm, 150 mW/cm^2^ for 60 s) | 1-3 log_10_ | [64] |
|  |  | ↑ Upregulated  (approx. 1.3-fold) | RT-qPCR |  | MTCC 497, Institute of Microbial Technology, Chandigarh | TBO-aPDI (20 μg ml^−1^, 630 nm, 9.1 J cm^−2^) | 1-3 log_10_ | [65] |
|  |  | ↓ Downregulated  (approx. 1.4-fold) | RT-qPCR |  |  | TBO-AgNP-aPDI (10 μg ml^−1^, 630 nm, 9.1 J cm^−2^) | > 3 log_10_ |  |
| *csgA* / major curlin subunit | Encodes curlin - the major subunit protein of curli | ↓ Downregulated  (≥10⁴-fold; near  detection limit) | RT-qPCR | *E.coli* | CCTCC M2015233, obtained from MG1655 | 450 nm aBL (1300 lx) | < 1 log_10_ | [58] |
| *csgB* / minor curlin subunit | Nucleator, trigger the polymerization of CsgA to form curli fibers on the cell surface | ↓ Downregulated  (≥10⁴-fold; near  detection limit) | RT-qPCR | *E.coli* | CCTCC M2015233, obtained from MG1655 | 450 nm aBL (1300 lx) | < 1 log_10_ | [58] |
| *csgC* / curlin chaperone | Maintains the curlin subunit, CsgA, in a soluble monomeric state | ↓ Downregulated  (≥10⁴-fold; near  detection limit) | RT-qPCR | *E.coli* | CCTCC M2015233, obtained from MG1655 | 450 nm aBL (1300 lx) | < 1 log_10_ | [58] |
| *csgD* / DNA-binding transcriptional dual regulator | Transcriptional regulator that regulates several genes involved in the Curli assembly, transport, and structural components | ↓ Downregulated  (≥10³–10⁴-fold; near  detection limit) | RT-qPCR | *E.coli* | CCTCC M2015233, obtained from MG1655 | 450 nm aBL (1300 lx) | < 1 log_10_ | [58] |
| *csuAB* / Csu fimbrial major subunit | Biosynthesis of pili, biofilm formation | ↓ Downregulated  (approx. 1.45-fold) | RT-qPCR | *A. baumannii* | ATCC 17978, clinical isolate, human infant | 469 nm blue light | No data | [47] |
| *csuE /* Csu fimbrial tip adhesin | Adhesions involved in biofilm formation | ↓ Downregulated  (34-fold for CR-XDR-  AB)  (12-fold for ATCC 19606) | RT-qPCR | *A. baumannii* | CR-XDR-AB –  burn-wound infection  ATCC 19606 – reference strain | TBO-aPDI (0.37 mg/mL, 630nm, 180 J/cm^2^) | No reduction (growth inhibition) | [45] |
| *efa* / Enterococcus faecalis antigen A | Metal ion binding | ↓ Downregulated  (2.1-fold) | RT-qPCR | *E. faecalis* | ATCC 29212, Iranian Biological Resource Center | ICG-aPDI (1000 μg/mL, 810 nm, 31.2 J/cm^2^) | < 1 log_10_ | [73] |
|  |  | ↓ Downregulated  (approx. 18-fold) | RT-qPCR |  | ATCC 29212 | rGO-Cur-aPDI (125 μg/mL, 450 nm, 360 J/cm^2^) | biofilm culture, no reduction (growth inhibition) | [72] |
| *epsA* / EpsA | Facilitate capsule expression and biofilm development | ↓ Downregulated  (17.6-fold for CR-XDR-  AB)  (11.4-fold for ATCC  19606) | RT-qPCR | *A. baumannii* | CR-XDR-AB –  burn-wound infection  ATCC 19606 – reference strain | TBO-aPDI (0.37 mg/mL, 630nm, 180 J/cm^2^) | No reduction (growth inhibition) | [45] |
| *esp* / Enterococcal Surface Protein | Enhances bacterial adherence to surfaces and host tissues. | ↓ Downregulated  (4.4-, 6.2- and 6.0-fold) | RT-qPCR | *E. faecalis* | ATCC 29212, Iranian Biological  Resource Center | Fe-88-ICG-aPDI (810nm, 31.2 J/cm^2^)  Fe-101-ICG-aPDI (810nm, 31.2 J/cm^2^)  Al-101-ICG-aPDI (810nm, 31.2 J/cm^2^) | < 1 log_10_ | [70] |
|  |  | ↓ Downregulated  (3.2-fold) | RT-qPCR |  | ATCC 29212, Iranian Biological  Resource Center | TBO-aPDI (6.2 μg/mL, 635 nm, 103.12 J/cm^2^) | No data | [71] |
|  |  | ↓ Downregulated  (2.8-fold) | RT-qPCR |  |  | MB-aPDI (6.2 μg/mL, 660 nm, 70.31 J/cm^2^) | No data |  |
|  |  | ↓ Downregulated  (5.2-fold) | RT-qPCR |  |  | ICG-aPDI (31.2 μg/mL, 808 nm, 15.62 J/cm^2^) | No data |  |
|  |  | ↓ Downregulated  (approx. 23.0-fold) | RT-qPCR |  | ATCC 29212 | rGO-Cur-aPDI (125 μg/mL, 450 nm, 360 J/cm^2^) | biofilm culture, no reduction (growth inhibition) | [72] |
|  |  | ↓ Downregulated  (4.44-fold) | RT-qPCR |  | No data | Kojic acid-aPDI (32 μg/mL, 450 nm, 150 mW/cm^2^, 1 min) | No data | [74] |
|  |  | ↓ Downregulated  (6.06-fold) | RT-qPCR |  |  | Parietin-aPDI (8 μg/mL, 450 nm, 150 mW/cm2, 1 min) | No data |  |
|  |  | ↓ Downregulated  (4-8-fold) | RT-qPCR |  | ATCC 29212 | Rutin-Ga(III)-aPDI (25 μmol/L, blue light, 300–420 J/cm^2^) | >3 log_10_ | [75] |
| *fimA* / type-1 fimbrial protein subunit | Structural subunit of the major type-1 fimbriae, adhesion, and biofilm | ↓ Downregulated  (9.6-fold) | RT-qPCR | *P. gingivalis* | ATCC 33277 | GQD-Cur-aPDI (100 μg/mL, 435 nm, 60–80 J/cm^2^) | < 1 log_10_ | [50] |
|  |  | ↓ Downregulated  (14.4-fold) | RT-qPCR |  | Clinical isolates from root canal from patients treated with PAD | TBO-aPDI (6.25 μg/mL, 635 nm, 171.87 J/cm^2^) | No statistically significant reduction | [51] |
|  |  | ↓ Downregulated  (4.6-fold) | RT-qPCR |  |  | MB-aPDI (25 μg/mL, 660 nm, 93.75 J/cm^2^) | No statistically significant reduction |  |
|  |  | ↓ Downregulated  (17.3-fold) | RT-qPCR |  |  | ICG-aPDI (15.6 μg/mL, 810 nm, 15.6 J/cm^2^) | No statistically significant reduction |  |
|  |  | ↓ Downregulated  (>10-fold for Sm2) | RT-qPCR | *S. marcescens* | ATCC 13880  Sm2 – clinical isolate from blood sample | MB-aPDI (25 and 50 μM, 660 nm, 15 J/cm^2^) | 1-3 log_10_ (sub-lethal)  >3 log_10_ (lethal) | [57] |
|  |  | ↓ Downregulated  (approx. 256-fold) | RT-qPCR | *E. coli* | CCTCC M2015233, obtained from MG1655 | 450 nm aBL (1300 lx) | < 1 log_10_ | [58] |
| *fimA* / fimbria adhesin protein | Cell adhesion involved in single-species biofilm formation | ↓ Downregulated  (2-2.83-fold) | RNA-seq | *A. baumannii* | ATCC 19606 | 462 nm blue light (6-10 µmol photons x m^-2^ x s^-1^) | No data | [49] |
| *fimC* / P pilus assembly protein chaperone | Chaperone-mediated protein folding | ↓ Downregulated  (2-2.83-fold) | RNA-seq | *A. baumannii* | ATCC 19606 | 462 nm blue light (6-10 µmol photons x m^-2^ x s^-1^) | No data | [49] |
|  |  | ↓ Downregulated  (approx. 3.0- and 6.0-  fold for Sm2) | RT-qPCR | *S. marcescens* | ATCC 13880  Sm2 – clinical isolate from blood sample | MB-aPDI (25 and 50 μM, 660 nm, 15 J/cm^2^) | 1-3 log_10_ (sub-lethal)  >3 log_10_ (lethal) | [57] |
| *fimD* / outer membrane usher protein | Export and assembly of type I fimbriae | ↓ Downregulated  (2-2.83-fold) | RNA-seq | *A. baumannii* | ATCC 19606 | 462 nm blue light (6-10 µmol photons x m^-2^ x s^-1^) | No data | [49] |
| *fimH* / type 1 fimbriae D-mannose specific adhesin | Mediate binding to receptor structures allowing the bacteria to colonize various host tissues | ↓ Downregulated  (approx. 2048-fold) | RT-qPCR | *E.coli* | CCTCC M2015233, obtained from MG1655 | 450 nm aBL (1300 lx) | < 1 log_10_ | [58] |
| *flgJ* / peptidoglycan hydrolase | Flagellum-specific muramidase which hydrolyzes the peptidoglycan | ↓ Downregulated  (approx. 4.0-fold) | RT-qPCR | *C. sakazakii* | ATCC 29004, American Type Culture Collection | 405 nm aBL (26 mW/cm^2^, 2h, 25°C) | ≤ 1 log_10_ | [59] |
|  |  | ↑ Upregulated  (approx. 3.5-fold) | RT-qPCR |  | ATCC 29544, Beijing BeNa Culture Collection | Hypocrellin B-aPDI (30 μM, 460 nm, 20 J/cm^2^) | 1-3 log_10_ | [82] |
| *flgK* / flagellar hook-associated protein 1 | Connects the flagellar hook to the flagellar filament | ↑ Upregulated  (approx. 2.5-fold) | RT-qPCR | *C. sakazakii* | ATCC 29544, Beijing BeNa Culture Collection | Hypocrellin B-aPDI (30 μM, 460 nm, 20 J/cm^2^) | 1-3 log_10_ | [82] |
| *flhC* / DNA-binding transcriptional dual regulator | DNA binding and regulation of transcription | ↓ Downregulated  (approx. 32768-fold) | RT-qPCR | *E. coli* | CCTCC M2015233, obtained from MG1655 | 450 nm aBL (1300 lx) | < 1 log_10_ | [58] |
| *flhD* / flagellar transcriptional regulator | Master transcriptional regulator of several flagellar and non-flagellar operons | ↓ Downregulated  (approx. 4.0- and 10.0-fold for ATCC 13880)  (approx. 10-fold for Sm2) | RT-qPCR | *S. marcescens* | ATCC 13880  Sm2 – clinical isolate from blood sample | MB-aPDI (25 and 50 μM, 660 nm, 15 J/cm^2^) | 1-3 log_10_ (sub-lethal)  >3 log_10_ (lethal) | [57] |
|  |  | ↓ Downregulated  (approx. 1048576-fold) | RT-qPCR | *E.coli* | CCTCC M2015233, obtained from MG1655 | 450 nm aBL (1300 lx) | < 1 log_10_ | [58] |
|  |  | ↓ Downregulated  (approx. 4.0-fold) | RT-qPCR | *C. sakazakii* | ATCC 29004, American Type Culture Collection | 405 nm aBL (26 mW/cm^2^, 2h, 25°C) | ≤ 1 log_10_ | [59] |
| *fliA* / RNA polymerase sigma factor | Minor sigma factor that is responsible for initiation of transcription | ↓ Downregulated  (approx. 1048576-fold) | RT-qPCR | *E. coli* | CCTCC M2015233, obtained from MG1655 | 450 nm aBL (1300 lx) | < 1 log_10_ | [58] |
| *fliC* / flagellin | Basic subunit that polymerizes to form the rigid flagellar filament | ↓ Downregulated  (approx. 2048-fold) | RT-qPCR | *E. coli* | CCTCC M2015233, obtained from MG1655 | 450 nm aBL (1300 lx) | < 1 log_10_ | [58] |
|  |  | ↑ Upregulated  (approx. 3.75-fold) | RT-qPCR | *C. sakazakii* | ATCC 29544, Beijing BeNa Culture Collection | Hypocrellin B-aPDI (30 μM, 460 nm, 20 J/cm^2^) | 1-3 log_10_ | [82] |
|  |  | ↓ Downregulated  (2.0-fold) | RT-qPCR | *P. syringae* | DC3000, London Research and Development Centre | 458 nm blue light (10 min, 20 μE/m^2^s) | No data | [83] |
| *fliD* / flagellar cap protein | Stabilizes the flagellar structure and facilitates the polymerization of flagellin | ↓ Downregulated  (approx. 8.0-fold) | RT-qPCR | *C. sakazakii* | ATCC 29004, American Type Culture Collection | 405 nm aBL (26 mW/cm^2^, 2h, 25°C) | ≤ 1 log_10_ | [59] |
| *fliH* / flagellar assembly protein | Export and assembly of flagellar components | ↑ Upregulated  (approx. 4.5-fold) | RT-qPCR | *C. sakazakii* | ATCC 29544, Beijing BeNa Culture Collection | hypocrellin B-aPDI (30 μM, 460 nm, 20 J/cm^2^) | 1-3 log_10_ | [82] |
| *fliK* / flagellar hook length control protein | Regulates the hook length during flagellum assembly | ↑ Upregulated  (approx. 5.0-fold) | RT-qPCR | *C. sakazakii* | ATCC 29544, Beijing BeNa Culture Collection | hypocrellin B-aPDI (30 μM, 460 nm, 20 J/cm^2^) | 1-3 log_10_ | [82] |
| *fsrB* / FsrB | Putative role in processing signaling molecules, QS system | ↓ Downregulated  (10.8-fold) | RT-qPCR | *E. faecalis* | ATCC 29212, Iranian Biological Resource Center | C-PC-aPDI (125 μg/mL, 635 nm, 34.73 J/cm^2^) | < 1 log_10_ | [76] |
| *fsrC* / transmembrane histidine kinase FsrC | Role in senses cell density and activates the response regulator of the regulon; FsrA | ↓ Downregulated  (4.1-fold) | RT-qPCR | *E. faecalis* | ATCC 29212, Iranian Biological Resource Center | ICG-aPDI (1000 μg/mL, 810 nm, 31.2 J/cm^2^) | < 1 log_10_ | [73] |
|  |  | ↓ Downregulated  (approx. 8.0-fold) | RT-qPCR |  | ATCC 29212 | rGO-Cur-aPDI (125 μg/mL, 450 nm, 360 J/cm^2^) | biofilm culture, no reduction (growth inhibition) | [72] |
|  |  | ↑↓ Variable – strain  dependent | RT-qPCR |  | ATTC 29212  A1, A2 – clinical isolates, Karolinska Hospital culture collections  UmID1, UmID2, UmID3 - infected dental root-canals, Umeå bacterial collection | TMPyP-aPDI (0.015 µM, 445 nm, 210 J/cm^2^) | 1-3 log_10_ | [77] |
| *ftf* / levansucrase | Catalyzes the synthesis of levan, a fructose polymer | ↑ Upregulated  (approx. 1.3-fold) | RT-qPCR | *S. mutans* | MTCC 497, Institute of Microbial Technology, Chandigarh | TBO-aPDI (20 μg ml^−1^, 630 nm, 9.1 J cm^−2^) | 1-3 log_10_ | [65] |
|  |  | ↓ Downregulated  (approx. 1.5-fold) | RT-qPCR |  |  | TBO-AgNP-aPDI (10 μg ml^−1^, 630 nm, 9.1 J cm^−2^) | > 3 log_10_ |  |
| *gbpB* / glucan-binding protein B | Unknown | ↑ Upregulated  (approx. 1.7-fold) | RT-qPCR | *S. mutans* | MTCC 497, Institute of Microbial Technology, Chandigarh | TBO-aPDI (20 μg ml^−1^, 630 nm, 9.1 J cm^−2^) | 1-3 log_10_ | [65] |
|  |  | ↓ Downregulated  (approx. 2.5- and 1.25-  fold) | RT-qPCR |  |  | TBO-AgNP-aPDI (10 μg ml^−1^, 630 nm, 9.1 J cm^−2^) | > 3 log_10_ |  |
| *gelE* / gelatinase | Metalloprotease capable of the hydrolysis of Insoluble hydrophobic substrates | ↓ Downregulated  (3.5-fold) | RT-qPCR | *E. faecalis* | ATCC 29212, Iranian Biological Resource Center | ICG-aPDI (1000 μg/mL, 810 nm, 31.2 J/cm^2^) | < 1 log_10_ | [73] |
|  |  | ↓ Downregulated  (approx. 8.5-fold) | RT-qPCR |  | ATCC 29212 | rGO-Cur-aPDI (125 μg/mL, 450 nm, 360 J/cm^2^) | biofilm culture, no reduction (growth inhibition) | [72] |
|  |  | ↓ Downregulated  (3-6-fold) | RT-qPCR |  | ATCC 29212 | Rutin-Ga(III)-aPDI (25 μmol/L, blue light, 300–420 J/cm^2^) | >3 log_10_ | [75] |
|  |  | ↑↓ Variable – strain  dependent | RT-qPCR |  | ATTC 29212  A1, A2 – clinical isolates, Karolinska Hospital culture collections  UmID1, UmID2, UmID3 - infected dental root-canals, Umeå bacterial collection | TMPyP-aPDI (0.015 µM, 445 nm, 210 J/cm^2^) | 1-3 log_10_ | [77] |
| *gtfB* / glucosyltransferase-I | Production of extracellular glucans | ↓ Downregulated  (3.9-fold) | RT-qPCR | *S. mutans* | Clinical isolates from dental plaque | TBO-aPDI (23.12 µM/mL, 635 nm, 68.75 J/cm^2^) | No statistically significant reduction | [60] |
|  |  | ↓ Downregulated  (8.25-fold) | RT-qPCR |  |  | ICG-aPDI (20.15 µM/mL, 810 nm, 31.2 J/cm^2^) | No statistically significant reduction |  |
|  |  | ↓ Downregulated  (5.1-fold) | RT-qPCR |  | ATCC 35668, Iranian Biological Resource Center | Rib-aPDI (64 µg/mL, 450nm, 60–80 J/cm^2^) | Biofilm culture,  < 1 log_10_ | [61] |
|  |  | ↓ Downregulated  (7.8-fold) | RT-qPCR |  | ATCC 35668, Iranian Biological Resource Center | Emo-CS-NPs-aPDI (0.58 µg/mL Emo/ 3.1 µg/mL CS, 405 nm, 313.7 J/cm^2^) | Biofilm culture,  < 1 log_10_ | [62] |
|  |  | ↓ Downregulated  (4.1-fold) | RT-qPCR |  | ATCC 35668, Iranian Biological Resource Center | Ulva lactuca-aPDI (0.5%, 450 nm, 150 mW/cm^2^) | Biofilm culture,  < 1 log_10_ | [63] |
|  |  | ↓ Downregulated  (6.5-fold at 64 μg/mL)  (4.1-fold at 32 μg/mL) | RT-qPCR |  | ATCC 35668 | N-QCT-aPDI (64 μg/mL, 32 μg/mL 405 nm, 150 mW/cm^2^ for 60 s) | 1-3 log_10_ | [64] |
|  |  | ↓ Downregulated  T2 (approx. 2.5-fold)  T3 (approx. 5.0-fold)  T4 (approx. 8.0-fold)  T5 (approx. 15.0-fold) | RT-qPCR |  | Isolates from teeth from patients treated with NMCur-aPDI | NMCur-aPDI (80 mg, 450 nm, 14 J/cm^2^, days 0 [T1], 30 [T2], 60 [T3], 90 [T4], and 120 [T5]) | < 1 log_10_ [T2]  1-3 log_10_ [T3]  1-3 log_10_ [T4]  > 3 log_10_ [T5] | [66] |
|  |  | ↓ Downregulated  (3.5-fold) | RT-qPCR |  | ATCC 35668, Iranian Biological Resource Center | PhotoActive+-aPDI (2.4×10-3 mol/L, 635 nm, 104 J/cm^2^) | No statistically significant reduction | [67] |
| *gtfC* / glucosyltransferase-SI | Production of extracellular glucans | ↓ Downregulated  (5.3-fold) | RT-qPCR | *S. mutans* | ATCC 35668, Iranian Biological Resource Center | Ulva lactuca-aPDI (0.5%, 450 nm, 150 mW/cm^2^) | Biofilm culture,  < 1 log_10_ | [63] |
|  |  | ↑ Slightly Upregulated  (1.2-fold) | RT-qPCR |  | MTCC 497, Institute of Microbial Technology, Chandigarh | TBO-aPDI (20 μg ml^−1^, 630 nm, 9.1 J cm^−2^) | 1-3 log_10_ | [65] |
|  |  | ↓ Downregulated  (approx. 1.7-fold) | RT-qPCR |  |  | TBO-AgNP-aPDI (10 μg ml^−1^, 630 nm, 9.1 J cm^−2^) | > 3 log_10_ |  |
| *gtfD* / glucosyltransferase-S | Production of extracellular glucans | ↓ Downregulated  (7.4-fold) | RT-qPCR | *S. mutans* | ATCC 35668, Iranian Biological Resource Center | Ulva lactuca-aPDI (0.5%, 450 nm, 150 mW/cm^2^) | Biofilm culture,  < 1 log_10_ | [63] |
| *icaA* / poly-beta-1,6-N-acetyl-D-glucosamine synthase | Catalyzes formation of  biofilm adhesin polysaccharide | ↓ Downregulated  (12.5-fold, average for  MRSA, MSSA and  ATCC) | RT-qPCR | *S. aureus* | ATTC 25923, MRSA and MSSA isolates from patients with burn wound infection | TBO-aPDI (25 µg/mL, 630 nm, 2000 mW/cm^2^, 104.1 J/cm^2^) | No statistically significant reduction | [68] |
| *icaB* / poly-beta-1,6-N-acetyl-D-glucosamine N-deacetylase | Catalyzes N-deacetylation of biofilm adhesin polysaccharide | ↓ Downregulated  (14-fold, average for  MRSA, MSSA and  ATCC) | RT-qPCR | *S. aureus* | ATTC 25923, MRSA and MSSA isolates from patients with burn wound infection | TBO-aPDI (25 µg/mL, 630 nm, 2000 mW/cm^2^, 104.1 J/cm^2^) | No statistically significant reduction | [68] |
| *icaC* / putative poly-beta-1,6-N-acetyl-D-glucosamine export protein | Presumably involved in the export of the biofilm adhesin across the cell membrane | ↓ Downregulated  (11.5-fold, average for  MRSA, MSSA and  ATCC) | RT-qPCR | *S. aureus* | ATTC 25923, MRSA and MSSA isolates from patients with burn wound infection | TBO-aPDI (25 µg/mL, 630 nm, 2000 mW/cm^2^, 104.1 J/cm^2^) | No statistically significant reduction | [68] |
| *icaD* / poly-beta-1,6-N-acetyl-D-glucosamine synthesis protein | Necessary for the synthesis of biofilm adhesin polysaccharide | ↓ Downregulated  (9.0-fold, average for  MRSA, MSSA and  ATCC) | RT-qPCR | *S. aureus* | ATTC 25923, MRSA and MSSA isolates from patients with burn wound infection | TBO-aPDI (25 µg/mL, 630 nm, 2000 mW/cm^2^, 104.1 J/cm^2^) | No statistically significant reduction | [68] |
| *icaR* / ica operon transcriptional regulator IcaR | Represses transcription of the icaADBC operon | ↓ Downregulated  (7.0-fold, average for  MRSA, MSSA and  ATCC) | RT-qPCR | *S. aureus* | ATTC 25923, MRSA and MSSA isolates from patients with burn wound infection | TBO-aPDI (25 µg/mL, 630 nm, 2000 mW/cm^2^, 104.1 J/cm^2^) | No statistically significant reduction | [68] |
| *inpA (pin0048)* / protease interpain A | Cysteine protease | ↓ Downregulated  (11.8-fold) | RT-qPCR | *P. intermedia* | ATCC 49046 | GQD-Cur-aPDI (100 μg/mL, 435 nm, 60–80 J/cm^2^) | < 1 log_10_ | [50] |
| *lasB* / elastase LasB | Cleaves host elastin, collagen, IgG | ↓ Downregulated  (approx. 4.0- and 10-fold) | RT-qPCR | *P. aeruginosa* | PAO1, reference strain | ALA-aPDI (10, 20 mM, 630 nm, 108 J/cm^2^ ) | < 1 log_10_ | [39] |
|  |  | ↑ Upregulated  (4.5-fold for 1 min)  (5.1-fold for 2 min)  (7.9-fold for 5 min) | RT-qPCR |  | PAO1, reference strain | MB-aPDI (0.8 mM, 660 nm, 1,2 and 5 min) | < 1 log_10_ ( 1 min)  < 1 log_10_ ( 2 min)  1-3 log_10_ (5 min) | [25] |
| *lasI* / acyl-homoserine-lactone synthase | Signal transduction, quorum sensing | ↓ Downregulated  (approx. 4- and 10-fold) | RT-qPCR | *P. aeruginosa* | PAO1, reference strain | ALA-aPDI (10, 20 mM, 630 nm, 108 J/cm^2^) | < 1 log_10_ | [39] |
|  |  | ↓ Downregulated  (> 2-fold) | RT-qPCR |  | ATCC 27853 | MB-aPDI (0.012 mM, 650 nm, 23 J/cm^2^) | 1-3 log_10_ | [40] |
|  |  | ↓ Downregulated  (< 1.4-fold for all  isolates) | RT-qPCR |  | ATCC 27853  P1-P6 – clinical isolates from burn wound infections | MB-aPDI (0.012 mM, 650 nm, 23 J/cm^2^) | 1-3 log_10_ | [41] |
|  |  | ↓ Downregulated  (4.9-fold) | RT-qPCR |  | ATCC 27853, Iranian Biological Resource Center | ICG-aPDI (31.2 µg/mL, 810 nm, 31.2 J/cm^2^) | No statistically significant reduction | [43] |
| *lasR* / transcriptional activator protein LasR | Quorum sensing, transcriptional regulator of genes | ↓ Downregulated  (approx. 2.5- and 5.0-fold) | RT-qPCR | *P. aeruginosa* | PAO1, reference strain | ALA-aPDI (10, 20 mM, 630 nm, 108 J/cm^2^) | < 1 log_10_ | [39] |
|  |  | ↓ Downregulated  (> 10-fold) | RT-qPCR |  | ATCC 27853 | MB-aPDI (0.012 mM, 650 nm, 23 J/cm^2^) | 1-3 log_10_ | [40] |
|  |  | ↓ Downregulated  (< 1.4-fold for all  isolates) | RT-qPCR |  | ATCC 27853  P1-P6 – clinical isolates from burn wound infections | MB-aPDI (0.012 mM, 650 nm, 23 J/cm^2^) | 1-3 log_10_ | [41] |
| *lrgA* / antiholin-like protein | Modulation of protein transport across the plasma membrane | ↓ Downregulated  (approx. 5-fold) | RNA-seq and RT-qPCR | *S. aureus* | ATCC 25923, BeNa Culture Collection | 460 nm aBL (50 mW/cm^2^, 30 J/cm^2^) | < 1 log_10_ | [69] |
|  |  | ↑ Upregulated  (approx. 1.45-fold) |  |  |  | Hypocrellin B-aPDI (500 nM, 460 nm, 9 J/cm^2^) | 1-3 log_10_ |  |
| *luxR* / LuxR C-terminal-related transcriptional regulator | Transcriptional activator of the bioluminescence operon | ↓ Downregulated  (approx. 4-fold) | RT-qPCR | *C. sakazakii* | ATCC 29004, American Type Culture Collection | 405 nm aBL (26 mW/cm^2^, 2h, 25°C) | < 1 log_10_ | [59] |
| *motA* / motility protein A | Form flagellar motor | ↓ Downregulated  (approx. 32768-fold) | RT-qPCR | *E. coli* | CCTCC M2015233, obtained from MG1655 | 450 nm aBL (1300 lx) | < 1 log_10_ | [58] |
|  |  | ↓ Downregulated  (approx. 4-fold) | RT-qPCR | *C. sakazakii* | ATCC 29004, American Type Culture Collection | 405 nm aBL (26 mW/cm^2^, 2h, 25°C) | < 1 log_10_ | [59] |
| *motB* / motility protein B | Form flagellar motor | ↓ Downregulated  (approx. 4-fold) | RT-qPCR | *C. sakazakii* | ATCC 29004, American Type Culture Collection | 405 nm aBL (26 mW/cm^2^, 2h, 25°C) | < 1 log_10_ | [59] |
| *pelA* / PelA | Putative - Pel polysaccharide production | ↓ Downregulated  (> 50-fold CDsGEN-  NH2;  (approx. 14-fold CDs-  IMP- NH2)  ( > 100-fold CDs-IMP-  GEN)  (approx. 100-fold CDs-  GEN-IMP) | RT-qPCR | *P. aeruginosa* | ATCC 27853, and  clinical isolate from burn wounds | CDsGEN-NH2-aPDI (6 mg/mL, 320 nm, 12.5 J/cm^2^)  CDs-IMP-NH2-aPDI (12 mg/mL, 320 nm, 12.5 J/cm^2^)  CDs-IMP-GEN-aPDI (1 mg/mL, 320 nm, 12.5 J/cm^2^)  CDs-GEN-IMP-aPDI (12 mg/mL, 320 nm, 12.5 J/cm^2^) | Biofilm cultures,  > 3 log_10_ for both strains and all treatments | [42] |
| *pelF* / biofilm biosynthesis glycosyltransferase PelF | Pel polysaccharide production | ↓ Downregulated  (> 10-fold) | RT-qPCR | *P. aeruginosa* | ATCC 27853 | MB-aPDI (0.012 mM, 650 nm, 23 J/cm^2^) | 1-3 log_10_ | [40] |
| *phzB2* / phenazine biosynthesis protein | Synthesis of phenazine-1-carboxylic acid (PCA) | No significant change | RT-qPCR | *P. aeruginosa* | PA14 – reference strain  133/k – urine clinical isolate | GaCHP-aPDI (5,10 μM, 409 nm, 2.4 J/cm^2^) | < 1 log_10_ | [88] |
| *phzH* / phenazine-modifying protein | Phenazine biosynthesis | ↓ Downregulated  (approx. 2- and >10-fold) | RT-qPCR | *P. aeruginosa* | PAO1, reference strain | ALA-aPDI (10, 20 mM, 630 nm, 108 J/cm^2^) | < 1 log_10_ | [39] |
| *phzM* / phenazine-1-carboxylate N-methyltransferase | Pyocyanine biosynthesis | ↑ Upregulated  (approx. 1024-fold for  ATCC 27853, P2 and P3) | RT-qPCR | *P. aeruginosa* | ATCC 27853  P1-P6 – clinical isolates from burn wound infections | MB-aPDI (0.012 mM, 650 nm, 23 J/cm^2^) | 1-3 log_10_ | [41] |
|  |  | No significant change | RT-qPCR |  | PA14 – reference strain  133/k – urine clinical isolate | GaCHP-aPDI (5,10 μM, 409 nm, 2.4 J/cm^2^) | < 1 log_10_ | [88] |
| *phzS* / 5-methylphenazine-1-carboxylate 1-monooxygenase | Biosynthesis of pyocyanin,  catalyzes the oxidative decarboxylation of (5-methyl-PCA) to pyocyanine. | No significant change | RT-qPCR | *P. aeruginosa* | PA14 – reference strain  133/k – urine clinical isolate | GaCHP-aPDI (5,10 μM, 409 nm, 2.4 J/cm^2^) | < 1 log_10_ | [88] |
| *pilZ* / type 4 fimbrial biogenesis protein | C-di-GMP binding | No significant change | RT-qPCR | *A. baumannii* | XDR isolate from burn wound infection | TBO-aPDI (0.01 mg/mL, 630 nm, 2000-4000 mW/cm^2^, 60 s) | No data | [81] |
| *pqsA* / anthranilate--CoA ligase | PQS signaling | No significant change | RT-qPCR | *P. aeruginosa* | PAO1, reference strain | MB-aPDI (0.8 mM, 660 nm, 1,2 and 5 min) | < 1 log_10_ ( 1 min)  < 1 log_10_ ( 2 min)  1-3 log_10_ (5 min) | [25] |
| *prpABCD operon* / predicted type I pilus | Unknown | ↓ Downregulated  *prpA* (2.75-fold)  *prpB* (4.41-fold)  *prpC* (3.08-fold)  *prpD* (5.76-fold) | RNA-seq | *A. baumannii* | ATCC 17978, clinical isolate | 462 nm blue light | No data | [48] |
|  |  | ↓ Downregulated  *prpA* at 24°C (2.2-  fold) | RT-qPCR |  |  |  |  |  |
| *pslA* / biofilm formation protein PslA | Psl polysaccharide production | ↓ Downregulated  (> 10-fold) | RT-qPCR | *P. aeruginosa* | ATCC 27853 | MB-aPDI (0.012 mM, 650 nm, 23 J/cm^2^) | 1-3 log_10_ | [40] |
|  |  | ↓ Downregulated  (approx. 100-fold  CDsGEN-NH2)  (approx. 20-fold CDs-  IMP- NH2)  (> 100-fold CDs-IMP-  GEN)  (approx. 100-fold CDs-  GEN-IMP) | RT-qPCR |  | ATCC 27853, and  clinical isolate from burn wounds | CDsGEN-NH2-aPDI (6 mg/mL, 320 nm, 12.5 J/cm^2^)  CDs-IMP-NH2-aPDI (12 mg/mL, 320 nm, 12.5 J/cm^2^)  CDs-IMP-GEN-aPDI (1 mg/mL, 320 nm, 12.5 J/cm^2^)  CDs-GEN-IMP-aPDI (12 mg/mL, 320 nm, 12.5 J/cm^2^) | Biofilm cultures,  > 3 log_10_ for both strains and all treatments | [42] |
| *qseB* / transcriptional regulatory protein | Two-component regulatory system | ↓ Downregulated  (2.5-fold - 10×10^–4^ g/L)  (4.9-fold - 25×10^–4^ g/L)  (6.3-fold - 50×10^–4^ g/L) | RT-qPCR | *A. actinomycetemcomitans* | IR-TUMS/BPG4, Genbank: KX108928 | Cur-NPhs-aPDI (10, 25, 50 × 10^–4^ g/L, 450 nm, 2 min) | > 3 log_10_ for all treatment conditions | [53] |
| *qseC* / quorum sensing histidine kinase | Two-component regulatory system | ↓ Downregulated  (3.1-fold - 10×10^–4^ g/L)  (5.4-fold - 25x10^–4^ g/L)  (7.8-fold - 50×10^–4^ g/L) | RT-qPCR | *A. actinomycetemcomitans* | IR-TUMS/BPG4, Genbank: KX108928 | Cur-NPhs-aPDI (10, 25, 50 × 10^–4^ g/L, 450 nm, 2 min) | > 3 log_10_ for all treatment conditions | [53] |
| *rcpA* / RcpA | Pili assembly and adherence | ↓ Downregulated  (8.1-fold) | RT-qPCR | *A. actinomycetemcomitans* | ATCC 33384 | GQD-Cur-aPDI (100 μg/mL, 435 nm, 60–80 J/cm^2^) | < 1 log_10_ | [50] |
|  |  | ↓ Downregulated  (8.5-fold) | RT-qPCR |  | ATCC 33384, Institute of Microbiology, ETH Zurich | CUR-aPDI (1.2 µmol/mL, 450 nm, 180- 240 J/cm^2^) | No statistically significant reduction | [52] |
|  |  | ↓ Downregulated  (4.4-fold - 10×10^–4^ g/L)  (5.2-fold - 25x10^–4^ g/L)  (9.7-fold - 50×10^–4^ g/L) | RT-qPCR |  | IR-TUMS/BPG4, Genbank: KX108928 | Cur-NPhs-aPDI (10, 25, 50 × 10^–4^ g/L, 450 nm, 2 min) | > 3 log_10_ for all treatment conditions | [53] |
|  |  | ↓ Downregulated  (3.83-fold) | RT-qPCR |  | ATCC 33384 | MB-aPDI (25 µg/mL, 660 nm, 93.75 J/cm^2^) | < 1 log_10_ | [54] |
|  |  | ↓ Downregulated  (5.65-fold) | RT-qPCR |  | ATCC 33384 | ICG-aPDI (125 μg/mL, 808 nm, 15.6 J/cm^2^) | < 1 log_10_ | [55] |
|  |  | ↓ Downregulated  (6.4-fold) | RT-qPCR |  | ATCC 33384 | ICG-aPDI (810 nm, 31.2 J/cm^2^) | No data | [56] |
|  |  | ↓ Downregulated  (13.2-fold) | RT-qPCR |  |  | CS-NPs@ICG-aPDI (810 nm, 31.2 J/cm^2^) | No data |  |
| *rhlA* / 3-(3-hydroxydecanoyloxy) decanoate synthase | Rhamnolipid surfactant production | ↓ Downregulated  (< 1-fold for all  isolates) | RT-qPCR | *P. aeruginosa* | ATCC 27853  P1-P6 – clinical isolates from burn wound infections | MB-aPDI (0.012 mM, 650 nm, 23 J/cm^2^) | 1-3 log_10_ | [41] |
|  |  | No significant change | RT-qPCR |  | PAO1, reference strain | MB-aPDI (0.8 mM, 660 nm, 1,2 and 5 min) | < 1 log_10_ ( 1 min)  < 1 log_10_ ( 2 min)  1-3 log_10_ (5 min) | [25] |
| *rhlI* / acyl-homoserine-lactone synthase | Signal transduction, quorum sensing | ↓ Downregulated  (approx. 2.5- and 5.0-  fold) | RT-qPCR | *P. aeruginosa* | PAO1, reference strain | ALA-aPDI (10, 20 mM, 630 nm, 108 J/cm^2^) | < 1 log_10_ | [39] |
|  |  | ↓ Downregulated  (> 10-fold) | RT-qPCR |  | ATCC 27853 | MB-aPDI (0.012 mM, 650 nm, 23 J/cm^2^) | 1-3 log_10_ | [40] |
|  |  | ↓ Downregulated  (< 1.4-fold) | RT-qPCR |  | ATCC 27853  P1-P6 – clinical isolates from burn wound infections | MB-aPDI (0.012 mM, 650 nm, 23 J/cm^2^) | 1-3 log_10_ | [41] |
| *rhlR* / HTH-type quorum-sensing regulator RhlR | Quorum sensing, transcriptional regulator of genes | ↓ Downregulated  (approx. 2.5- and 5.0-  fold) | RT-qPCR | *P. aeruginosa* | PAO1, reference strain | ALA-aPDI (10, 20 mM, 630 nm, 108 J/cm^2^) | < 1 log_10_ | [39] |
|  |  | ↓ Downregulated  (> 100-fold) | RT-qPCR |  | ATCC 27853 | MB-aPDI (0.012 mM, 650 nm, 23 J/cm^2^) | 1-3 log_10_ | [40] |
|  |  | ↓ Downregulated  (< 1.4-fold) | RT-qPCR |  | ATCC 27853  P1-P6 – clinical isolates from burn wound infections | MB-aPDI (0.012 mM, 650 nm, 23 J/cm^2^) | 1-3 log_10_ | [41] |
| *seb* / staphylococcal enterotoxin B (SEB) | Superantigen | ↓ Downregulated  t1 (2.05-fold)  t2 (2.82-fold) | RT-qPCR | *S. aureus* | 140/05 – reference strain, National Medicines Institute | RB-aPDI (0.25 µM, 515 nm, 2 J/cm^2^, t1- 20 min, t2- 40 min) | < 1 log_10_ | [24] |
|  |  | ↓ Downregulated  t1 (1.15-fold)  t2 (1.87-fold) | RT-qPCR |  |  | NMB-aPDI (5 µM, 632 nm, 20 J/cm^2^, t1- 20 min, t2- 40 min) | < 1 log_10_ |  |
| *sec* / staphylococcal enterotoxin C (SEC) | Superantigen | ↓ Downregulated  (13-fold) | RT-qPCR | *S. aureus* | 5N – nasal isolate from atopic dermatitis patient | GaCHP-aPDI (1 μM, 409 nm, 6.24 J/cm^2^) | < 1 log_10_ | [88] |
|  |  | ↓ Downregulated  (6-fold) | RT-qPCR | *S. aureus* | 5N – nasal isolate from atopic dermatitis patient | GaCHP-aPDI (1 μM, 522 nm, 1.52 J/cm^2^) | < 1 log_10_ | [87] |
|  |  | ↓ Downregulated  (2.77-fold) | RT-qPCR |  |  | GaMPIX-aPDI (10 μM, 522 nm, 12.7 J/cm^2^) | < 1 log_10_ |  |
| *spaP* / surface protein adhesin | Bacterial adhesion and biofilm formation | ↑ Slightly Upregulated  (approx. 1.2-fold) | RT-qPCR | *S. mutans* | MTCC 497, Institute of Microbial Technology, Chandigarh | TBO-aPDI (20 μg ml^−1^, 630 nm, 9.1 J cm^−2^) | 1-3 log_10_ | [65] |
|  |  | ↓ Downregulated  (approx. 1.25-fold) | RT-qPCR |  |  | TBO-AgNP-aPDI (10 μg ml^−1^, 630 nm, 9.1 J cm^−2^) | > 3 log_10_ |  |
| *srrA* / transcriptional regulatory protein SrrA | Global virulence regulator | No significant change | RT-qPCR | *S. aureus* | 5N – nasal isolate from atopic dermatitis patient | GaCHP-aPDI (1 μM, 522 nm, 1.52 J/cm^2^) | < 1 log_10_ | [87] |
| *ssrB* / transcriptional regulatory protein SrrB | Global virulence regulator | ↑ Upregulated  (approx. 2-fold) | RT-qPCR | *S. aureus* | 5N – nasal isolate from atopic dermatitis patient | GaCHP-aPDI (1 μM, 522 nm, 1.52 J/cm^2^) | < 1 log_10_ | [87] |
| *swrR* / LuxR-type transcriptional regulator | QS system | ↓ Downregulated  (approx. 4-fold for ATCC 13880)  ↓ Downregulated  (> 10-fold for Sm2) | RT-qPCR | *S. marcescens* | ATCC 13880  Sm2 – clinical isolate from blood sample | MB-aPDI (25 and 50 μM, 660 nm, 15 J/cm^2^) | 1-3 log_10_ (sub-lethal)  >3 log_10_ (lethal) | [57] |
| *traI* / conjugative transfer relaxase | Plasmid conjugation and horizontal gene transfer | ↓ Downregulated  (approx. 3-fold) | RNA-seq and RT-qPCR | *A. baumannii* | ATCC 17978, clinical isolate, human infant | 469 nm blue light | No data | [47] |
| *tssC* / type VI secretion system contractile sheath large subunit | Structural component of the T6SS contractile sheath | ↑ Upregulated  (4-16-fold) | RT-qPCR | *A. baumannii* | ATCC 19606 | 462 nm blue light (6-10 µmol photons x m^-2^ x s^-1^) | No data | [49] |
| *tssD* / type VI secretion system tube protein | Secreted effector and structural component | ↑ Upregulated  (4-16-fold) | RT-qPCR | *A. baumannii* | ATCC 19606 | 462 nm blue light (6-10 µmol photons x m^-2^ x s^-1^) | No data | [49] |
| *tssM* / type VI secretion system membrane subunit | Membrane-associated protein that anchors the T6SS machinery | ↑ Upregulated  (4-16-fold) | RT-qPCR | *A. baumannii* | ATCC 19606 | 462 nm blue light (6-10 µmol photons x m^-2^ x s^-1^) | No data | [49] |
| *tst* / toxic shock syndrome toxin-1 (TSST-1) | Superantigen | No significant change | RT-qPCR | *S. aureus* | 5N – nasal isolate from atopic dermatitis patient | GaCHP-aPDI (1 μM, 409 nm, 6.24 J/cm^2^) | < 1 log_10_ | [88] |
|  |  | ↑ Upregulated  (2.83-fold) | RT-qPCR | *S. aureus* | 5N – nasal isolate from atopic dermatitis patient | GaCHP-aPDI (1 μM, 522 nm, 1.52 J/cm^2^) | < 1 log_10_ | [87] |
|  |  | No significant change | RT-qPCR |  |  | GaMPIX-aPDI (10 μM, 522 nm, 12.7 J/cm^2^) | < 1 log_10_ |  |
| *vicR*/ global response regulator | Two-component signal transduction system | ↑ Upregulated  (approx. 1.3-fold) | RT-qPCR | *S. mutans* | MTCC 497, Institute of Microbial Technology, Chandigarh | TBO-aPDI (20 μg ml^−1^, 630 nm, 9.1  J cm^−2^) | 1-3 log_10_ | [65] |
|  |  | ↓ Downregulated  (approx. 1.7-fold) | RT-qPCR |  |  | TBO-AgNP-aPDI (10 μg ml^−1^, 630 nm, 9.1 J cm^−2^) | > 3 log_10_ |  |

**Supplementary Table S2. Differentially expressed genes related to DNA repair upon photodynamic treatment**.

| **Gene/encoded protein** | **Function** | **Expression change** | **Method** | **Species** | **Source of bacteria** | **Photoinactivation** | **Decrease in viability** | **Refe-rence** |
| --- | --- | --- | --- | --- | --- | --- | --- | --- |
| *copA* / soluble Cu+ chaperone | Cytoplasmic copper chaperone that contributes to copper tolerance | ↑ Upregulated  (3.83-fold) | RNA microarray | *E. coli* | K12 MG1655, DSM498, DSMZ, Braunschweig | 448 nm aBL (310 J/cm^2^) | < 1 log_10_ | [95] |
| *deoB* / phosphopentomutase | Catalyzes the transfer of a phosphate group in ribose and deoxyribose | No significant change | RT-qPCR | *E. coli* | BW25113, Keio Knockout  Parent Strain | 415 nm aBL (43.2 J/cm^2^) | < 1 log_10_ | [23] |
|  |  | No significant change |  |  |  | 409 nm aBL (9.36 J/cm^2^) | < 1 log_10_ |  |
| *dinI* / DNA damage-inducible protein I | Positive modulator of RecA function,  stabilizes RecA filament | ↑ Upregulated  (2.3-fold) | RNA microarray | *E. coli* | K12 MG1655, DSM498, DSMZ, Braunschweig | 448 nm aBL (310 J/cm^2^) | < 1 log_10_ | [95] |
| *dinD* / DNA damage-inducible protein D | Disassembly of the RecA polymer after DNA repair | ↑ Upregulated  (2-fold) | RNA microarray | *E. coli* | K12 MG1655, DSM498, DSMZ, Braunschweig | 448 nm aBL (310 J/cm^2^) | < 1 log_10_ | [95] |
| *dnaE* / DNA polymerase III subunit alpha | Catalyzes the polymerase activity of the holoenzyme complex | ↑ Upregulated  T1 (1.69-fold)  T2 (1.66-fold) | RNA-seq | *C. jejuni* | NCTC11168H | 405 nm aBL (T1-15 min, 7 J/cm^2^; T2- 30 min, 14 J/cm^2^) | No statistically significant reduction (T1)  1-3 log_10_ (T2) | [22] |
| *dnaN* / β-clamp | Ensuring the high processivity of DNA polymerase III during bacterial DNA replication | ↓ Downregulated  T1 (1.38-fold)  T2 (1.93-fold) | RNA-seq | *C. jejuni* | NCTC11168H | 405 nm aBL (T1-15 min, 7 J/cm^2^; T2- 30 min, 14 J/cm^2^) | No statistically significant reduction (T1)  1-3 log_10_ (T2) | [22] |
| *dnaX* / DNA polymerase III subunit gamma/tau | DNA replication | ↓ Downregulated  T1 (2.38-fold)  T2 (2.93-fold) | RNA-seq | *C. jejuni* | NCTC11168H | 405 nm aBL (T1-15 min, 7 J/cm^2^; T2- 30 min, 14 J/cm^2^) | No statistically significant reduction (T1)  1-3 log_10_ (T2) | [22] |
| *exoA* / exodeoxyribonuclease | Putative DNA exonuclease activity | ↓ Downregulated  T1 (3.41-fold)  T2 (9.67-fold) | RNA-seq | *C. jejuni* | NCTC11168H | 405 nm aBL (T1-15 min, 7 J/cm^2^; T2- 30 min, 14 J/cm^2^) | No statistically significant reduction (T1)  1-3 log_10_ (T2) | [22] |
| *gyrA* / DNA gyrase subunit A | Type II topoisomerasea, DNA binding, cleavage | ↓ Downregulated  T1 (2.38-fold)  T2 (4.78-fold) | RNA-seq | *C. jejuni* | NCTC11168H | 405 nm aBL (T1-15 min, 7 J/cm^2^; T2- 30 min, 14 J/cm^2^) | No statistically significant reduction (T1)  1-3 log_10_ (T2) | [22] |
| *gyrB* / DNA gyrase subunit B | Type II topoisomerase, ATP hydrolysis | ↓ Downregulated  T1 (1.5-fold)  T2 (2.07-fold) | RNA-seq | *C. jejuni* | NCTC11168H | 405 nm aBL (T1-15 min, 7 J/cm^2^; T2- 30 min, 14 J/cm^2^) | No statistically significant reduction (T1)  1-3 log_10_ (T2) | [22] |
| *ligA* / DNA ligase | Catalyzes the formation of phosphodiester linkages between 5'-phosphoryl and 3'-hydroxyl groups in double-stranded DNA | ↓ Downregulated  T1 (2.62-fold)  T2 (3.55-fold) | RNA-seq | *C.jejuni* | NCTC11168H | 405 nm aBL (T1-15 min, 7 J/cm^2^; T2- 30 min, 14 J/cm^2^) | No statistically significant reduction (T1)  1-3 log_10_ (T2) | [22] |
| *pgi* / glucose-6-phosphate isomerase | Catalyzes the reversible isomerization of glucose-6-phosphate to fructose-6-phosphate | ↑ Upregulated  T2 (1.43-fold) | RNA-seq | *C. jejuni* | NCTC11168H | 405 nm aBL (T1-15 min, 7 J/cm^2^; T2- 30 min, 14 J/cm^2^) | No statistically significant reduction (T1)  1-3 log_10_ (T2) | [22] |
| *phr* / deoxyribodipyrimidine photolyase | Involved in repair of UV radiation-induced DNA damage | ↑ Upregulated (ΔExpression value: 822) | RNA-seq | *V. cholerae* | MT_VC_0001 | blue light (50 μmoles m^−2^s^−1^, fluorescent black light source, each 20 W, 45 min) | No data | [101] |
| *purA* / adenylosuccinate synthetase | Catalyzes the first committed step toward the *de novo* synthesis of AMP | ↑ Upregulated  (1.98-fold) | RT-qPCR | *E. coli* | BW25113, Keio Knockout  Parent Strain | 415 nm aBL (43.2 J/cm^2^) | < 1 log_10_ | [23] |
|  |  | ↑ Upregulated  (3.1-fold) |  |  |  | 409 nm aBL (9.36 J/cm^2^) | < 1 log_10_ |  |
| *rbfA* / 30S ribosome binding factor | Required for efficient processing of the 16S rRNA and 30S subunit assembly | ↑ Upregulated  (3.47-fold) | RT-qPCR | *E. coli* | BW25113, Keio Knockout  Parent Strain | 415 nm aBL (43.2 J/cm^2^) | < 1 log_10_ | [23] |
|  |  | ↑ Upregulated  (2.68-fold) |  |  |  | 409 nm aBL (9.36 J/cm^2^) | < 1 log_10_ |  |
| *recA* / repair protein RecA | DNA recombination and repair | ↑ Upregulated  (approx. 3-fold) | RT-qPCR | *S. agalactiae* | ATTC 27956 - isolated from infected bovine udder | RB-aPDI (0.08 μM, 515 nm, 20 J/cm^2^) | ≤ 1 log_10_ | [94] |
|  |  | No significant change | RT-qPCR | *A. baumannii* | CR-XDR-AB –  burn-wound infection  ATCC 19606 – reference strain | TBO-aPDI (0.37 mg/mL, 630nm, 180 J/cm^2^) | No reduction (growth inhibition) | [45] |
|  |  | ↑ Upregulated  (2-fold) | RT-qPCR | *S. mutans* | MTCC 497, Institute of Microbial Technology, Chandigarh | TBO-aPDI (50 μM, 630 nm, 50 J/cm^2^) | Biofilm culture,  >3 log_10_ | [97] |
|  |  | ↑ Upregulated  (3-fold) |  |  |  | NMB-aPDI (50 μM, 630 nm, 50 J/cm^2^) |  |  |
|  |  | No significant change | RT-qPCR | *E. coli* | K12, Addgene, Watertown, MA, USA | PVP curcumin-aPDI (10 and 50 µg/mL, 475 nm, 15 J/cm^2^) | 1-3 log_10_ ( 10 µg/mL)  No statistically significant reduction (50 µg/mL) | [98] |
|  |  | No significant change | RNA-seq | *E. coli* | ATCC 25922, German Collection of Microorganisms and Cell Cultures GmbH | SAPYR-aPDI (0.5 μM, 380–600 nm, 30 J/cm^2^) | < 1 log_10_ | [100] |
|  |  | ↑ Upregulated  (2.64-fold) | RNA microarray | *E. coli* | K12 MG1655, DSM498, DSMZ, Braunschweig | 448 nm aBL (310 J/cm^2^) | < 1 log_10_ | [95] |
|  |  | ↑ Upregulated  (approx. 2-fold) | RT-qPCR | *S. aureus* | USA300 JE2, NARSA | RB-aPDI (0.1 μM, 515 nm,10 J/cm^2^) | 1-3 log_10_ | [96] |
|  |  | ↑ Upregulated  (approx. 2-fold) |  |  |  | 411 nm aBL (150 J/cm^2^) | 1-3 log_10_ |  |
|  |  | ↑ Upregulated  T1 (1.51-fold)  T2 (1.57-fold) | RNA-seq | *C.jejuni* | NCTC11168H | 405 nm aBL (T1-15 min, 7 J/cm^2^; T2- 30 min, 14 J/cm^2^) | No statistically significant reduction (T1)  1-3 log_10_ (T2) | [22] |
| *recJ* / single-stranded-DNA-specific exonuclease | DNA repair, recombination, and replication | ↑ Upregulated  T1 (1.35-fold)  T2 (1.39-fold) | RNA-seq | *C. jejuni* | NCTC11168H | 405 nm aBL (T1-15 min, 7 J/cm^2^; T2- 30 min, 14 J/cm^2^) | No statistically significant reduction (T1)  1-3 log_10_ (T2) | [22] |
| *recN* / DNA repair protein | Recombinational repair of DNA double-strand breaks | ↑ Upregulated  (2.5-fold) | RNA microarray | *E. coli* | K12 MG1655, DSM498, DSMZ, Braunschweig | 448 nm aBL (310 J/cm^2^) | < 1 log_10_ | [95] |
|  |  | ↓ Downregulated  T1 (1.31-fold) | RNA-seq | *C. jejuni* | NCTC11168H | 405 nm aBL (T1-15 min, 7 J/cm^2^; T2- 30 min, 14 J/cm^2^) | No statistically significant reduction (T1)  1-3 log_10_ (T2) | [22] |
| *recX* / RecA inhibitor | Interacts directly with RecA, inhibits its ssDNA-dependent ATPase, coprotease, and DNA strand exchange activities | ↑ Upregulated  (3.03-fold) | RNA-seq | *E. coli* | ATCC 25922, German Collection of Microorganisms and Cell Cultures GmbH | SAPYR-aPDI (0.5 μM, 380–600 nm, 30 J/cm^2^) | < 1 log_10_ | [100] |
| *ruvA* / Holliday junction branch migration complex subunit RuvA | Holliday Junction recognition and binding | ↑ Upregulated  T1 (1.44-fold)  T2 (1.63- fold) | RNA-seq | *C. jejuni* | NCTC11168H | 405 nm aBL (T1-15 min, 7 J/cm^2^; T2- 30 min, 14 J/cm^2^) | No statistically significant reduction (T1)  1-3 log_10_ (T2) | [22] |
| *ruvB* / Holliday junction branch migration complex subunit RuvB | DNA helicase, branch migration | ↓ Downregulated  T1 (1.5-fold)  T2 (6-fold) | RNA-seq | *C. jejuni* | NCTC11168H | 405 nm aBL (T1-15 min, 7 J/cm^2^; T2- 30 min, 14 J/cm^2^) | No statistically significant reduction (T1)  1-3 log_10_ (T2) | [22] |
| *ruvC* / crossover junction endodeoxyribonuclease | Holliday Junction resolution | ↓ Downregulated  T1 (1.97-fold)  T2 (4.26-fold) | RNA-seq | *C. jejuni* | NCTC11168H | 405 nm aBL (T1-15 min, 7 J/cm^2^; T2- 30 min, 14 J/cm^2^) | No statistically significant reduction (T1)  1-3 log_10_ (T2) | [22] |
| *ssb* / single-stranded DNA-binding protein | DNA replication, repair, and recombination | ↓ Downregulated  T1 (4.71-fold)  T2 (4.27-fold) | RNA-seq | *C. jejuni* | NCTC11168H | 405 nm aBL (T1-15 min, 7 J/cm^2^; T2- 30 min, 14 J/cm^2^) | No statistically significant reduction (T1)  1-3 log_10_ (T2) | [22] |
| *umuC* / DNA polymerase V catalytic protein | DNA polymerase involved in translesion replication and mutagenesis | No significant change | RT-qPCR | *E. coli* | K12, Addgene, Watertown, MA, USA | PVP curcumin-aPDI (10 and 50 µg/mL, 475 nm, 15 J/cm^2^) | 1-3 log_10_ ( 10 µg/mL)  No statistically significant reduction (50 µg/mL) | [98] |
|  |  | ↑ Upregulated  (approx. 2-fold) | RT-qPCR | *S. aureus* | USA300 JE2, NARSA | RB-aPDI (0.1 μM, 515 nm, 10 J/cm^2^) | 1-3 log_10_ | [96] |
|  |  | ↑ Upregulated  (approx. 2-fold) |  |  |  | 411 nm aBL (150 J/cm^2^) | 1-3 log_10_ |  |
| *umuD* / DNA polymerase V protein | Regulates DNA damage and mutagenesis | ↑ Upregulated  (4-fold) | RT-qPCR | *E. coli* | BW25113, Keio Knockout  Parent Strain | 415 nm aBL (43.2 J/cm^2^) | < 1 log_10_ | [23] |
|  |  | ↑ Upregulated  (3.36-fold) |  |  |  | 409 nm aBL (9.36 J/cm^2^) | < 1 log_10_ |  |
| *yebG* / DNA damage-inducible protein | DNA damage-inducible gene of the SOS regulon | ↑ Upregulated  (2.1-fold) | RNA microarray | *E. coli* | K12 MG1655, DSM498, DSMZ, Braunschweig | 448 nm aBL (310 J/cm^2^) | < 1 log_10_ | [95] |
| *zraP* / signaling pathway modulator | Accessory protein and modulator of the Zra signaling pathway | ↑ Upregulated  (2.93-fold) | RNA microarray | *E. coli* | K12 MG1655, DSM498, DSMZ, Braunschweig | 448 nm aBL (310 J/cm^2^) | < 1 log_10_ | [95] |

**Supplementary Table S3. Differentially expressed genes related to detoxification and stress response upon photodynamic treatment.**

| **Gene/encoded protein** | **Function** | **Expression change** | **Method** | **Species** | **Source of**  **bacteria** | **Photoinactivation** | **Decrease in viability** | **Refe-rence** |
| --- | --- | --- | --- | --- | --- | --- | --- | --- |
| *σV* / SigV | Extracytoplasmic sigma factor | ↑ Upregulated  (to 38.7-fold) | RT-qPCR | *E. faecalis* | ATTC 29212  A1, A2 – clinical isolates, Karolinska Hospital culture collections  UmID1, UmID2, UmID3 - infected dental root-canals, Umeå bacterial collection | TMPyP-aPDI (0.015 µM, 445 nm, 210 J/cm^2^) | 1-3 log_10_ | [77] |
| *ahpC* / alkyl hydroperoxide reductase C) | Organic peroxides reduction | Expression not aligned with bacterial growth | RT-qPCR | *C. acnes* | ATCC 6919 | ALA-aPDI (0.05, 0.1, 0.5, 1.0, 2.5 mmol/L, 633 nm, 3 J/cm^2^) | No decrease (0.05-0.5 mmol/L)  < 1 log_10_ (1 and 2.5 mmol/L) | [111] |
|  |  | ↑ Upregulated  (approx. 2.75-fold) | RT-qPCR | *S. enterica* serovar Typhimurium | DS88, University of Helsinki | Chl-aPDI (1.5×10^-5^ M, 405 nm, 38 J/cm^2^) | 1-3 log_10_ | [108] |
|  |  | ↑ Upregulated at T2  (5.22-fold) | RNA-seq | *C. jejuni* | NCTC11168H | 405 nm aBL (T1-15 min, 7 J/cm^2^; T2- 30 min, 14 J/cm^2^) | No statistically significant reduction (T1)  1-3 log_10_ (T2) | [22] |
| *atpC* / ATP synthase epsilon chain | Inhibition of oxidative respiration | ↑ Upregulated  (approx. 2.75-fold) | RT-qPCR | *S. enterica* serovar Typhimurium | DS88, University of Helsinki | Chl-aPDI (1.5×10^-5^ M, 405 nm, 38 J/cm^2^) | 1-3 log_10_ | [108] |
| *ccoN* / cbb₃-type cytochrome c oxidase | Bacterial electron transport chain | ↓ Downregulated at T2  (2.4-fold) | RNA-seq | *C. jejuni* | NCTC11168H | 405 nm aBL (T1-15 min, 7 J/cm^2^; T2- 30 min, 14 J/cm^2^) | No statistically significant reduction (T1)  1-3 log_10_ (T2) | [22] |
| *cj0379c (msrP)* / methionine sulfoxide reductase | Protection of proteins from oxidative damage | ↑ Upregulated at T2  (6.6-fold) | RNA-seq | *C. jejuni* | NCTC11168H | 405 nm aBL (T1-15 min, 7 J/cm^2^; T2- 30 min, 14 J/cm^2^) | No statistically significant reduction (T1)  1-3 log_10_ (T2) | [22] |
| *cj0358* / cytochrome c peroxidase | Peroxidase activity | ↑ Upregulated  T1 (2.86-fold)  T2 (1.85-fold) | RNA-seq | *C. jejuni* | NCTC11168H | 405 nm aBL (T1-15 min, 7 J/cm^2^; T2- 30 min, 14 J/cm^2^) | No statistically significant reduction (T1)  1-3 log_10_ (T2) | [22] |
| *cj0264c* / TMAO reductase | Alternative electron transport | ↑ Upregulated  T1 (4.38-fold)  T2 (4.07-fold) | RNA-seq | *C. jejuni* | NCTC11168H | 405 nm aBL (T1-15 min, 7 J/cm^2^; T2- 30 min, 14 J/cm^2^) | No statistically significant reduction (T1)  1-3 log_10_ (T2) | [22] |
| *cj0265c* / TMAO reductase | Alternative electron transport | ↑ Upregulated  T1 (6.90-fold)  T2 (8.40-fold) | RNA-seq | *C. jejuni* | NCTC11168H | 405 nm aBL (T1-15 min, 7 J/cm^2^; T2- 30 min, 14 J/cm^2^) | No statistically significant reduction (T1)  1-3 log_10_ (T2) | [22] |
| *csrA* / translational regulator CsrA | Translation control of stress-related proteins | ↑ Upregulated at T2  (6.84-fold) | RNA-seq | *C. jejuni* | NCTC11168H | 405 nm aBL (T1-15 min, 7 J/cm^2^; T2- 30 min, 14 J/cm^2^) | No statistically significant reduction (T1)  1-3 log_10_ (T2) | [22] |
| *cydA* / terminal oxidase subunits | Bacterial electron transport chain | ↓ Downregulated at T2  (4.11-fold) | RNA-seq | *C. jejuni* | NCTC11168H | 405 nm aBL (T1-15 min, 7 J/cm^2^; T2- 30 min, 14 J/cm^2^) | No statistically significant reduction (T1)  1-3 log_10_ (T2) | [22] |
| *cydB* / terminal oxidase subunits | Bacterial electron transport chain | ↓ Downregulated at T2  (3.20-fold) | RNA-seq | *C. jejuni* | NCTC11168H | 405 nm aBL (T1-15 min, 7 J/cm^2^; T2- 30 min, 14 J/cm^2^) | No statistically significant reduction (T1)  1-3 log_10_ (T2) | [22] |
| *dnaK* / chaperone | Heat shock response | ↑ Upregulated  (to 30.1-fold) | RT-qPCR | *E. faecalis* | ATTC 29212  A1, A2 – clinical isolates, Karolinska Hospital culture collections  UmID1, UmID2, UmID3 - infected dental root-canals, Umeå bacterial collection | TMPyP-aPDI (0.015 µM, 445 nm, 210 J/cm^2^) | 1-3 log_10_ | [77] |
| *dps* / DNA-binding protein | Oxidative stress protection | ↑ Upregulated  (> 2-fold) | RT-qPCR | *E. faecalis* | ATTC 29212  A1, A2 – clinical isolates, Karolinska Hospital culture collections  UmID1, UmID2, UmID3 - infected dental root-canals, Umeå bacterial collection | TMPyP-aPDI (0.015 µM, 445 nm, 210 J/cm^2^) | 1-3 log_10_ | [77] |
| *dsbA2* / disulfide bond system | Protein folding, oxidative stress resistance | ↑ Upregulated at T2  (1.79-fold) | RNA-seq | *C. jejuni* | NCTC11168H | 405 nm aBL (T1-15 min, 7 J/cm^2^; T2- 30 min, 14 J/cm^2^) | No statistically significant reduction (T1)  1-3 log_10_ (T2) | [22] |
| *dsbB* / disulfide bond system |  | ↑ Upregulated  T1 (2.36-fold)  T2 (3.45-fold) | RNA-seq | *C. jejuni* | NCTC11168H | 405 nm aBL (T1-15 min, 7 J/cm^2^; T2- 30 min, 14 J/cm^2^) | No statistically significant reduction (T1)  1-3 log_10_ (T2) | [22] |
| *dsbD* / disulfide bond system |  | ↑ Upregulated  T1 (1.57-fold)  T2 (4.88-fold) | RNA-seq | *C. jejuni* | NCTC11168H | 405 nm aBL (T1-15 min, 7 J/cm^2^; T2- 30 min, 14 J/cm^2^) | No statistically significant reduction (T1)  1-3 log_10_ (T2) | [22] |
| *grxA* / glutaredoxin | Protection against oxidative stress | ↑ Upregulated  (approx. 1.8-fold) | RT-qPCR | *S. enterica* serovar Typhimurium | DS88, University of Helsinki | Chl-aPDI (1.5×10^-5^ M, 405 nm, 38 J/cm^2^) | 1-3 log_10_ | [108] |
| *hfq* / RNA-binding protein | Post-transcriptional regulation | ↑ Upregulated  (> 6-fold) | RT-qPCR | *K. pneumoniae* | KPPR1 (ATCC 43816) – reference strain  ST258 – clinical strain | PSIR-3-aPDI (4 µg/mL, white LED, 17 µW/cm^2^) | > 3 log_10_ | [110] |
| *hspR* / heat shock transcriptional regulator HspR | Heat shock and oxidative stress genes regulation | ↑ Upregulated  T1 (3.09-fold)  T2 (7.32-fold) | RNA-seq | *C. jejuni* | NCTC11168H | 405 nm aBL (T1-15 min, 7 J/cm^2^; T2- 30 min, 14 J/cm^2^) | No statistically significant reduction (T1)  1-3 log_10_ (T2) | [22] |
| *hypR* / oxidative stress regulator | Transcriptional activator | ↑ Upregulated  (approx. 4-fold for ATCC 29212) | RT-qPCR | *E. faecalis* | ATTC 29212  A1, A2 – clinical isolates, Karolinska Hospital culture collections  UmID1, UmID2, UmID3 - infected dental root-canals, Umeå bacterial collection | TMPyP-aPDI (0.015 µM, 445 nm, 210 J/cm^2^) | 1-3 log_10_ | [77] |
| *katA* / catalase | Hydrogen peroxide decomposition | ↑ Upregulated  T1 (8.03-fold)  T2 (111.82-fold) | RNA-seq | *C. jejuni* | NCTC11168H | 405 nm aBL (T1-15 min, 7 J/cm^2^; T2- 30 min, 14 J/cm^2^) | No statistically significant reduction (T1)  1-3 log_10_ (T2) | [22] |
| *katE* / catalase | Hydrogen peroxide decomposition | ↑ Upregulated  (7.1-fold) | RT-qPCR | *A. baumannii* | ATCC 19606 | 462 nm blue light (6-10 µmol photons · m^-2^ ·s^-1^) | No data | [49] |
| *mfrA* / periplasmic fumarate reductase | Alternative electron acceptor | ↑ Upregulated  T1 (2.82-fold)  T2 (3.33-fold) | RNA-seq | *C. jejuni* | NCTC11168H | 405 nm aBL (T1-15 min, 7 J/cm^2^; T2- 30 min, 14 J/cm^2^) | No statistically significant reduction (T1)  1-3 log_10_ (T2) | [22] |
| *mfrB* / periplasmic fumarate reductase |  | ↑ Upregulated  T1 (4.85-fold)  T2 (4.30-fold) | RNA-seq | *C. jejuni* | NCTC11168H | 405 nm aBL (T1-15 min, 7 J/cm^2^; T2- 30 min, 14 J/cm^2^) | No statistically significant reduction (T1)  1-3 log_10_ (T2) | [22] |
| *mfrE* / periplasmic fumarate reductase |  | ↑ Upregulated  T1 (2.23-fold)  T2 (2.22-fold) | RNA-seq | *C. jejuni* | NCTC11168H | 405 nm aBL (T1-15 min, 7 J/cm^2^; T2- 30 min, 14 J/cm^2^) | No statistically significant reduction (T1)  1-3 log_10_ (T2) | [22] |
| *napG* / putative ferrodoxin | Electron transfer components of periplasmic nitrate reductase | ↑ Upregulated  T1 (2.08-fold)  ↓ Downregulated  T2 (1.07-fold) | RNA-Seq | *C. jejuni* | NCTC11168H | 405 nm aBL (T1-15 min, 7 J/cm^2^; T2- 30 min, 14 J/cm^2^) | No statistically significant reduction (T1)  1-3 log_10_ (T2) | [22] |
| *napH* / putative ferrodoxin |  | ↑ Upregulated  T1 (2.87-fold)  T2 (1.65-fold) | RNA-Seq | *C. jejuni* | NCTC11168H | 405 nm aBL (T1-15 min, 7 J/cm^2^; T2- 30 min, 14 J/cm^2^) | No statistically significant reduction (T1)  1-3 log_10_ (T2) | [22] |
| *oxyR* / oxidative stress response regulator | Antioxidant defense genes regulation | ↑ Upregulated  (approx. 2-fold at 0.5  mmol/L)  (approx. 3.5-fold at  2.5 mmol/L) | RT-qPCR | *C. acnes* | ATCC 6919 | ALA-aPDI (0.05, 0.1, 0.5, 1.0, 2.5 mmol/L, 633 nm, 3 J/cm^2^) | No decrease (0.05-0.5 mmol/L)  < 1 log_10_ (1 and 2.5 mmol/L) | [111] |
|  |  | ↑ Upregulated  (2.0-fold) | RT-qPCR | *K. pneumoniae* | KPPR1 (ATCC 43816) – reference strain  ST258 – clinical strain | PSIR-3-aPDI (4 µg/mL, white LED, 17 µW/cm^2^) | > 3 log_10_ | [110] |
|  |  | ↑ Upregulated  (8.5-fold) | RT-qPCR | *P. gingivalis* | Clinical isolates from root canal from patients treated with PAD | TBO-aPDI (6.25 μg/ml, 171.87 J/cm^2^, 635 nm, 220 mW) | No statistically significant reduction | [109] |
|  |  | ↑ Upregulated  (12.3-fold) |  |  |  | ICG-aPDI (15.6 μg/mL, 15.6 J/cm^2^, 810 nm, 250 mW) | No statistically significant reduction |  |
|  |  | ↑ Upregulated  ( 5.6-fold) |  |  |  | MB-aPDI (25 μg/mL, 93.75 J/cm^2^, 660 nm, 150 mW) | No statistically significant reduction |  |
|  |  | ↑ Upregulated  (approx. 1.6-fold) | RT-qPCR | *S. enterica* serovar Typhimurium | DS88, University of Helsinki | Chl-aPDI (1.5×10^-5^ M, 405 nm, 38 J/cm^2^) | 1-3 log_10_ | [108] |
| *PPA0097* / catalase | Hydrogen peroxide decomposition | ↑ Upregulated  (approx. 1.5-fold at 0.1 mmol/L)  (approx. 2.5-fold at 0.5 mmol/L)  (approx. 3.75-fold at 1.0 mmol/L)  (approx. 7.0-fold at 2.5 mmol/L) | RT-qPCR | *C. acnes* | ATCC 6919 | ALA-aPDI (0.05, 0.1, 0.5, 1.0, 2.5 mmol/L, 633 nm, 3 J/cm^2^) | No decrease (0.05-0.5 mmol/L)  < 1 log_10_ (1 and 2.5 mmol/L) | [111] |
| *recA* / recombinase A | DNA repair, SOS response | No significant change | RT-qPCR | *S. Enteritidis* | ATCC 13076 (CDC) | 405 nm aBL (72 J/cm^2^, 4°C) | No statistically significant reduction | [18] |
|  |  | ↑ Upregulated in illuminated & nonilluminated |  | *S. Saintpaul* | ATCC 9712 (cystitis, Panama) |  | No statistically significant reduction |  |
| *relA* / RelA | Stringent response regulator | Variable (↑/↓) | RT-qPCR | *E. faecalis* | ATTC 29212  A1, A2 – clinical isolates, Karolinska Hospital culture collections  UmID1, UmID2, UmID3 - infected dental root-canals, Umeå bacterial collection | TMPyP-aPDI (0.015 µM, 445 nm, 210 J/cm^2^) | 1-3 log_10_ | [77] |
| *rpoE* / extracytoplasmic sigma factor | Extracytoplasmic stress-response regulation | ↑ Upregulated  (>6-fold) | RT-qPCR | *K. pneumoniae* | KPPR1 (ATCC 43816) – reference strain  ST258 – clinical strain | PSIR-3-aPDI (4 µg/mL, white LED, 17 µW/cm^2^) | > 3 log_10_ | [110] |
| *rpoS* / sigma factor RpoS | General stress response | No significant change | RT-qPCR | *S. Enteritidis* | ATCC 13076 (CDC) | 405 nm aBL (72 J/cm^2^, 4°C) | No statistically significant reduction | [18] |
|  |  | ↑ Upregulated  in illuminated & nonilluminated |  | *S. Saintpaul* | ATCC 9712 (cystitis, Panama) |  | No statistically significant reduction |  |
| *rrc* / desulforuberythrin-like protein | Alternative electron transport chain | ↑ Upregulated  T1 (2.48-fold)  T2 (3.34-fold) | RNA-seq | *C. jejuni* | NCTC11168H | 405 nm aBL (T1-15 min, 7 J/cm^2^; T2- 30 min, 14 J/cm^2^) | No statistically significant reduction (T1)  1-3 log_10_ (T2) | [22] |
| *sodA* /superoxide dismutase A | Superoxide radicals neutralization | ↑ Upregulated  (approx. 2.0-fold at 0.5 mmol/L)  (approx. 5.0-fold at 1.0 mmol/L)  (approx. 6.0-fold at 2.5 mmol/L) | RT-qPCR | *C. acnes* | ATCC 6919 | ALA-aPDI (0.05, 0.1, 0.5, 1.0, 2.5 mmol/L, 633 nm, 3 J/cm^2^) | No decrease (0.05-0.5 mmol/L)  < 1 log_10_ (1 and 2.5 mmol/L) | [111] |
|  |  | No significant change | RT-qPCR | *K. pneumoniae* | KPPR1 (ATCC 43816) – reference strain  ST258 – clinical strain | PSIR-3-aPDI (4 µg/mL, white LED, 17 µW/cm^2^) | > 3 log_10_ | [110] |
|  |  | No significant change | RT-qPCR | *S. Enteritidis* | ATCC 13076 (CDC) | 405 nm aBL (72 J/cm^2^, 4°C) | No statistically significant reduction | [18] |
|  |  | ↑ Upregulated  in illuminated & nonilluminated |  | *S. Saintpaul* | ATCC 9712 (cystitis, Panama) |  | No statistically significant reduction |  |
| *soxR* / redox-sensitive transcriptional activator | Response to superoxide stress | No significant change | RT-qPCR | *S. Enteritidis* | ATCC 13076 (CDC) | 405 nm aBL (72 J/cm^2^, 4°C) | No statistically significant reduction | [18] |
|  |  | ↑ Upregulated in illuminated & nonilluminated |  | *S. Saintpaul* | ATCC 9712 (cystitis, Panama) |  | No statistically significant reduction |  |
| STM0225 (*skp*) / chaperone | Stress adaptation function | ↑ Upregulated  (approx. 1.75-fold) | RT-qPCR | *S. enterica* serovar Typhimurium | DS88, University of Helsinki | Chl-aPDI (1.5×10^-5^ M, 405 nm, 38 J/cm^2^) | 1-3 log_10_ | [108] |
| *sulA* / SulA | Inhibitor of cell division | ↑ Upregulated  (approx. 1.8-fold) | RT-qPCR | *S. enterica* serovar Typhimurium | DS88, University of Helsinki | Chl-aPDI (1.5×10^-5^ M, 405 nm, 38 J/cm^2^) | 1-3 log_10_ | [108] |
| *trxB* / thioredoxin reductase | Maintains redox homeostasis | ↑ Upregulated  T1 (2.57-fold)  T2 (3.96-fold) | RNA-seq | *C. jejuni* | NCTC11168H | 405 nm aBL (T1-15 min, 7 J/cm^2^; T2- 30 min, 14 J/cm^2^) | No statistically significant reduction (T1)  1-3 log_10_ (T2) | [22] |

**Supplementary Table S4. Differentially expressed genes related to heat shock proteins upon photodynamic treatment**.

| **Gene/encoded protein** | **Function** | **Expression change** | **Method** | **Species** | **Source of**  **bacteria** | **Photoinactivation** | **Decrease in viability** | **Refe-rence** |
| --- | --- | --- | --- | --- | --- | --- | --- | --- |
| *clpB* / chaperone | Protein folding and stress response | ↑ Upregulated  T1 (9.31-fold)  T2 (20.04-fold) | RNA-seq | *C. jejuni* | NCTC11168H | 405 nm aBL (T1-15 min, 7 J/cm^2^; T2- 30 min, 14 J/cm^2^) | No statistically significant reduction (T1)  1-3 log_10_ (T2) | [22] |
|  |  | ↑ Upregulated  (13.3-fold) | RNA-seq | *E. coli* | ATCC 25922, German Collection of Microorganisms and Cell Cultures GmbH | SAPYR-aPDI (0.5 μM, 380–600 nm, 30 J/cm^2^) | < 1 log_10_ | [100] |
| *cpxP* / Cpx two-component envelope stress response system | Periplasmic chaperonin | ↑ Upregulated  (97-fold) | RNA-seq | *E. coli* | ATCC 25922, German Collection of Microorganisms and Cell Cultures GmbH | SAPYR-aPDI (0.5 μM, 380–600 nm, 30 J/cm^2^) | < 1 log_10_ | [100] |
| *cspI* / cold shock protein | Chaperone | ↑ Upregulated  (51.7-fold) | RNA-seq | *E. coli* | ATCC 25922, German Collection of Microorganisms and Cell Cultures GmbH | SAPYR-aPDI (0.5 μM, 380–600 nm, 30 J/cm^2^) | < 1 log_10_ | [100] |
| *dnaK* / chaperone | Heat shock Response | ↑ Upregulated  (5.6-fold for CR-XDR-AB)  ↑ Upregulated  (4.4-fold ATCC 19606) | RNA-seq | *A. baumannii* | CR-XDR-AB –  burn-wound infection  ATCC 19606 – reference strain | TBO-aPDI (0.37 mg/mL, 630nm, 180 J/cm^2^) | No reduction (growth inhibition) | [45] |
|  |  | ↑ Upregulated  T1 (8.44-fold)  T2 (8.49-fold) | RNA-seq | *C. jejuni* | NCTC11168H | 405 nm aBL (T1-15 min, 7 J/cm^2^; T2- 30 min, 14 J/cm^2^) | No statistically significant reduction (T1)  1-3 log_10_ (T2) | [22] |
| *gadA* / glutamate decarboxylase alpha | Maintaining a near-neutral intracellular pH when cells are exposed to extremely acidic conditions | ↓ Downregulated  (27.9-fold) | RNA-seq | *E. coli* | ATCC 25922, German Collection of Microorganisms and Cell Cultures GmbH | SAPYR-aPDI (0.5 μM, 380–600 nm, 30 J/cm^2^) | < 1 log_10_ | [100] |
| *gadB* / glutamate decarboxylase beta | Maintaining a near-neutral intracellular pH when cells are exposed to extremely acidic conditions | ↓ Downregulated  (36.8-fold) | RNA-seq | *E. coli* | ATCC 25922, German Collection of Microorganisms and Cell Cultures GmbH | SAPYR-aPDI (0.5 μM, 380–600 nm, 30 J/cm^2^) | < 1 log_10_ | [100] |
| *gadC* / glutamic acid:γ-aminobutyrate antiporter | Part of the glutamate-dependent acid resistance system 2 (AR2) which confers resistance to extreme acid conditions | ↓ Downregulated  (27.9-fold) | RNA-seq | *E. coli* | ATCC 25922, German Collection of Microorganisms and Cell Cultures GmbH | SAPYR-aPDI (0.5 μM, 380–600 nm, 30 J/cm^2^) | < 1 log_10_ | [100] |
| *groEL* / chaperone | Heat shock Response | ↑ Upregulated  T1 (3.60-fold)  T2 (4.68-fold) | RNA-seq | *C. jejuni* | NCTC11168H | 405 nm aBL (T1-15 min, 7 J/cm^2^; T2- 30 min, 14 J/cm^2^) | No statistically significant reduction (T1)  1-3 log_10_ (T2) | [22] |
| *grpE* / HSP-70 cofactor | Response to hyperosmotic and heat shock by preventing the aggregation of stress-denatured proteins | ↑ Upregulated  T1 (34.2-fold)  T2 (47.56-fold) | RNA-seq | *C. jejuni* | NCTC11168H | 405 nm aBL (T1-15 min, 7 J/cm^2^; T2- 30 min, 14 J/cm^2^) | No statistically significant reduction (T1)  1-3 log_10_ (T2) | [22] |
| *groES* / co-chaperonin GroES | Protein folding assisting | ↑ Upregulated  T1 (9.65-fold)  T2 (67.68-fold) | RNA-seq | *C. jejuni* | NCTC11168H | 405 nm aBL (T1-15 min, 7 J/cm^2^; T2- 30 min, 14 J/cm^2^) | No statistically significant reduction (T1)  1-3 log_10_ (T2) | [22] |
| *hdeA, hdeB, hdeD* / periplasmic acid stress chaperone | Prevent the aggregation of periplasmic proteins at acidic pH | ↓ Downregulated  (13.9-fold, 16-fold, 21.1-fold) | RNA-seq | *E. coli* | ATCC 25922, German Collection of Microorganisms and Cell Cultures GmbH | SAPYR-aPDI (0.5 μM, 380–600 nm, 30 J/cm^2^) | < 1 log_10_ | [100] |
| *hslO* / molecular chaperone Hsp33 | Protects both thermally unfolding and oxidatively damaged proteins from irreversible aggregation | ↑ Upregulated  (15.4-fold) | RNA-seq | *E. coli* | ATCC 25922, German Collection of Microorganisms and Cell Cultures GmbH | SAPYR-aPDI (0.5 μM, 380–600 nm, 30 J/cm^2^) | < 1 log_10_ | [100] |
| *hrcA* / heat-inducible transcription repressor | Prevents heat-shock induction of operons *grpE*, *dnaK*, etc. | ↑ Upregulated  T1 (38.68-fold)  T2 (159.63-fold) | RNA-seq | *C. jejuni* | NCTC11168H | 405 nm aBL (T1-15 min, 7 J/cm^2^; T2- 30 min, 14 J/cm^2^) | No statistically significant reduction (T1)  1-3 log_10_ (T2) | [22] |
| *hspR* / heat shock transcriptional regulator HspR | Regulation of expression of heat-shock genes | ↑ Upregulated  T1 (3.1-fold )  T2 (7.32-fold) | RNA-seq | *C. jejuni* | NCTC11168H | 405 nm aBL (T1-15 min, 7 J/cm^2^; T2- 30 min, 14 J/cm^2^) | No statistically significant reduction (T1)  1-3 log_10_ (T2) | [22] |
| *ibpA* / small heat shock protein | Heat shock Response | ↑ Upregulated  (38.6-fold) | RNA-seq | *E. coli* | ATCC 25922, German Collection of Microorganisms and Cell Cultures GmbH | SAPYR-aPDI (0.5 μM, 380–600 nm, 30 J/cm^2^) | < 1 log_10_ | [100] |
| *ibpB* / small heat shock protein |  | ↑ Upregulated  (98-fold) | RNA-seq | *E. coli* | ATCC 25922, German Collection of Microorganisms and Cell Cultures GmbH | SAPYR-aPDI (0.5 μM, 380–600 nm, 30 J/cm^2^) | < 1 log_10_ | [100] |
| *nark* / small heat shock protein | Probably: Nitrate transporter | ↓ Downregulated  (9.24 fold) | RNA-seq | *S. aureus* | BUSA2288 – MRSA clinical isolate from nasal passage | 465 nm blue light (250 J/cm²) | No data | [117] |
| SAR 2639 / copper chaperone | Not characterised | ↓ Downregulated  (*) | RNA-seq | *S. aureus* | MRSA 8325-4, Wellman Center for Photomedicine, Boston  MRSA 252, ATCC | 460 nm aBL (120 J/cm^2^) | < 1 log_10_ | [116] |
| *spy* / ATP-independent periplasmic chaperone | Decreases protein aggregation and helps protein refolding | ↑ Upregulated  (119-fold) | RT-qPCR | *E. coli* | ATCC 25922, German Collection of Microorganisms and Cell Cultures GmbH | SAPYR-aPDI (0.5 μM, 380–600 nm, 30 J/cm^2^) | < 1 log_10_ | [100] |

*Authors did not provide fold change values

**Supplementary Table S5. Differentially expressed genes related to antimicrobial resistance and efflux pumps upon photodynamic treatment**.

| **Gene/encoded protein** | **Function** | **Expression change** | **Method** | **Species** | **Source of**  **bacteria** | **Photoinactivation** | **Decrease in viability** | **Refe-rence** |
| --- | --- | --- | --- | --- | --- | --- | --- | --- |
| *blaZ* / beta-lactamase | Resistance to β-lactamase antibiotics | ↓ Downregulated  (*) | RNA-seq | *S. aureus* | MRSA 8325-4, Wellman Center for Photomedicine, Boston  MRSA 252, ATCC | 460 nm aBL (120 J/cm^2^) | < 1 log_10_ | [116] |
| *bla* / beta-lactamase | Resistance to β-lactamase antibiotics | ↓ Downregulated  (approx. 2-7-fold) | RT-qPCR | *E. coli* | DH5α – reference strain | 450 nm aBL (﻿10 W/m^2^) | No influence on growth | [126] |
| *bla_OXA-23_* / beta-lactamase OXA 23 | Resistance to oxacillin and other β-lactam antibiotics | ↓ Downregulated  (4.1-fold) | RT-qPCR | *A. baumannii* | ATCC 19606 | Rutin-Gal(III)-aPDI (32 µg/mL, 405 nm±10 nm, 60 s) | > 3 log_10_ | [127] |
|  |  | ↓ Downregulated  (7.8-fold) |  |  |  | Quercetin-aPDI (128 µg/mL, 405 nm±10 nm, 60 s) | 1-3 log_10_ |  |
| *cj1180c* / multidrug resistance ABC transporter ATP-binding | Probable involvement in the removal of toxic compounds from the cell | ↓ Downregulated  T1 (3.23-fold)  T2 (9.73-fold) | RNA-Seq | *C. jejuni* | NCTC11168H | 405 nm aBL (T1-15 min, 7 J/cm^2^; T2- 30 min, 14 J/cm^2^) | No statistically significant reduction (T1)  1-3 log_10_ (T2) | [22] |
| DJ41_1581.t01 / unknown | Carbapenem-associated resistance protein | ↑ Upregulated  (2.67-fold) | RNA-seq | *A. baumannii* | ATCC 19606 | 462 nm blue light (6-10 µmol photons · m^-2^ ·s^-1^) | No data | [49] |
| *erm*(41) /﻿ methyl- transferase | Modifies A2058 or A2058 in the peptidyltransferase region of the 23S rRNA, preventing the binding of macrolides | ↓ Downregulated for  both concentrations  (approx. 2-fold) | RT-qPCR | *M. abscessus* | ATCC 19977 | ALA-aPDI (40, 100 mg/mL, 80 J/cm^2^, 635 nm) | No statistically significant reduction (40 mg/mL )  < 1 log_10_ (100 mg/mL) | [130] |
| *emrA, emrB* / components of efflux pump resistance-nodulation-division (RND) | Hypothetic function: resistance to colistin and adaptation to osmotic stress | ↓ Downregulated  (4.76-fold, 2.48-fold) | RNA-seq | *A. baumannii* | ATCC 19606 | 462 nm blue light (6-10 µmol photons · m^-2^ ·s^-1^) | No data | [49] |
| *fmtA* / teichoic acid D-alanine hydrolase | Catalyzes the liberation of D-alanyl moieties present on wall teichoic acid (WTA) and lipoteichoic acid (LTA). Modulate resistance to methicillin | ↑ Upregulated  (1.12-fold) | RNA-seq | *S. aureus* | HG003 - laboratory strain | MB-aPDI (33 µM, 2.4 J/cm^2^) | > 3 log_10_ | [20] |
| *fosB* / FosB | Resistance to fosfomycin | ↑ Upregulated  (10.2-fold) | RNA-seq | *S. aureus* | HG003 - laboratory strain | MB-aPDI (33 µM, 2.4 J/cm^2^) | > 3 log_10_ | [20] |
| MAB_1409c / membrane transporter proteins | Efflux pump | ↓ Downregulated for  both concentrations  (approx. 4-fold) | RT-qPCR | *M. abscessus* | ATCC 19977 | ALA-aPDI (40, 100 mg/mL, 80 J/cm^2^, 635 nm) | No statistically significant reduction (40 mg/mL )  < 1 log_10_ (100 mg/mL) | [130] |
| MAB_3142c / membrane transporter proteins |  | ↓ Downregulated for both concentrations  (> 10-fold) | RT-qPCR | *M. abscessus* | ATCC 19977 | ALA-aPDI (40, 100 mg/mL, 80 J/cm^2^, 635 nm) | No statistically significant reduction (40 mg/mL )  < 1 log_10_ (100 mg/mL) | [130] |
| *mdeA* / multidrug efflux MFS transporter MdeA | Efflux pump | ↑ Upregulated  (1.77-fold for MSSA  strain)  (1.59-fold for MRSA  strain) | RT-qPCR | *S. aureus* | Newman (MSSA)  USA300 (MRSA) | TBO-aPDI (0.5 mM, 635 nm, 40 J/cm^2^) | 1-3 log_10_ | [134] |
| *mecA* / penicillin-binding protein (PBP) | Resistance to methicillin | ↓ Downregulated  (2-fold for MRSA/BAA-  1556 strain) | RT-qPCR | *S. aureus* | JD004, JD39452 - MRSA strains from patients’ wounds  BAA-1556 - American Type Culture Collection | ICG-aPDI (﻿25 µg/mL, ﻿100 J/cm^2^, 65.5 mW/cm^2^) | 1-3 log_10_  (JD004)  < 1 log_10_ (JD39452)  ≤ 1 log_10_ (BAA-1556) | [125] |
| *mepA* / multidrug export protein MepA | Multidrug resistance efflux of norfloxacin, ciprofloxacin and involved in the production of protein A (spa) and toxins alpha, beta and delta | ↑ Upregulated  (1.57-fold for MSSA)  (6.29-fold for MRSA) | RT-qPCR | *S. aureus* | Newman (MSSA)  USA300 (MRSA) | TBO-aPDI (0.5 mM, 635 nm, 40 J/cm^2^) | 1-3 log_10_ | [134] |
| *mgrA* / multiple-gene regulator | Regulatory protein involved in autolytic activity, multidrug resistance and virulence | ↑ Upregulated  (3.3-fold) | RNA-seq | *S. aureus* | HG003 - laboratory strain | MB-aPDI (33 µM, 2.4 J/cm^2^) | > 3 log_10_ | [20] |
| *norA* / quinolone resistance protein NorA | Involved in quinolone resistance. May constitute a membrane-associated active efflux pump of hydrophilic quinolones | ↓ Downregulated  (5.44-fold for MSSA)  ↑ Upregulated  (2.90-fold for MRSA) | RT-qPCR | *S. aureus* | Newman (MSSA)  USA300 (MRSA) | TBO-aPDI (0.5 mM, 635 nm, 40 J/cm^2^) | 1-3 log_10_ | [134] |
| *norB* / quinolone resistance protein NorB | Multidrug efflux pump involved in quinolone resistance | ↓ Downregulated  (3.77-fold for MSSA) (3.02-fold for MRSA) | RT-qPCR | *S. aureus* | Newman (MSSA)  USA300 (MRSA) | TBO-aPDI (0.5 mM, 635 nm, 40 J/cm^2^) | 1-3 log_10_ | [134] |
| *saeS* / histidine protein kinase | Member of the two-component regulatory system SaeR/SaeS involved in the regulation of staphylococcal virulence factors | ↑ Upregulated  4.0-fold) | RNA-seq | *S. aureus* | HG003 - laboratory strain | MB-aPDI (33 µM, 2.4 J/cm^2^) | > 3 log_10_ | [20] |
| SAOUHSC_00647 / multidrug resistance ABC transporter ATP-binding | Probable involvement in the removal of toxic compounds from the cell | ↓ Downregulated  (4.9-fold) | RNA-seq | *S. aureus* | HG003 - laboratory strain | MB-aPDI (33 µM, 2.4 J/cm^2^) | > 3 log_10_ | [20] |
| *sepA* / multidrug resistance efflux pump | Involved in multidrug efflux | ↓ Downregulated  (6.14-fold for MSSA)  (3.47-fold for MRSA) | RT-qPCR | *S. aureus* | Newman (MSSA)  USA300 (MRSA) | TBO-aPDI (0.5 mM, 635 nm, 40 J/cm^2^) | 1-3 log_10_ | [134] |
| *whiB7* / transcriptional regulator | Transcription regulation via the DNA binding (The apo- but not holo-form probably binds DNA) | ↓ Downregulated  (approx. 4-fold at 40  mg/mL)  (approx. 10-fold at 100  mg/mL) | RT-qPCR | *M. abscessus* | ATCC 19977 | ALA-aPDI (40, 100 mg/mL, 80 J/cm^2^, 635 nm) | No statistically significant reduction (40 mg/mL )  < 1 log_10_ (100 mg/mL) | [130] |
| *vanA* / D-alanine–D-lactate ligase | Resistance to vancomycin | ↑ Upregulated  (2.8-fold – isolate A1)  (3.3-fold – isolate A2) | RT-qPCR | *E. faecalis* | ATTC 29212  A1, A2 – clinical isolates, Karolinska Hospital culture collections  UmID1, UmID2, UmID3 - infected dental root-canals, Umeå bacterial collection | TMPyP-aPDI (0.015 µM, 445 nm, 210 J/cm^2^) | 1-3 log_10_ | [77] |

* Authors did not provide fold change values

**Supplementary Table S6. Differentially expressed genes related to microbial cell envelope upon photodynamic treatment**.

| **Gene/encoded protein** | **Function** | **Expression change** | **Method** | **Species** | **Source of**  **bacteria** | **Photoinactivation** | **Decrease in viability** | **Refe-**  **rence** |
| --- | --- | --- | --- | --- | --- | --- | --- | --- |
| *atl* / peptidoglycan hydrolase | Hydrolysis of the link between N-acetylmuramoyl residues and L-amino acid residues in certain cell-wall glycopeptides | ↓ Downregulated  (1.72-fold) | RT-qPCR | *S. aureus* | MRSA252 | 415 nm aBL (54.72 J/cm^2^) | 1-3 log_10_ | [141] |
| *cap5I, cap5J*, c*ap5*K / capsular polysaccharide synthesis enzymes | Translocation of the ﬁnal precursor lipids to the outer cell surface - formation of a protective polysaccharide microcapsule | ↓ Downregulated  (3.36-fold, 4.29-fold,  4.23-fold) | RNA-seq | *S. aureus* | HG003 - laboratory strain | MB-aPDI (33 µM, 2.4 J/cm^2^) | > 3 log_10_ | [20] |
| *capA, capB*, *capC*, *capD*, *capF, capG, cap8K, capL* / capsular polysaccharide synthase | Production of capsular polysaccharide | ↑ Upregulated  (*) | RNA-seq | *S. aureus* | MRSA 8325-4, Wellman Center for Photomedicine, Boston  MRSA 252, ATCC | 460 nm aBL (120 J/cm^2^) | < 1 log_10_ | [116] |
| *crtN* / dehydrosqualene desaturase | Biosynthesis of staphyloxanthin | ↑ Upregulated  (*) | RNA-seq | *S. aureus* | MRSA 8325-4, Wellman Center for Photomedicine, Boston  MRSA 252, ATCC | 460 nm aBL (120 J/cm^2^) | < 1 log_10_ | [116] |
| *crtM* / dehydrosqualene synthase |  | ↑ Upregulated  (*) | RNA-seq | *S. aureus* | MRSA 8325-4, Wellman Center for Photomedicine, Boston  MRSA 252, ATCC | 460 nm aBL (120 J/cm^2^) | < 1 log_10_ | [116] |
| *dacA* / penicillin-binding protein 5 (PBP5) | Formation cell wall | ↑ Upregulated  (2.0-fold) | RT-qPCR | *E. coli* | BW25113, Keio Knockout  Parent Strain | 415 nm aBL (43.2 J/cm^2^) | < 1 log_10_ | [23] |
|  |  | ↑ Upregulated  (2.0-fold) |  |  |  | 409 nm aBL (9.36 J/cm^2^) | < 1 log_10_ |  |
| *fadL, fadJ, fadI, fadH, fadE, fadD* / fad regulon | Transportation of long-chain fatty acids across the bacterial cell membrane | ↑ Upregulated  (69.7-fold, 92-fold,  49.8-fold, 8.3-fold,  31.3-fold, 53-fold) | RNA-seq | *E. coli* | O157:H7 (C7927, apple cider isolate) | 465 nm aBL | No reduction (growth rate) | [145] |
| *glmS* / glutamine-fructose-6-phosphate aminotransferase | Catalyzes the first step in hexosamine metabolism, converting fructose-6P into glucosamine-6P – crucial for bacterial cell wall synthesis | ↑ Upregulated  (3.11-fold)  (2.68-fold) | RNA-seq and RT-qPCR | *S. aureus* | MRSA252 | 415 nm aBL (54.72 J/cm^2^) | 1-3 log_10_ | [141] |
| *lpsB* / lipooligosaccharide core glycosyltransferase | Glycosyltransferase activity | ↑ Upregulated  (31-fold in strain resistant  to colistin)  (9- fold in reference  strain) | RT-qPCR | *A. baumannii* | CR-XDR-AB –  burn-wound infection  ATCC 19606 – reference strain | TBO-aPDI (0.37 mg/mL, 630nm, 180 J/cm^2^) | No reduction (growth inhibition) | [45] |
| *mscL* / ion channel | Protection from cell lysis during acute downward shifts in the osmotic pressure on the cell | ↑ Upregulated  (approx. 7.5-fold for  DHMD-RAIM)  (approx. 22-fold for  DHMD-RAI2M | RT-qPCR | *E. coli* | DH5α – reference strain | 450 nm aBL (﻿10 W/m^2^) | No influence on growth | [126] |
| *murG* / UDP-N-acetylglucosamine--N-acetylmuramyl-(pentapeptide) pyrophosphoryl-undecaprenol N-acetylglucosamine transferase | Cell wall formation (peptidoglycan biosynthesis pathway) | ↑ Upregulated  (1.22-fold) | RT-qPCR | *S. aureus* | MRSA252 | 415 nm aBL (54.72 J/cm^2^) | 1-3 log_10_ | [141] |
| *ompA* / outer membrane protein | Porin | ↑ Upregulated  (approx. 10-fold) | RT-qPCR | *A. baumannii* | XDR isolate from burn wound infection | TBO-aPDI (0.01 mg/mL, 630 nm, 2000-4000 mW/cm^2^, 60 s) | No data | [81] |
|  |  | ↑ Upregulated  (3.27-fold) | RNA-seq |  | ATCC 19606 | 462 nm blue light (6-10 µmol photons · m^-2^ ·s^-1^) | No data | [49] |
| *pbpB* / peptidoglycan glycosyltransferase | Production of the peptidoglycan in the bacterial cell wall. | ↑ Upregulated  (2.21-fold) | RT-qPCR | *S. aureus* | MRSA252 | 415 nm aBL (54.72 J/cm^2^) | 1-3 log_10_ | [141] |
| *pmrA* / two-component system PmrAB | Colistin resistance via regulating the expression of a set of genes associated with lipid A modification and virulence | ↓ Downregulated  (6.1-fold) | RT-qPCR | *A. baumannii* | CR-PDR-AB - isolated from a burn patient | TBO-aPDI (﻿0.37 mg/mL, 630 nm, 180 J/cm^2^, 60s) | No data | [144] |
| *pmrB* / two-component system PmrAB |  | ↓ Downregulated  (4.9-fold) | RT-qPCR | *A. baumannii* | CR-PDR-AB - isolated from a burn patient | TBO-aPDI (﻿0.37 mg/mL, 630 nm, 180 J/cm^2^, 60s) | No data | [144] |
| SAR1973 / membrane protein | Unknown | ↑ Upregulated  (*) | RNA-seq | *S. aureus* | MRSA 8325-4, Wellman Center for Photomedicine, Boston  MRSA 252, ATCC | 460 nm aBL (120 J/cm^2^) | < 1 log_10_ | [116] |
| *srtB* / sortase | Transpeptidase that probably anchors surface proteins to the cell wall | ↑ Upregulated  (5.03-fold) | RNA-seq | *S. aureus* | BUSA2288 – MRSA clinical isolate from nasal passage | 465 nm blue light (250 J/cm²) | No data | [117] |
| *ssaA* / secretory antigen precursor SsaA | Relaxation of peptidoglycan crosslinking | ↓ Downregulated  (2.14-fold)  (3.99-fold) | RNA-seq and RT-qPCR | *S. aureus* | MRSA252 | 415 nm aBL (54.72 J/cm^2^) | 1-3 log_10_ | [141] |

* Authors did not provide fold change values

**Supplementary Table S7. Differentially expressed genes related to cell metabolism and other processes upon photodynamic treatment**.

| **Gene/encoded protein** | **Function** | **Expression change** | **Method** | **Species** | **Source of bacteria** | **Photoinactivation** | **Decrease in viability** | **Refe-rence** |
| --- | --- | --- | --- | --- | --- | --- | --- | --- |
| *acpD* / azoreductase | Reduction of azo dyes and xenobiotics | ↑ Upregulated  (28.06-fold) | RNA-Seq | *S. aureus* | BUSA2288 – MRSA clinical isolate from nasal passage | 465 nm blue light (250 J/cm^2^) | No data | [117] |
| *arcC2* / carbamate kinase | Kinase catalyzing ATP and ADP synthesis | ↑ Upregulated  (2.1-fold) | cDNA microarray | *S. aureus* | NCTC8325-4 (MSSA) | Ce_6_-aPDI (5 μM, 664 nm, 15 J/cm^2^) | No data | [163] |
| *arcD* / arginine/ornithine antiporter | Arginine uptake and ornithine export | ↑ Upregulated  (2.2-fold) | cDNA microarray | *S. aureus* | NCTC8325-4 (MSSA) | Ce_6_-aPDI (5 μM, 664 nm, 15 J/cm^2^) | No data | [163] |
| *argB* / acetylglutamate kinase | Arginine biosynthesis | ↓ Downregulated  T1 (4.82-fold)  T2 (3.39-fold) | RNA-Seq | *C. jejuni* | NCTC11168H | 405 nm aBL (T1-15 min, 7 J/cm^2^; T2- 30 min, 14 J/cm^2^) | No statistically significant reduction (T1)  1-3 log_10_ (T2) | [22] |
| *argC* / N-acetyl-gamma-glutamyl-phosphate reductase) |  | ↓ Downregulated  T1 (4.83-fold)  T2 (8.94-fold) | RNA-Seq | *C. jejuni* | NCTC11168H | 405 nm aBL (T1-15 min, 7 J/cm^2^; T2- 30 min, 14 J/cm^2^) | No statistically significant reduction (T1)  1-3 log_10_ (T2) | [22] |
| *asd* / semialdehyde dehydrogenase) | Conversion of aspartate semialdehyde to diaminopimelate | ↓ Downregulated  (5.54-fold) | RNA-Seq | *S. aureus* | BUSA2288 – MRSA clinical isolate from nasal passage | 465 nm blue light (250 J/cm^2^) | No data | [117] |
| *benP* / fenzoate transport porin | Benzoate transporter | ↑ Upregulated  (2.85-fold) | RNA-Seq | *A. baumanii* | ATCC 17978, clinical isolate, human infant | 469 nm blue light | No data | [47] |
| *blsA* / blue-light sensing protein | Regulation of gene expression, transcriptional regulator | ↑ Upregulated  (2.80-fold) | RT-qPCR | *A. baumannii* | CR-XDR-AB –  burn-wound infection  ATCC 19606 – reference strain | TBO-aPDI (0.37 mg/mL, 630nm, 180 J/cm^2^) | No reduction (growth inhibition) | [45] |
| *catA* / catechol 1,2-dioxygenase | Conversion of catechol into cis,cis-muconate | ↑ Upregulated  (4.88-fold) | RNA-Seq | *A. baumanii* | ATCC 17978, clinical isolate, human infant | 469 nm blue light | No data | [47] |
| *chuA* / haemin uptake system outer membrane receptor | Iron acquisition from heme | ↑ Upregulated  T1 (2.15-fold)  T2 (17.92-fold) | RNA-Seq | *C. jejuni* | NCTC11168H | 405 nm aBL (T1-15 min, 7 J/cm^2^; T2- 30 min, 14 J/cm^2^) | No statistically significant reduction (T1)  1-3 log_10_ (T2) | [22] |
| *chuB* / putative haemin uptake system permease protein | Heme binding and transport | ↑ Upregulated  T1 (1.72-fold)  T2 (4.44-fold) | RNA-Seq | *C. jejuni* | NCTC11168H | 405 nm aBL (T1-15 min, 7 J/cm^2^; T2- 30 min, 14 J/cm^2^) | No statistically significant reduction (T1)  1-3 log_10_ (T2) | [22] |
| *cj0264c* / molybdopterin containing oxidoreductase | Anaerobic respiration | ↑ Upregulated  T1 (4.37-fold)  T2 (4.07-fold) | RNA-Seq | *C. jejuni* | NCTC11168H | 405 nm aBL (T1-15 min, 7 J/cm^2^; T2- 30 min, 14 J/cm^2^) | No statistically significant reduction (T1)  1-3 log_10_ (T2) | [22] |
| *cj0*265c / cytochrome C-type heme-binding protein |  | ↑ Upregulated  T1 (6.90-fold)  T2 (8.40-fold) | RNA-Seq | *C. jejuni* | NCTC11168H | 405 nm aBL (T1-15 min, 7 J/cm^2^; T2- 30 min, 14 J/cm^2^) | No statistically significant reduction (T1)  1-3 log_10_ (T2) | [22] |
| *dcuA* / anaerobic C4-dicarboxylate transporter | Transporter facilitating fumarate, succinate, and malate exchange | ↑ Upregulated  T1 (3.72-fold)  T2 (2.44-fold) | RNA-Seq | *C. jejuni* | NCTC11168H | 405 nm aBL (T1-15 min, 7 J/cm^2^; T2- 30 min, 14 J/cm^2^) | No statistically significant reduction (T1)  1-3 log_10_ (T2) | [22] |
| *dcuB* / anaerobic C4-dicarboxylate transporter | Transporter facilitating fumarate, succinate, and malate exchange | ↑ Upregulated  T1 (2.04-fold)  T2 (2.17-fold) | RNA-Seq | *C. jejuni* | NCTC11168H | 405 nm aBL (T1-15 min, 7 J/cm^2^; T2- 30 min, 14 J/cm^2^) | No statistically significant reduction (T1)  1-3 log_10_ (T2) | [22] |
| *efeO* / iron uptake system component protein | Iron acquisition and metabolism | ↑ Upregulated  (3.81-fold) | RNA-Seq | *S. aureus* | HG003 - laboratory strain | MB-aPDI (33 µM, 2.4 J/cm^2^) | > 3 log_10_ | [20] |
| *fdhF* / formate dehydrogenase H | Catalytic subunit of formate dehydrogenase H | ↓ Downregulated  (30-fold) | RNA-Seq | *E. coli* | ATCC 25922, German Collection of Microorganisms and Cell Cultures GmbH | SAPYR-aPDI (0.5 μM, 380–600 nm, 30 J/cm^2^) | < 1 log_10_ | [100] |
| *feABC* / ATP-binding cassette (ABC) transporter | Inorganic ions transport and metabolism | ↑ Upregulated  (4.60-fold) | RNA-Seq | *S. aureus* | HG003 - laboratory strain | MB-aPDI (33 µM, 2.4 J/cm^2^) | > 3 log_10_ | [20] |
| *fetB* / iron export permease protein FetB | Iron transport system component | ↑ Upregulated  (2.39-fold) | RT-qPCR | *P. gingivalis* | W83, Oral Microbiology Laboratory of Peking University School of Stomatology | 405 nm aBL (100 mW/cm^2^, 4 min) | > 3 log_10_ | [21] |
| *fieF* / ferrous-iron efflux pump | Metal cation transporters (such as Zn^2+^, Cd^2+^ and Fe^2^ in a proton-dependent manner | ↓ Downregulated   (14.8-fold) | RT-qPCR | *A. actinomycetemco*  *mitans* | ATCC 33384 | CNPs/ICG-aPDI (810 nm, 200 mW, 31.2 J/cm^2^) | < 1 log_10_ | [159] |
| *fimA* / fimbrilin | Adhesion onto host cells, biofilm formation, stimulation host immune response | ↓ Downregulated  (10.4-fold for 1/2 × MIC) | RT-qPCR | *P. gingivalis* | IR-TUMS/BPG5, Genbank: KX108929.1 | DNA-aptamer-NGO-aPDI (1/2 × MIC, 980 nm, 1W) | > 3 log_10_ | [153] |
| *frd* / fumarate reductase iron-sulfur protein | Conversion of fumarate to succinate in anaerobic | ↑ Upregulated  T1 (1.23-fold)  T2 (1.21-fold) | RNA-Seq | *C. jejuni* | NCTC11168H | 405 nm aBL (T1-15 min, 7 J/cm^2^; T2- 30 min, 14 J/cm^2^) | No statistically significant reduction (T1)  1-3 log_10_ (T2) | [22] |
| *ftn* / ferritin-like protein | Iron storage | ↑ Upregulated  (3.10-fold) | RT-qPCR | *P. gingivalis* | W83, Oral Microbiology Laboratory of Peking University School of Stomatology | 405 nm aBL (100 mW/cm^2^, 4 min) | > 3 log_10_ | [21] |
| *fumB* / fumarate hydratase | Conversion of fumarate to malate under anaerobic conditions. | ↓ Downregulated  (30-fold) | RNA-Seq | *E. coli* | ATCC 25922, German Collection of Microorganisms and Cell Cultures GmbH | SAPYR-aPDI (0.5 μM, 380–600 nm, 30 J/cm^2^) | < 1 log_10_ | [100] |
| *geh* / staphylococcal lipase | Triglycerides hydrolisys into glycerol and free fatty acids. | ↑ Upregulated  (12.1-fold) | cDNA microarray | *S. aureus* | NCTC8325-4 (MSSA) | Ce_6_-aPDI (5 μM, 664 nm, 15 J/cm^2^) | No data | [163] |
| *htsB* / heme ABC type transporter permease | Heme iron acquisition | ↑ Upregulated  (2.96-fold) | RNA-Seq | *S. aureus* | HG003 - laboratory strain | MB-aPDI (33 µM, 2.4 J/cm^2^) | > 3 log_10_ | [20] |
| *hutI* / imidazolonepropionase | Histidine degradation by imidazolonepropionate to N-formimino-L-glutamate conversion | ↑ Upregulated  (5.2-fold) | cDNA microarray | *S. aureus* | NCTC8325-4 (MSSA) | Ce_6_-aPDI (5 μM, 664 nm, 15 J/cm^2^) | No data | [163] |
| *hutU* / urocanate hydratase | Conversion of urocanate to 4-imidazolone-5-propionate in histidine metabolism. | ↑ Upregulated  (6.7-fold) | cDNA microarray | *S. aureus* | NCTC8325-4 (MSSA) | Ce_6_-aPDI (5 μM, 664 nm, 15 J/cm^2^) | No data | [163] |
| *hycB* / formate hydrogenlyase subunit 2 | Electron transfer protein in the FHL | ↓ Downregulated  (52-fold) | RNA-Seq | *E. coli* | ATCC 25922, German Collection of Microorganisms and Cell Cultures GmbH | SAPYR-aPDI (0.5 μM, 380–600 nm, 30 J/cm^2^) | < 1 log_10_ | [100] |
| *hycC* / formate hydrogenlyase subunit 3 | Membrane-bound electron transport protein in the FHL | ↓ Downregulated  (34.3-fold) | RNA-Seq | *E. coli* | ATCC 25922, German Collection of Microorganisms and Cell Cultures GmbH | SAPYR-aPDI (0.5 μM, 380–600 nm, 30 J/cm^2^) | < 1 log_10_ | [100] |
| *hycF* / formate hydrogenlyase subunit 6 | Regulatory protein modulating FHL activity. | ↓ Downregulated  (32-fold) | RNA-Seq | *E. coli* | ATCC 25922, German Collection of Microorganisms and Cell Cultures GmbH | SAPYR-aPDI (0.5 μM, 380–600 nm, 30 J/cm^2^) | < 1 log_10_ | [100] |
| *hydN* / electron transport protein HydN | Putative electron transport protein | ↓ Downregulated  (45.3-fold) | RNA-Seq | *E. coli* | ATCC 25922, German Collection of Microorganisms and Cell Cultures GmbH | SAPYR-aPDI (0.5 μM, 380–600 nm, 30 J/cm^2^) | < 1 log_10_ | [100] |
| *kdtA* / 3-deoxy-D-manno-octulosonic-acid | LPS biosynthesis | ↓ Downregulated  T1 (7.15-fold)  T2 (11.49-fold) | RNA-Seq | *C. jejuni* | NCTC11168H | 405 nm aBL (T1-15 min, 7 J/cm^2^; T2- 30 min, 14 J/cm^2^) | No statistically significant reduction (T1)  1-3 log_10_ (T2) | [22] |
| *lysC* / aspartate kinase | Biosynthesis of lysine, methionine, and threonine | ↓ Downregulated  (5.3-fold) | RNA-Seq | *S. aureus* | BUSA2288 – MRSA clinical isolate from nasal passage | 465 nm blue light (250 J/cm^2^) | No data | [117] |
| *mfrA* / periplasmic fumarate reductase flavoprotein subunit | Catalyzes the reduction of fumarate to succinate under anaerobic conditions. | ↑ Upregulated  T1 (2.15-fold)  T2 (17.92-fold) | RNA-Seq | *C. jejuni* | NCTC11168H | 405 nm aBL (T1-15 min, 7 J/cm^2^; T2- 30 min, 14 J/cm^2^) | No statistically significant reduction (T1)  1-3 log_10_ (T2) | [22] |
| *mfrB* / fumarate reductase iron-sulfur protein |  | ↑ Upregulated  T1 (4.84-fold)  T2 (4.30-fold) | RNA-Seq | *C. jejuni* | NCTC11168H | 405 nm aBL (T1-15 min, 7 J/cm^2^; T2- 30 min, 14 J/cm^2^) | No statistically significant reduction (T1)  1-3 log_10_ (T2) | [22] |
| *mfrE* / periplasmic fumarate reductase subunit E |  | ↑ Upregulated  T1 (2.22-fold)  T2 (2.22-fold) | RNA-Seq | *C. jejuni* | NCTC11168H | 405 nm aBL (T1-15 min, 7 J/cm^2^; T2- 30 min, 14 J/cm^2^) | No statistically significant reduction (T1)  1-3 log_10_ (T2) | [22] |
| *napG* / putative ferrodoxin | Electron transfer components of periplasmic nitrate reductase | ↑ Upregulated  T1 (2.08-fold)  ↓ Downregulated  T2 (1.07-fold) | RNA-Seq | *C. jejuni* | NCTC11168H | 405 nm aBL (T1-15 min, 7 J/cm^2^; T2- 30 min, 14 J/cm^2^) | No statistically significant reduction (T1)  1-3 log_10_ (T2) | [22] |
| *napH* / putative ferrodoxin | Electron transfer components of periplasmic nitrate reductase | ↑ Upregulated  T1 (2.87-fold)  T2 (1.65-fold) | RNA-Seq | *C. jejuni* | NCTC11168H | 405 nm aBL (T1-15 min, 7 J/cm^2^; T2- 30 min, 14 J/cm^2^) | No statistically significant reduction (T1)  1-3 log_10_ (T2) | [22] |
| *pckA* / phosphoenolpyruvate carboxykinase | Oxaloacetate conversion to phosphoenolpyruvate in gluconeogenesis | ↑ Upregulated  (4.8-fold) | RNA-Seq | *S. aureus* | NCTC8325-4 (MSSA) | Ce_6_-aPDI (5 μM, 664 nm, 15 J/cm^2^) | No data | [163] |
| *PcpA_N-like* / dioxygenase | Oxidative degradation of aromatic compounds | ↑ Upregulated  (18.32-fold) | RNA-Seq | *S. aureus* | BUSA2288 – MRSA clinical isolate from nasal passage | 465 nm blue light (250 J/cm^2^) | No data | [117] |
| *rgpA* / arginine-specific gingipain (cysteine protease) | Generation of peptides or amino acids as energy and carbon source; Processing mechanisms for bacterial surface proteins; Heme uptake, conversion of oxyhemoglobin to methemoglobin | ↓ Downregulated   (6.8-fold for 1/2 × MIC)  ↓ Downregulated   (4.1-fold for 1/4 × MIC) | RT-qPCR | *P. gingivalis* | IR-TUMS/BPG5, Genbank: KX108929.1 | DNA-aptamer-NGO-aPDI (1/2 × MIC, 1/4 × MIC, 980 nm, 1W) | > 3 log_10_ | [153] |
|  |  | ↓ Downregulated   (11.6-fold) | RT-qPCR | *P. gingivalis* | Clinical isolates from root canal from patients treated with PAD | TBO-aPDI (6.25 μg/ml, 171.87 J/cm^2^, 635 nm, 220 mW) | No statistically significant reduction | [155] |
|  |  | ↓ Downregulated   (14.0-fold) | RT-qPCR | *P. gingivalis* |  | ICG-aPDI (15.6 μg/mL, 15.6 J/cm^2^, 810 nm, 250 mW) | No statistically significant reduction |  |
|  |  | ↓ Downregulated   (4.9-fold) | RT-qPCR | *P. gingivalis* |  | MB-aPDI (25 μg/mL, 93.75 J/cm^2^, 660 nm, 150 mW) | No statistically significant reduction |  |
|  |  | ↑ Upregulated  (7.41-fold) | RT-qPCR | *P. gingivalis* | W83, Oral Microbiology Laboratory of Peking University School of Stomatology | 405 nm aBL (100 mW/cm^2^, 4 min) | > 3 log_10_ | [21] |
| *rgpB* / arginine-specific gingipain (cysteine protease) |  | ↑ Upregulated  (8.60-fold) | RT-qPCR | *P. gingivalis* | W83, Oral Microbiology Laboratory of Peking University School of Stomatology | 405 nm aBL (100 mW/cm^2^, 4 min) | > 3 log_10_ | [21] |

**Supplementary Table S8. Differentially expressed genes related to potential mechanisms involved in light tolerance.**

| **Gene/encoded protein** | **Function** | **Expression change** | **Method** | **Species** | **Source of bacteria** | **Photoinactivation** | **Decrease in viability** | **Refe-rence** |
| --- | --- | --- | --- | --- | --- | --- | --- | --- |
| *ahpC* / alkyl hydroperoxide reductase C) | Organic peroxides reduction | ↓ Downregulated  (approx. 2.0-fold) | RT-qPCR | *S. agalactiae* | ATTC 27956 - isolated from infected bovine udder | RB-aPDI (0.08 μM, 515 nm, 20 J/cm^2^) | ≤ 1 log_10_ | [94] |
| *cylE* / cytolysin E | Hemolysin activity | ↓ Downregulated  (approx. 5-fold) | RT-qPCR | *S. agalactiae* | ATTC 27956 - isolated from infected bovine udder | RB-aPDI (0.08 μM, 515 nm, 20 J/cm^2^) | ≤ 1 log_10_ | [94] |
| *csoR* / copper-sensing transcriptional repressor CsoR | Cellular response to increasing concentrations of copper inside the bacterium | ↓ Downregulated  (1.69-fold) | RNA-seq | *S. aureus* | HG003 - laboratory strain | MB-aPDI (33 µM, 2.4 J/cm^2^) | > 3 log_10_ | [20] |

| *htsA* / heme ABC type transporter HtsABC | Heme transporter necessary for iron acquisition. | ↑ Upregulated  (1.99-fold) | RNA-seq | *S. aureus* | HG003 - laboratory strain | MB-aPDI (33 µM, 2.4 J/cm^2^) | > 3 log_10_ | [20] |
| --- | --- | --- | --- | --- | --- | --- | --- | --- |
| *htsB* / heme ABC type transporter HtsABC, permease protein HtsB |  | ↑ Upregulated  (2.96-fold) | RNA-seq | *S. aureus* | HG003 - laboratory strain | MB-aPDI (33 µM, 2.4 J/cm^2^) | > 3 log_10_ | [20] |
| *htsC* / heme ABC type transporter HtsABC, |  | ↑ Upregulated  (2.39-fold) | RNA-seq | *S. aureus* | HG003 - laboratory strain | MB-aPDI (33 µM, 2.4 J/cm^2^) | > 3 log_10_ | [20] |
| *mgrA* / HTH-type transcriptional regulator MgrA | Regulatory protein involved in autolytic activity, multidrug resistance and virulence | ↑ Upregulated  (1.73-fold) | RNA-seq | *S. aureus* | HG003 - laboratory strain | MB-aPDI (33 µM, 2.4 J/cm^2^) | > 3 log_10_ | [20] |
| *npx* / NADH peroxidase | Reduction of hydrogen peroxide (H₂O₂) to water using NADH as an electron donor | ↑ Upregulated  (approx. 20-fold for  consecutive aPDI) | RT-qPCR | *S. agalactiae* | ATTC 27956 - isolated from infected bovine udder | RB-aPDI (0.08 μM, 515 nm, 20 J/cm^2^) | ≤ 1 log_10_ | [94] |
| *oxyR* / oxidative stress response regulator | Antioxidant defense genes regulation | ↑ Upregulated  (1.5-fold) | RT-qPCR | *S. Enteritidis* | ATCC 13076 (CDC) | 405 nm aBL (72 J/cm^2^, 4°C) | No statistically significant reduction | [18] |
|  |  | ↓ Downregulated  (1.4-fold) |  | *S. Saintpaul* | ATCC 9712 (cystitis, Panama) |  | No statistically significant reduction |  |
| *recA* / recombinase A | DNA repair, SOS response | ↑ Upregulated  (approx. 2.5-fold for  single aPDI)  (approx. 20-fold for consecutive aPDI) | RT-PCR | *S. agalactiae* | ATTC 27956 - isolated from infected bovine udder | RB-aPDI (0.08 μM, 515 nm, 20 J/cm^2^) | ≤ 1 log_10_ | [94] |
| *saeS* / sensor histidine protein kinase SaeS | Member of the two-component regulatory system SaeR/SaeS involved in the regulation of staphylococcal virulence factors | ↑ Upregulated  (2.0-fold) | RNA-seq | *S. aureus* | HG003 - laboratory strain | MB-aPDI (33 µM, 2.4 J/cm^2^) | > 3 log_10_ | [20] |
| SAOUHSC_00093 / manganese-dependent superoxide dismutase | Catalyze the dismutation of the free radical superoxide anion (O_2_^.^−) to H_2_O_2_ and molecular oxygen | ↑ Upregulated  (2.95-fold) | RNA-seq | *S. aureus* | HG003 - laboratory strain | MB-aPDI (33 µM, 2.4 J/cm^2^) | > 3 log_10_ | [20] |
| SAOUHSC_00173 / flavin mononucleotide (FMN)-dependent NADH-azoreductase | Catalyzes the reduction of azo groups in aromatic azo compounds and indigo compounds | ↑ Upregulated  (2.57-fold) | RNA-seq | *S. aureus* | HG003 - laboratory strain | MB-aPDI (33 µM, 2.4 J/cm^2^) | > 3 log_10_ | [20] |
| SAOUHSC_00318 / glyoxalase/bleomycin resistance protein | Part of the Qsr regulon, which controls the expression of antioxidant enzymes | ↓ Downregulated  (1.38-fold) | RNA-seq | *S. aureus* | HG003 - laboratory strain | MB-aPDI (33 µM, 2.4 J/cm^2^) | > 3 log_10_ | [20] |
| SAOUHSC_00320 / NADH-dependent flavin mononucleotide reductase | Flavin reductases catalyze the reduction of flavin through NADH or NADPH oxidation | ↓ Downregulated  (1.90-fold) | RNA-seq | *S. aureus* | HG003 - laboratory strain | MB-aPDI (33 µM, 2.4 J/cm^2^) | > 3 log_10_ | [20] |
| SAOUHSC_00833 / nitroreductase family protein | Metabolism of nitrosubstituted compounds - reduce aromatic nitro groups to hydroxylamine and groups amines | ↑ Upregulated  (1.55-fold) | RNA-seq | *S. aureus* | HG003 - laboratory strain | MB-aPDI (33 µM, 2.4 J/cm^2^) | > 3 log_10_ | [20] |
| SAOUHSC_02825 / glyoxalase family protein | Detoxification against methylglyoxal and other aldehydes, the metabolites derived from glycolysisD | ↓ Downregulated  (1.83-fold) | RNA-seq | *S. aureus* | HG003 - laboratory strain | MB-aPDI (33 µM, 2.4 J/cm^2^) | > 3 log_10_ | [20] |
| *sirA*, *sirB* / siderophore-binding protein SirA and SirB | Iron-regulated ABC transporter | ↑ Upregulated  (1.71-fold), (1.61-fold) | RNA-seq | *S. aureus* | HG003 - laboratory strain | MB-aPDI (33 µM, 2.4 J/cm^2^) | > 3 log_10_ | [20] |
| *sodA* /superoxide dismutase A | Superoxide radicals neutralization | ↑ Upregulated  (approx. 5.0-fold for  single aPDI)  (approx. 20-fold for  consecutive aPDI) | RT-qPCR | *S. agalactiae* | ATTC 27956 - isolated from infected bovine udder | RB-aPDI (0.08 μM, 515 nm, 20 J/cm^2^) | ≤ 1 log_10_ | [94] |
|  |  | ↑ Upregulated  (13.5-fold for 472 strain)  (20.0-fold for 80/0 strain)  No significant change for 2002 and 4246 strains | RT-qPCR | *S. aureus* | 472, 80/0, 2002, 4246 – MRSA clinical strain from hospital patients | PPIX-aPDI (50 μM, 624 nm, 12 J/cm^2^) | > 3 log_10_ (472 strain)  1-3 log_10_ (80/0 strain)  < 1 log_10_ (2002 and 4246 strains) | [176] |
| *sodM* / superoxide dismutase | Detoxfication | ↑ Upregulated  (41-fold for 472 strain)  (4.1-fold for 80/0 strain)  No significant change for 2002 and 4246 strains | RT-qPCR | *S. aureus* | 472, 80/0, 2002, 4246 – MRSA clinical strain from hospital patients | PPIX-aPDI (50 μM, 624 nm, 12 J/cm^2^) | > 3 log_10_ (472 strain)  1-3 log_10_ (80/0 strain)  < 1 log_10_ (2002 and 4246 strains) | [176] |
| *tpx* / thiol peroxidase | Reduction of hydrogen peroxide and organic hydroperoxides | ↑ Upregulated  (approx. 2.5-fold for  single aPDI)  (approx. 3.0-fold for  consecutive aPDI) | RT-qPCR | *S. agalactiae* | ATTC 27956 - isolated from infected bovine udder | RB-aPDI (0.08 μM, 515 nm, 20 J/cm^2^) | ≤ 1 log_10_ | [94] |
